# Albuminoid Genes: Evolving at the Interface of Dispensability and Selection

**DOI:** 10.1093/gbe/evu235

**Published:** 2014-10-27

**Authors:** Alessandra Mozzi, Diego Forni, Rachele Cagliani, Uberto Pozzoli, Jacopo Vertemara, Nereo Bresolin, Manuela Sironi

**Affiliations:** ^1^Bioinformatics, Scientific Institute IRCCS E.MEDEA, Bosisio Parini, Italy; ^2^Dino Ferrari Centre, Department of Physiopathology and Transplantation, University of Milan, Fondazione Ca’ Granda IRCCS Ospedale Maggiore Policlinico, Milano, Italy

**Keywords:** albumin, vitamin D-binding protein, albuminoids, positive selection, vitamin D, hibernation

## Abstract

The albuminoid gene family comprises vitamin D-binding protein (*GC*), alpha-fetoprotein (*AFP*), afamin (*AFM*), and albumin (*ALB*). Albumin is the most abundant human serum protein, and, as the other family members, acts as a transporter of endogenous and exogenous substances including thyroxine, fatty acids, and drugs. Instead, the major cargo of GC is 25-hydroxyvitamin D. We performed an evolutionary study of albuminoid genes and we show that *ALB* evolved adaptively in mammals. Most positively selected sites are located within albumin-binding sites for fatty acids and thyroxine, as well as at the contact surface with neonatal Fc receptor. Positive selection was also detected for residues forming the prostaglandin-binding pocket. Adaptation to hibernation/torpor might explain the signatures of episodic positive selection we detected for few mammalian lineages. Application of a population genetics–phylogenetics approach showed that purifying selection represented a major force acting on albuminoid genes in both humans and chimpanzees, with the strongest constraint observed for human *GC*. Population genetic analysis revealed that *GC* was also the target of locally exerted selective pressure, which drove the frequency increase of different haplotypes in distinct human populations. A search for known variants that modulate GC and 25-hydroxyvitamin D concentrations revealed linkage disequilibrium with positively selected variants, although European and Asian major *GC* haplotypes carry alleles with reported opposite effect on GC concentration. Data herein indicate that albumin, an extremely abundant housekeeping protein, was the target of pervasive and episodic selection in mammals, whereas *GC* represented a selection target during the recent evolution of human populations.

## Introduction

The albumin gene family comprises four genes encoding vitamin D-binding protein (official gene symbol: *GC*, group-specific component), albumin (*ALB*), alpha-fetoprotein (*AFP*), and afamin (*AFM*). These genes are developmentally regulated, mainly expressed in the liver, and the proteins are secreted into the bloodstream (Gibbs et al. 1998). Albuminoids are serum proteins essential for the transport of a wide range of molecules, which are made available beyond their solubility in plasma.

The linear chromosomal arrangement of the four genes and their structural similarities testify a common ancestry. Starting with the first duplication of an ancestral progenitor gene, a single evolutionary line gave rise to *GC* and to the *ALB/AFP/AFM* precursor. The second duplication occurred in this lineage, giving rise to *ALB* and to the *AFP/AFM* ancestor; this latter more recently duplicated to originate the *AFP* and *AFM* genes (Nishio et al. 1996; Gibbs et al. 1998).

Albumin is the most abundant protein in extracellular fluids and its high concentration (around 0.6 mM in plasma) contributes to colloid osmotic pressure maintenance (Curry 2009). Albumin is an important lipid carrier, as it displays multiple binding sites for medium- and long-chain fatty acids. It also acts as a plasma depot for thyroxine, a thyroid hormone mainly responsible for metabolic control (Hulbert 2000), and it can bind heme and bilirubin molecules. The conformational adaptability and the allosteric fatty acids-induced regulation facilitate the binding of a broad range of pharmaceutical drugs, affecting their availability and biological efficacy (Curry 2009). Moreover, albumin shows antioxidant functions, acting as radical scavenger and sequestering metal ions and nitric oxide (Fasano et al. 2005), as well as enzymatic properties for prostaglandins (Curry 2009; Yamaguchi et al. 2010). Despite its multiple roles and high abundance in serum, congenital analbuminemia (CAA), although rare, is compatible with life (Koot et al. 2004; Toye et al. 2012).

Alpha-fetoprotein is considered the fetal counterpart of albumin, although it is also detectable in small amounts in adults. As albumin, it binds a wide variety of hydrophobic ligands including fatty acids, bilirubin, retinoids, flavonoids, chemical drugs, and metal ions (Terentiev and Moldogazieva 2013). AFP also transports estrogens, regulating their concentration during embryonic development (Terentiev and Moldogazieva 2013).

The third member of the albumin family gene encodes afamin, which shows high affinity binding for vitamin E, possibly representing its major carrier in body fluids (Voegele et al. 2002). Vitamin E is involved in many neurological and immunological processes, contributes to biological membrane stabilization, and shows antioxidant properties (Borel et al. 2013).

Finally, vitamin D-binding protein is the main carrier for vitamin D and its metabolites, known to be essential in the development, function, and maintenance of healthy bones through the regulation of calcium homeostasis (Speeckaert et al. 2006). *Gc^–/−^* mice are viable and healthy, but more susceptible to vitamin D deficiency (Safadi et al. 1999). In humans, vitamin D synthesis is mainly sun-induced and influenced by season, time of day, latitude, altitude, air pollution, skin pigmentation, and aging (Wacker and Holick 2013).

Therefore, albuminoid proteins are involved in central homeostatic functions. Although albumin is one of the most extensively investigated proteins, few studies have analyzed its evolutionary history, and most of these mainly aimed at reconstructing the duplication/divergence events that originated the four family member genes (Gibbs et al. 1998; Ascenzi et al. 2013). Herein, we analyzed the evolutionary history of albuminoid genes at inter- and intraspecific levels.

## Materials and Methods

### Evolutionary Analysis in Mammals

Mammalian coding sequences for *GC*, *AFM*, *AFP*, and *ALB* genes were retrieved from the Ensembl and National Center for Biotechnology Information databases. The list of species for each gene is reported in supplementary table S1, Supplementary Material online. All sequences were translated and checked against the GenBank data set through protein BLAST (Basic Local Alignment Search Tool) (http://blast.ncbi.nlm.nih.gov/, last accessed September 20, 2014); differences (always very minor) were manually parsed against the vertebrate Multiz Alignments available through the UCSC (University of California–Santa Cruz) Genome Browser (http://genome-euro.ucsc.edu/, last accessed September 20, 2014) and the nucleotide coding sequence corrected accordingly. DNA alignments were performed using the RevTrans 2.0 utility (Wernersson and Pedersen 2003). All of them were first checked by the use of trimAl (automated1 mode) (Capella-Gutierrez et al. 2009); subsequently, alignments were visually inspected: Manual editing was only used to correct few misalignments in proximity of small gaps (supplementary fig. S1, Supplementary Material online).

Alignments were screened for the presence of recombination breakpoints using GARD (genetic algorithm recombination detection) (Kosakovsky Pond et al. 2006).

To detect selection, *codeml* NSsite models were fitted to the data using different models of equilibrium codon frequencies (Yang 1997, 2007). These models treat the d*N*/d*S* (ω) ratio for any codon in the gene as a random variable from a statistical distribution, thus allowing ω to vary from site to site, assuming a constant rate at synonymous sites. Two models of equilibrium codon frequencies were used: The F3 × 4 model (codon frequencies estimated from the nucleotide frequencies in the data at each codon site) and the F61 model (frequencies of each of the 61 nonstop codons estimated from the data) (Yang 1997, 2007). Likelihood ratio test (LRT) analyses were performed either for whole gene alignments or independently for subregions defined in accordance with the recombination breakpoints. In these latter cases, Bonferroni correction for multiple tests was applied to the LRT *P* values.

Specific sites under selection were identified using Bayes Empirical Bayes (BEB) analysis from the M8 model with a significance cutoff of 0.90 (Anisimova et al. 2002; Yang et al. 2005).

A second method, MEME (Mixed Effects Model of Evolution) (with the default cutoff of 0.1) (Murrell et al. 2012) was also applied to identify positively selected sites. MEME allows the distribution of ω to vary from site to site and from branch to branch at a site, thus detecting of both pervasive and episodic positive selection.

To explore possible variations in selective pressure among different lineages, we applied the free-ratio models implemented in the PAML package: The M0 model assumes all branches to have the same ω, whereas M1 allows each branch to have its own ω (Yang and Nielsen 1998).

In order to identify specific branches with a proportion of sites evolving with ω > 1, we used branch site-random effects likelihood (BS-REL). The method applies sequential LRTs to identify significant branches without a priori knowledge about which lineages are of interest (Kosakovsky Pond et al. 2011); branches identified using this approach were cross-validated using the branch-site LRTs from PAML (models MA and MA1). A false discovery rate (FDR) correction was applied to account for multiple hypothesis testing, as previously suggested (Anisimova and Yang 2007). BEB analysis from MA (with a cutoff of 0.90) was used to identify sites that evolved under positive selection on specific lineages.

GARD (Kosakovsky Pond et al. 2006), MEME (Murrell et al. 2012), SLAC (single-likelihood ancestor counting) (Kosakovsky Pond and Frost 2005), and BS-REL analyses were performed either through the DataMonkey server (Delport et al. 2010) (http://www.datamonkey.org, last accessed June 20, 2014) or run locally (through HyPhy).

### Population Genetics–Phylogenetics Analysis

Data from the Pilot 1 phase of the 1000 Genomes (1000 G) Project were retrieved from the dedicated website (http://www.1000genomes.org/, last accessed September 20, 2014) (1000 Genomes Project Consortium et al. 2010). For chimpanzees, we used phased single nucleotide polymorphisms (SNPs) information of ten *Pan troglodytes verus* (Auton et al. 2012); ancestral sequences were reconstructed by parsimony from the human, chimpanzee, orangutan, and macaque sequences.

For gammaMap analysis (Wilson et al. 2011), we assumed θ (neutral mutation rate per site), k (transitions/transversions ratio), and T (branch length) to vary among genes following log-normal distributions. For each gene, we set the neutral frequencies of non-STOP codons (1/61) and the probability that adjacent codons share the same selection coefficient (*P* = 0.02). For selection coefficients, we considered a uniform Dirichlet distribution with the same prior weight for each selection class. For each gene, we run 10,000 iterations with thinning interval of ten iterations.

### Protein Alignment and 3D Structure Analysis

The multiple protein alignment of human albumin, alpha-fetoprotein, and afamin was performed using ClustalW (Larkin et al. 2007). Protein three-dimensional (3D) structures for human albumin (1HK4, 1HK1, and 4K71) were derived from the Protein Data Bank (PDB). Sites were mapped onto structures using PyMOL (The PyMOL Molecular Graphics System, Version 1.5.0.2; Schrödinger, LLC).

### Population Genetics Analyses

Data from the 1000 G Pilot Project were retrieved from the dedicated website (http://www.1000genomes.org/, last accessed September 20, 2014) (1000 Genomes Project Consortium et al. 2010). SNP genotypes were organized in a MySQL database. A set of programs was developed to retrieve genotypes from the database and to analyze them according to selected genomic regions/populations. These programs were developed in C++ using the GeCo++ (Cereda et al. 2011) and the libsequence (Thornton 2003) libraries.

Genotype information was obtained for the four albuminoid genes; a control set of 3,000 genes was used as a reference. These data were used to calculate θ_W_ (Watterson 1975), π (Nei and Li 1979), as well as Tajima’s *D* (Tajima 1989)*.* Fay and Wu’s *H* (DH) (Fay and Wu 2000; Zeng et al. 2006) was also calculated in 5-kb sliding windows moving with a step of 500 bp. Sliding window analyses have an inherent multiple testing problem that is difficult to correct because of the nonindependence of windows. In order to partially account for this limitation, we applied the same procedure to the control gene set, and the distribution of DH was obtained for the corresponding windows. This allowed calculation of the fifth percentile and visualization of regions below this threshold.

*F*_ST_ (Wright 1950) and the DIND (Derived Intra-allelic Nucleotide Diversity) test (Barreiro et al. 2009) were calculated for all SNPs mapping to the control and albuminoid gene sets. Because *F*_ST_ values are not independent from allele frequencies, we binned variants based on their minor allele frequency (MAF, 50 classes) and calculated the percentiles for each MAF class. As for the DIND test, it was originally developed for application to Sanger or high coverage sequencing data (Barreiro et al. 2009), so that statistical significance can be inferred through coalescent simulations. This is not the case for the 1000 G Project data; thus, we calculated statistical significance by obtaining an empirical distribution of DIND–derived allele frequency (DAF) value pairs for variants located within control genes. Specifically, DIND values were calculated for all SNPs using a constant number of 40 flanking variants (20 up- and downstream), as previously described (Forni et al. 2013, 2014). The distributions of DIND–DAF pairs for Yoruba (YRI), Europeans (CEU), and Chinese plus Japanese (CHBJPT) were binned in DAF intervals (100 classes) and for each class the percentiles were calculated. As suggested previously (Barreiro et al. 2009), for values of iπ_D_ = 0 we set the DIND value to the maximum obtained over the whole data set plus 20. Due to the nature of low-coverage data, for low DAF values most iπ_D_ resulted equal to 0 (i.e., the 95th percentile could not be calculated); thus, we did not calculate DIND in these ranges and we consequently cannot detect selection acting on low frequency derived alleles.

## Results

### The Albumin Gene Evolved Adaptively in Mammals, with Different Selective Pressure among Lineages

To analyze the evolutionary history of albuminoid genes in mammals, we obtained and aligned coding sequence information for all species available in public databases. For each gene, at least 49 species were available, including Metatheria and Eutheria, and roughly covering 175 Myr of mammalian history (supplementary table S1, Supplementary Material online) (Madsen 2009).

Recombination may introduce apparent substitution rate heterogeneity among sites (Worobey 2001) increasing type I error rates when models of positive selection are applied (Anisimova et al. 2003); thus, we screened the four alignments for the presence of recombination breakpoints using GARD (Kosakovsky Pond et al. 2006). GARD detected one breakpoint in *ALB* and *AFP* and two breakpoints in the *AFM* gene, whereas no evidence of recombination was detected for *GC* (table 1)*.* We next calculated the average nonsynonymous substitution/synonymous substitution rate ratio (d*N*/d*S*, also referred to as ω) for the four genes using the SLAC method (Kosakovsky Pond and Frost 2005): In all cases d*N*/d*S* was much lower than 1 (table 1), indicating purifying selection as the major driving force in shaping albuminoid gene diversity. Nevertheless, diversifying selection may act upon specific sites or domains; we tested this possibility by applying the LRTs implemented in the *codeml* program (Yang 1997, 2007). LRTs compare models of gene evolution that allow (NSsite models M2a and M8, positive selection models) or disallow (NSsite models M1a and M7, null models) a class of codons to evolve with d*N*/d*S* > 1. These analyses were performed for the *GC* alignment, and independently for the subregions of *ALB, AFP* and *AFM*, split according to the location of recombination breakpoints. As reported in table 2, only for *ALB* both null models were rejected in favor of the positive selection models (after Bonferroni correction for two tests, to account for alignment splitting). These results were confirmed using different models of codon frequency (table 2). In order to identify specific sites subject to positive selection, we applied the BEB analysis (Anisimova et al. 2002; Yang et al. 2005) and the MEME (Murrell et al. 2012). To limit false positives, only sites detected using both methods were considered as positive selection targets. A total of nine positively selected sites were identified for ALB.

**Table 1:** Recombination Breakpoints and Average d*N*/d*S* for Albuminoid Genes

Gene Symbol	Gene Name	Alias	Number of Species	Recombination Breakpoints (Position^a^)	Average d*N*/d*S* (Confidence Intervals)	Human CDS Length (bp)^b^	Alignment Length (bp)	Identity to the Most Distant Ortholog, %
*GC*	Vitamin D-binding protein	—	55	0	0.294 (0.283, 0.306)	1,422	1,422	60.8 (platypus)
*ALB*	Serum albumin	—	54	1 (1538)	0.405 (0.392, 0.418)	1,827	1,827	44.0 (platypus)
*AFP*	Alpha-fetoprotein	HPAFP	50	1 (1443)	0.335 (0.323, 0.348)	1,827	1,827	73.3 (opossum)
*AFM*	Afamin	ALB2, ALBA	49	2 (713,1363)	0.496 (0.481, 0.513)	1,797	1,794	58.9 (platypus)

^a^Positions referred to human sequence.

^b^Coding sequences (CDS) length excluding the STOP codon.

**Table 2:** LRT Statistics for Models of Variable Selective Pressure among Sites (F3 × 4 and F61 Models of Codon Frequency)

Gene	Model	−2Δln *L*	*P* Value (Corrected *P* Value)	Percentage of Sites (Average d*N*/d*S*)	MEME-BEB Sites
*ALB, region 1*					
	F3 × 4				
	M1a versus M2a	90.65	2.07 × 10^−20 ^(4.14 × 10^−20^)	3.99% (2.31)	T107, Q128, E156, E208, A215, A315, T376
	M7 versus M8	92.20	9.54 × 10^−21 ^(1.91 × 10^−20^)	5.74% (1.68)	
	F61				
	M1a versus M2a	106.38	7.92 × 10^−24 ^(1.58 × 10^−23^)	4.11% (2.35)	
	M7 versus M8	94.28	3.37 × 10^−21 ^(6.74 × 10^−21^)	4.81% (1.81)	
*ALB, region 2*					
	F3 × 4				
	M1a versus M2a	30.78	2.07 × 10^−7^ (4.14 × 10^−7^)	6.77% (2.29)	A601, A602
	M7 versus M8	25.47	2.95 × 10^−6^ (5.90 × 10^−6^)	7.07% (1.90)	
	F61				
	M1a versus M2a	19.52	5.77 × 10^−5^ (1.15 × 10^−4^)	5.96% (2.11)	
	M7 versus M8	13.39	1.24 × 10^−3^ (2.47 × 10^−3^)	6.03% (1.68)	

Note.—M1a is a nearly neutral model that assumes one ω class between 0 and 1 and one class with ω = 1; M2a (positive selection model) is the same as M1a plus an extra class of ω > 1. M7 is a null model assuming that 0 < ω < 1 is beta distributed among sites; M8 (positive selection model) is the same as M7 and includes an extra category of sites with ω > 1. 2Δln *L* is twice the difference of the natural logs of the maximum likelihood of the models being compared; *P* value is the *P* value of rejecting the neutral models in favor of the positive selection model; percentage of sites (average d*N*/d*S*) is the estimated percentage of sites evolving under positive selection by M2a and M8 (d*N*/d*S* for these codons).

**Fig. 1.—:**
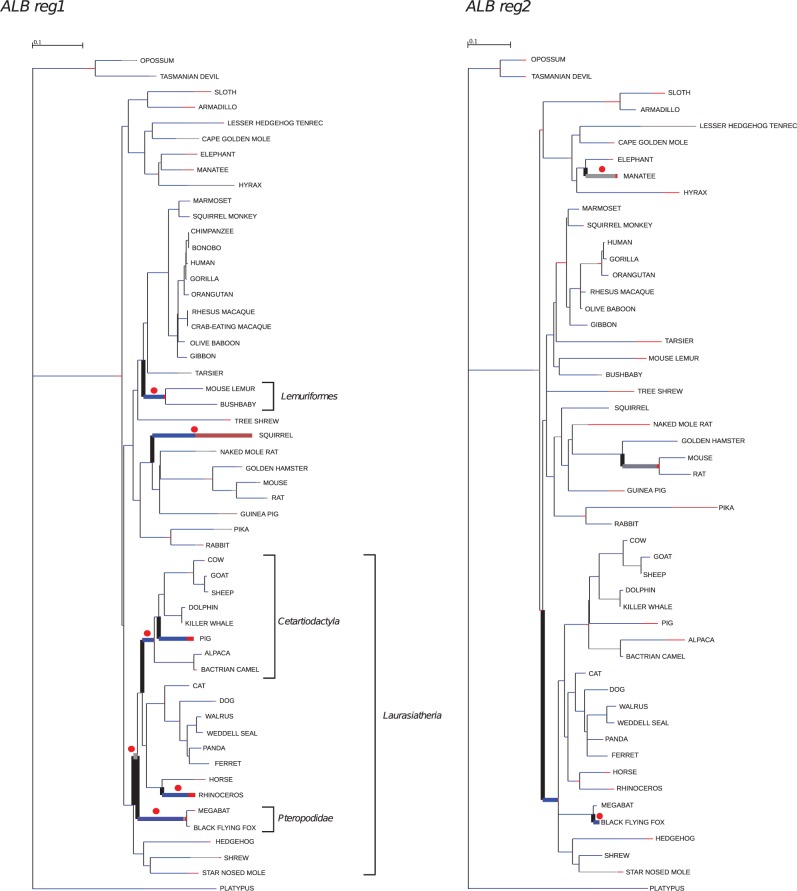
Branch-site analysis of positive selection. Branch lengths are scaled to the expected number of substitutions per nucleotide, and branch colors indicate the strength of selection (ω). Red, positive selection (ω > 5); blue, purifying selection (ω = 0); gray, neutral evolution (ω = 1). The proportion of each color represents the fraction of the sequence undergoing the corresponding positive class of selection. Thick branches indicate statistical support for evolution under episodic diversifying selection as determined by BS-REL. Red dots denote branches that were also detected to be under selection using the PAML branch-site models.

We also explored possible variations in selective pressure among lineages. To this aim, we tested whether models that allow d*N*/d*S* to vary along branches had significant better fit to the ALB data than models that assume one same d*N*/d*S* across the entire phylogeny (Yang and Nielsen 1998). Because this hypothesis was verified, we used the BS-REL method (Kosakovsky Pond et al. 2011) to analyze selection along specific lineages. Branches identified by BS-REL were cross-validated using *codeml* (branch-site LRT models) (Zhang et al. 2005) with FDR correction, as suggested (Anisimova and Yang 2007). Sites positively selected along specific branches were identified through BEB analysis (Zhang et al. 2005) (table 3). This method is sensitive but has low power, thus BEB may fail to identify branch-specific sites even when the LRT is significant (Zhang et al. 2005). Because MEME was specifically developed to detect episodic positive selection, only sites identified by both MEME and BEB were considered (table 3). Several lineages showed statistically supported evidence of positive selection (fig. 1), and positively selected residues were identified for the Lemuriformes (residues 316 and 513), squirrel (residue 142), and manatee (residue 588) branches (table 3).

**Table 3:** LRT Statistics for Models of Variable Selective Pressure along Branches and Branch-Site Tests

Gene	Model	−2Δln *L*	Degrees of Freedom	*P* Value (Bonferroni Corrected *P* Value)
*ALB*, *region 1*	M0 versus M1	418.74	105	4.71 × 10^−39^ (9.42 × 10^−39^)
*ALB, region 2*	M0 versus M1	194.21	105	2.77 × 10^−7^ (5.54 × 10^−7^)

Single branch analysis					
Gene	Foreground Branch (MA versus MA1)	−2Δln *L*	Degrees of Freedom	*P* value (FDR Corrected *P* Value)	MEME-BEB Sites
*ALB, region 1*					
	Laurasiatheria	5.27	1	2.16 × 10^−2 ^(2.45 × 10^−2^)	—
	Cetartiodactyla	5.06	1	2.45 × 10^−2 ^(2.45 × 10^−2^)	—
	Pteropodidae	5.88	1	1.53 × 10^−2 ^(2.29 × 10^−2^)	—
	Lemuriformes	14.66	1	1.29 × 10^−4 ^(7.74 × 10^−4^)	E316, S513
	Squirrel	5.96	1	1.46 × 10^−2 ^(1.75 × 10^−2^)	P142
	Rhinoceros	9.74	1	1.80 × 10^−3 ^(2.94 × 10^−3^)	—
*ALB, region 2*					
	Manatee	12.19	1	4.81 × 10^−4 ^(1.44 × 10^−3^)	K588
	Black flying fox	9.59	1	1.96 × 10^−3^ (2.94 × 10^−3^)	—

Note.—M0 and M1 are free-ratio models that assume all branches to have the same ω (M0) or allow each branch to have its own ω (M1). MA and MA1 are branch-site models that assume four classes of sites: The MA model allows a proportion of codons to have ω ≥ 1 on the foreground branches, whereas the MA1 model does not. 2Δln *L* is twice the difference of the natural logs of the maximum likelihood of the models being compared.

### Most Positively Selected Sites Are Located within Albumin-Binding Sites

**Fig. 2.—:**
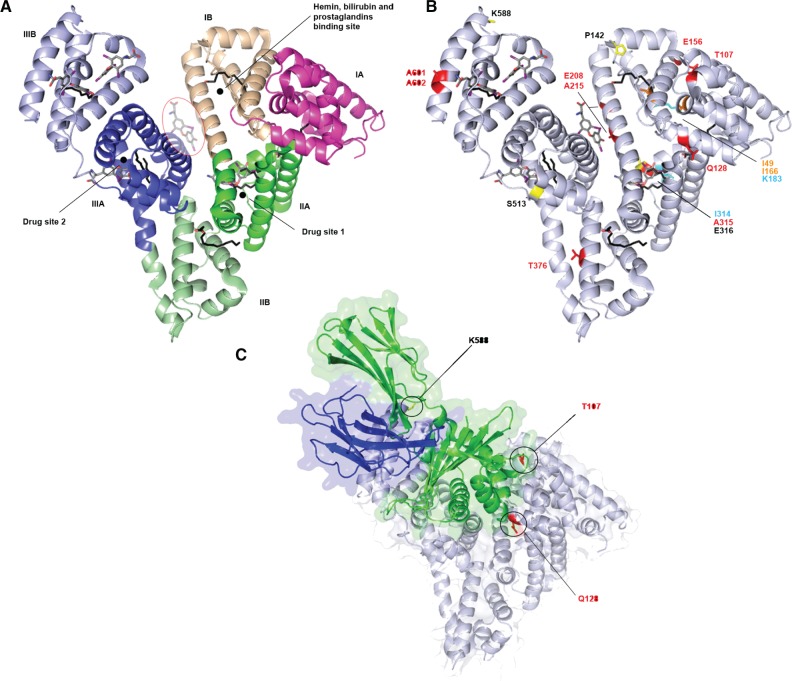
Analysis of positively selected sites in ALB. (*A*) Ribbon representation of human albumin structure (PDB code: 1HK4); color codes denote distinct domains: I (magenta), II (green), and III (blue); dark and light color shades indicate the A and B subdomain organization. Albumin-bound fatty acid (black) and thyroxine molecules (gray) are represented as sticks. The fifth additional fatty acids-induced thyroxine-binding site is circled. Binding sites for heme, bilirubin, prostaglandins, and drugs are mapped onto the structure. (*B*) Positively selected sites mapped onto the albumin structure. Color codes are as follows: Red, positively selected sites in the whole phylogeny; yellow, lineage-specific sites; orange, positively selected sites in the chimpanzee lineage; cyan, positively selected sites in the human lineage. Bound fatty acid (black) and thyroxine molecules (gray) are also shown. (*C*) Representation of the albumin/FcRn complex (PDB code: 4K71). Albumin is shown in gray, the MHC class I-*α* chain in green and *β*2-microglobulin in blue. Positively selected sites located at the contact interface are indicated.

Albumin presents a modular structural organization composed of three homologous helical domains (I, II, and III) arranged in a heart-shaped molecule. Each domain comprises two separated subdomains (A and B), containing six and four helices, respectively. Available crystallographic data revealed binding sites for fatty acids (Bhattacharya et al. 2000), hemin, bilirubin (Zunszain et al. 2003, 2008), thyroxine hormone (Petitpas et al. 2003), prostaglandins (Yamaguchi et al. 2010), and a wide variety of chemical drugs (Curry 2009) (fig. 2*A*).

We mapped the positively selected sites onto the available albumin 3D structures to gain further information about their physiological role.

Interestingly, most sites positively selected in the whole phylogeny are located in close proximity of albumin ligand-binding sites. In particular, A601 and A602 localize in IIIB subdomain, in a pocket responsible for fatty acids and thyroxine binding. T376 lies in proximity of fatty acid-binding site in the IIB subdomain. A315 is located in the subdomain IIA, in the so-called “Drug site 1,” responsible not only for the interaction with a wide range of pharmaceutical compounds but also for recruitment of fatty acids and thyroxine. E208 and A215 localize at the interface between I and III helical domains, where Petitpas et al. (2003) described an ancillary site for thyroxine binding dependent on fatty acid-induced conformational change (fig. 2*B*). Moreover T107 and Q128, as well as K588, a positively selected site identified for the manatee lineage, are close to the contact surface with neonatal Fc receptor (FcRn) (Schmidt et al. 2013) (fig. 2*C*).

Interestingly, positively selected residues for the Lemuriformes and squirrel lineages are in spatial proximity to albumin-binding sites: Residue 142 is located in the fatty acid-binding site of IB subdomain, residue 513 localizes in IIIA subdomain, in the pocket responsible of fatty acid and thyroxine binding, whereas residue 316 lies in “Drug site 1” (fig. 2*B* and table 3).

### Negative and Positive Selection at Genes in the Human and Chimpanzee Lineages

To study the evolution of albuminoids in the human and chimpanzee lineage, we applied a population genetics–phylogenetics approach; specifically, we used gammaMap (Wilson et al. 2011), which integrates intraspecific variation and interspecific diversity to estimate selection coefficients (γ) along coding regions. We exploited data from the 1000 G Pilot Project for CEU, YRI, and CHBJPT (1000 Genomes Project Consortium et al. 2010). For chimpanzees, we used phased SNPs information of ten *Pan troglodytes verus* (Auton et al. 2012).

**Fig. 3.—:**
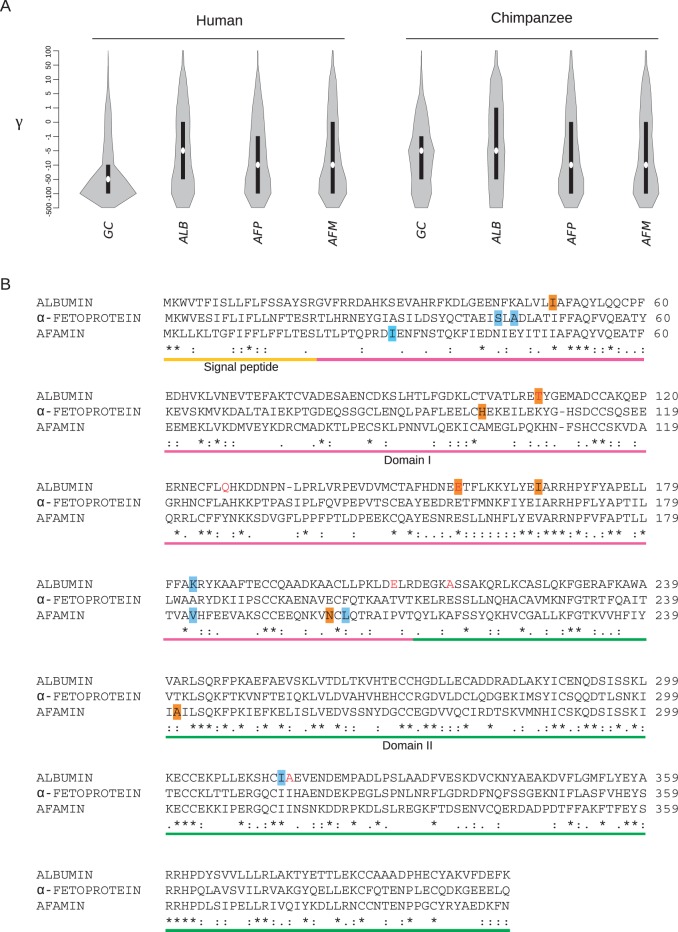
Analysis of selective pressure in the human and chimpanzee lineages. (*A*) Violin plot of selection coefficients (median, white dot; interquartile range, black bar). Selection coefficients (γ) are classified as strongly beneficial (100, 50), moderately beneficial (10, 5), weakly beneficial (1), neutral (0), weakly deleterious (−1), moderately deleterious (−5, −10), strongly deleterious (−50, −100), and inviable (−500). (*B*) Multiple alignment of human ALB, AFM, and AFP. Positively selected sites in the human (cyan) and chimpanzee (orange) lineages are highlighted. Sites selected in whole phylogeny are reported in red. Protein domains are indicated below the alignment: Signal peptide in yellow, domain I in magenta, and domain II in green.

In both humans and chimpanzees, we observed a general preponderance of codons evolving under negative selection (γ < 0) in all genes. The most striking difference was observed for *GC*, which showed stronger purifying selection compared with the other three genes in humans but not in chimpanzees (fig. 3*A*).

We next used gammaMap to identify specific codons evolving under positive selection in humans and chimpanzees. To be conservative, we declared a codon to be targeted by positive selection when the cumulative posterior probability of γ ≥ 1 was > 0.75, as suggested (Quach et al. 2013).

No positively selected codon was identified for *GC*. In *ALB* gene, two and four selected sites were detected in humans and in chimpanzees, respectively (supplementary table S2, Supplementary Material online)*.* Two of the *P. troglodytes* selected sites (T107 and E156) correspond to residues targeted by positive selection in the whole phylogeny (table 2 and supplementary table S2, Supplementary Material online). The location of positively selected sites in humans and chimpanzees relative to the albumin 3D structures is shown in figure 2*B.*

Moreover, positively selected codons were also identified for *AFP* and *AFM* (supplementary table S2, Supplementary Material online). Unfortunately, no 3D structure is available for alpha-fetoprotein and afamin; we thus performed a multiple alignment of the protein sequences of AFP, ALB, and AFM. Interestingly, corresponding sites at position 183 are positively selected in *ALB* and *AFM* in humans. In albumin, this residue lies in the surrounding of the prostaglanding-binding site, as is the case for I166, positively selected in the human lineage (figs. 2 and 3*B*). The *AFP* codons 42 and 44*,* which are positively selected in humans, are in proximity to *ALB* codon site 49, positively selected in *P. troglodytes.* Furthermore, the *AFP* codons 200 and 202*,* which are positively selected in chimpanzees and humans, respectively, are in proximity to *ALB* codon site 208 (selected in the whole phylogeny), located in the additional fatty acid-induced thyroxine-binding site (figs. 2 and 3*B*). Finally, the positively selected site at position 100 of *AFP* is close to the positively selected codon site 107 in *ALB,* which localizes at the albumin/FcRn interface (Schmidt et al. 2013) (figs. 2 and 3*B*).

### *GC* Is a Positive Selection Target in Human Populations

We finally investigated the action of natural selection for albuminoid genes during the recent evolutionary history of human populations. To this aim, we used the 1000 G Pilot data to calculate nucleotide diversity (measured as π [Nei and Li 1979] and θ_W_ [Watterson 1975]), and Tajima’s *D* (Tajima 1989) over whole gene regions. For all single nucleotide variants mapping to albuminoid genes and in their 60-kb flanks (30 kb up- and downstream), we calculated pairwise *F*_ST_ (Wright 1950), an estimate of population genetic differentiation, and we performed the DIND test (Barreiro et al. 2009); DIND compares the intra-allelic diversity associated with the ancestral and derived alleles (iπ_A_/iπ_D_), is well suited for low-coverage data, and has good power in most DAF ranges (Barreiro et al. 2009; Fagny et al. 2014). To assess statistical significance (in terms of percentile rank), we obtained empirical distributions for all the parameters and tests from a randomly selected set of 3,000 human genes (see Materials and Methods)*.* We considered genes as positive selection targets if significant results were obtained for the same population in at least two statistics based on different features (e.g., DIND and *F*_ST_, both with a rank >0.95). We also calculated normalized values for DH (Zeng et al. 2006) in sliding windows along the analyzed genomic regions; DH was used as a confirmatory signature rather than an a priori evidence, because of the statistical problems inherent to sliding-window analyses.

Although whole gene analyses of nucleotide diversity and Tajima’s D did not show evidence of selection at any of the albuminoid genes (supplementary table S3, Supplementary Material online), the single variant approaches (i.e., DIND and *F*_ST_) detected selection signatures at *GC*. In fact, the gene showed signals of positive selection in the three populations, with different events involving distinct variants.

**Fig. 4.—:**
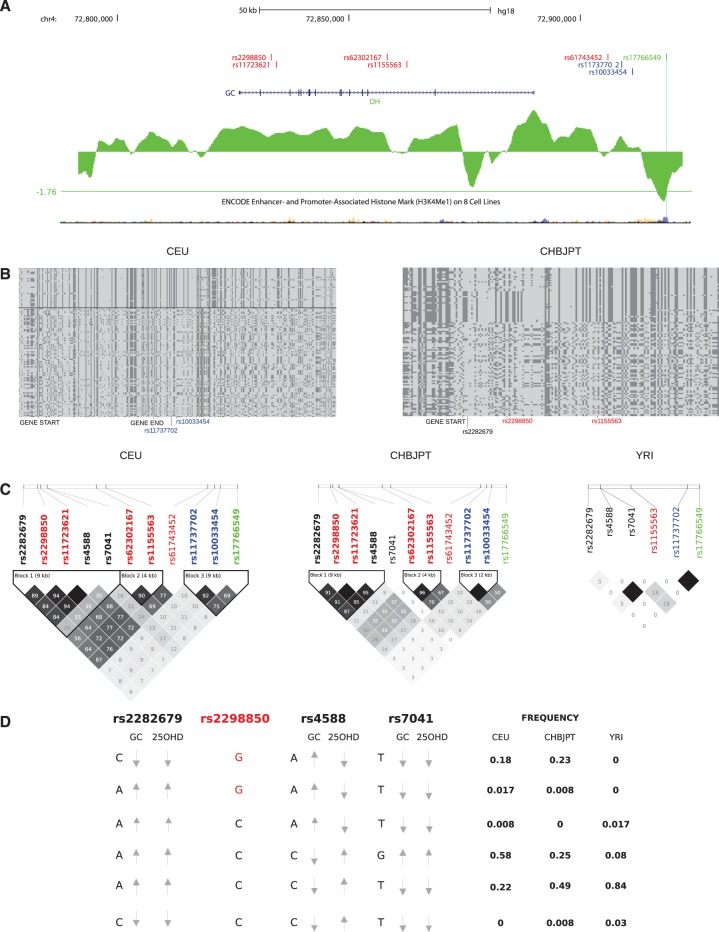
Natural selection at GC in human populations. (*A*) Schematic representation of *GC* within the UCSC Genome Browser view. The location of the selection targets and of the GWAS variant are also shown, together with relevant ENCODE tracks. Variants are color coded by population: YRI, green; CEU, blue; CHBJPT, red. A sliding-window analysis of normalized DH is also shown for YRI; the horizontal line represents the fifth percentile in the distribution of DH values. (*B*) Schematic representation of CEU (left) and CHBJPT (right) haplotypes for genomic regions centered around the selected variants. Each line represents a haplotype, columns indicate polymorphic positions. Dark gray, derived alleles; light gray, ancestral alleles. The thick horizontal line separates haplotypes carrying the ancestral (bottom) and derived (top) allele. (*C*) LD plot (r2) for the selected targets in the three populations, the GWAS variant, and two nonsynonymous SNPs in *GC* (see text). Variants are color coded as in (*A*). (*D*) Schematic representation of the major human haplotypes for rs2283679, rs2298850, rs4588, and rs7041. Arrows indicate the association with increased or decreased levels of vitamin D-binding protein (GC) and 25-hydroxyvitamin D (25ODH). The selected allele in CHBJPT for rs2298850 is colored in red. The frequency for each haplotype in the three populations is also reported.

In YRI one variant (rs17766549) at high DAF (0.98) was an *F*_ST_ outlier in the YRI/CEU comparison, and also displayed an unusually high DIND value (table 4). These results were confirmed by the DH analysis (fig. 4*A*): The variant fell in a local valley of DH, in line with this statistic having maximum power for high-frequency sweeps (Zeng et al. 2006) (table 4). Overall, these results indicate that a selective sweep drove the frequency increase of this variant in YRI. The SNP is located in a region where enhancer- and promoter-associated histone marks are located, as assessed by ENCODE data (fig. 4*A*).

**Table 4:** DIND and *F*_ST_ Results for the Best Candidate Selected Variants

SNP ID	DAF			DIND Rank (Pop^a^)	*F*_ST_ Rank (Comparison)
	YRI	CEU	CHBJPT		
rs17766549	0.98	0.77	0.85	0.96 (YRI)	0.95 (YRI/CEU)
rs11737702	0.02	0.27	0.16	0.99 (CEU)	0.96 (YRI/CEU)
rs10033454	0	0.28	0.16	0.97 (CEU)	>0.99 (YRI/CEU)
rs2298850	0	0.19	0.24	0.96 (CHBJPT)	>0.99 (YRI/CHBJPT)
rs11723621	0	0.20	0.24	0.99 (CHBJPT)	>0.99 (YRI/CHBJPT)
rs62302167	0	0.22	0.36	0.96 (CHBJPT)	>0.99 (YRI/CHBJPT)
rs1155563	0.02	0.22	0.37	0.98 (CHBJPT)	0.95 (YRI/CHBJPT)
rs61743452	0	0.22	0.37	0.97 (CHBJPT)	>0.99 (YRI/CHBJPT)

^a^Population showing signatures of selection.

In CEU signals of positive selection were detected for two variants (rs11737702 and rs10033454) in strong linkage disequilibrium (LD) (*r*^2^ = 0.92) (fig. 4*C*), and representing *F*_ST_ and DIND outliers (table 4). The two variants are located upstream the transcription start site of *GC* and define a homozygous haplotype extended along the gene region (fig. 4*B*).

In CHBJPT several variants were found to be outliers in the *F*_ST_ and DIND analyses, two of them are in full LD (rs2298850, rs11723621, *r*^2^ = 1) (fig. 4 and table 4). Interestingly, they also show strong LD with another variant (rs2282679, *r*^2^ = 0.91) that has been associated with circulating vitamin D levels in genome-wide association studies (GWASs) (Ahn et al. 2010; Wang et al. 2010; Lasky-Su et al. 2012) (fig. 4*C*). Three other variants (rs62302167, rs1155563, and rs61743452) showed high values of *F*_ST_ and DIND, but not LD with the previous ones (*r*^2^ = 0.302) (table 4 and fig. 4*C*); all these results together support that a selective sweep occurred in Asian populations; the selective event was likely disrupted by recombination, leaving two distinct signatures (fig. 4*B*).

We next investigated the relationship between the selected variants in the three populations and two known polymorphisms that define three different variant vitamin D-binding proteins, named GC*1S, GC*1F, and GC2 (supplementary table S4, Supplementary Material online) (Lauridsen et al. 2005). One of these two variants (rs4588) is in tight LD with two of the SNPs selected in Asians (fig. 4*C*); the selected alleles of rs2298850 and rs11723621 are in phase with the A allele of rs4588, which correlates with increased levels of vitamin D-binding protein and decreased levels of 25-hydroxyvitamin D (fig. 4*D*) (Powe et al. 2013). Nevertheless, the positively selected alleles are also in phase with the C allele of rs2282679, which decreases the concentrations of both 25-hydroxyvitamin D and vitamin D-binding protein (Wang et al. 2010) (fig. 4*D*).

## Discussion

Large-scale analyses of positive selection at the inter- and intraspecific levels have described gene functional classes that are commonly targeted by natural selection; these most frequently include immune response, chemosensory perception, reproduction, and, in the case of human populations, pigmentation and diet (Voight et al. 2006; Kosiol et al. 2008; Pickrell et al. 2009; Grossman et al. 2013). Albuminoid genes carry out housekeeping functions and are not directly involved in any of the functions generally regarded as common targets of natural selection, although they may have a role in reproduction (see below). Albumin, the most abundant protein in human serum, has been the subject of intense investigation, also because of its central role in the transport and activation of several pharmacological compounds (Curry 2009). Nonetheless, the evolutionary history of albuminoid genes has mainly been analyzed in terms of gene duplication/divergence events (Nishio et al. 1996; Gibbs et al. 1998). In this respect it is worth mentioning that the four genes were shown to derive from a series of duplications from a common ancestor, the most recent of which originated the *AFM* and *AFP* genes from a common precursor. This event has been dated back to about 250 Ma (Gibbs et al. 1998)—that is, earlier than the separation of therian and prototerian mammals (Warren et al. 2008). Thus, all duplication events occurred before the divergence of the species we analyzed.

Herein, we wished to gain further insight into the evolution of albuminoids by means of inter- and intraspecific comparison of orthologous genes. Evolutionary analysis along the mammalian phylogeny indicated no evidence of adaptive evolution for *GC*, *AFM*,** and *AFP*. Conversely, strong signatures of recurrent positive selection were detected for *ALB*. The analyses we performed relied on relatively conservative approaches, aimed at minimizing false positive results. We thus required two neutral models to be rejected in favor of the positive selection models; likewise, sites and lineages were declared positively selected only if they were detected by two distinct methods. Because of these assumptions we may have missed weak signals and we most likely underestimated the fraction of sites that evolved under episodic positive selection. Ad-hoc analyses using a population genetics–phylogenetics approach were performed in order to detect positively selected sites in the human and chimpanzee lineages.

The combination of these methods detected several selected sites in ALB, and most of these are located in functional regions involved in ligand binding. In particular, we found evidence of positive selection at three consecutive sites (I314, A315, and E316) facing the so-called Drug site 1, in the IIA subdomain. This binding site is involved in the binding not only of hydrophobic, bulky, heterocyclic chemical drugs presenting a centrally located negative charge (e.g., warfarin) but also of fatty acid and thyroxine molecules, revealing adaptability to a wide range of ligands and involvement in different physiological processes. Similarly, E208 and A215 are located in the fatty acid-induced thyroxine-binding site in the IIB subdomain, whereas A601 and A602 are in spatial proximity to the high affinity fatty acid-binding site in the IIIB subdomain, also involved in thyroxine recruitment (Petitpas et al. 2003) (fig. 2).

Thus, these indicate that positive selection preferentially targeted regions implicated in ligand binding, suggesting a selective pressure favoring the modulation of albumin binding capability.

Albumin is also known to act as an endogenous catalyst in metabolism of prostaglandin D2, a potent sleep-promoting substance (Urade and Hayaishi 2011), yielding to the production of Δ12-PGJ2, a metabolite involved in metabolic and immunological processes. Interestingly, I166 and K183, positively selected in the chimpanzee and human lineages, respectively, localize in a D-shaped cavity in the center of the four-helix bundle of subdomain IB responsible for prostaglandin binding. This domain presents a small number of nucleophyle residues, including K183, and shows an extraordinary reactivity; even if it is not considered a true binding site, it is accepted that reactive aromatic electrophile compounds have a marked specificity for this region (Yamaguchi et al. 2010). Intriguingly, we detected a human positively selected site in the afamin sequence at a position corresponding to K183. To date, the physiological functions of afamin are still not fully understood but there are clear evidences that it is a vehicle for vitamin E in body fluids (Voegele et al. 2002). Positive selection at this site suggests a role in ligand recognition and binding modulation, although the range of afamin natural interactors is not fully defined, yet.

The positively selected ALB residue Q128 precedes H129, which was demonstrated by site-directed mutagenesis to be involved in the conformational neutral to base (N-B) transition between domains I and II, observed at pH increasing conditions. The structural N-B transition is a conserved mechanism and is thought to have a relevant role in the transport and cellular uptake mechanisms of many endogenous and exogenous compounds, modulating binding affinity (Yang et al. 2005). In fact, a similar structural change is also observed after binding of warfarin, one of the most widely used anticoagulants (Petersen et al. 2002; Yang et al. 2005; Ha and Bhagavan 2013)*.*

The positively selected Q128 residue also localizes at the FcRn–albumin interface, as is the case for T107 and K588 (selected in the manatee branch) (fig. 2*C*). FcRn competes with natural ligands for the binding into the hydrophobic pockets in domain III, and mediates endosomal salvage from degradation, extending albumin half-life and prolonging its action in the organism (Schmidt et al. 2013). This effect might be relevant in specific physiological conditions. For instance, increased albumin serum concentrations are observed during hibernation, an adaptive physiological response to cold and inhospitable environments. A recent proteomics analysis in hibernating arctic ground squirrels suggested that the high increment of albumin levels is not a passive response to the dehydration that naturally accompanies hibernation, but a finely regulated process, although the comprehension of the underlying molecular machinery remains unclear (Shao et al. 2010).

To date, the evolutionary origin of hibernation is unknown; many species of hibernators are interspersed across the whole phylogeny and often closely related to nonhibernators, opening up two opposite hypotheses that consider the common ancestor as a hibernator and as a nonhibernator. Anyway, the widespread distribution of hibernating behavior suggests that physiological processes regulating this phenotype are analogous among different species (Srere et al. 1992; Villanueva-Canas et al. 2014). Exploring possible variations in selective pressure among lineages, we detected positive selection in many branches including those leading to Laurasiatheria, which include both hibernators (e.g., Pteropodidae, showing episodic selection at ALB) and nonhibernators, and to Cetartiodactyla, all of them nonhibernators.

We also identified positively selected sites in the Lemuriformes, squirrel, and manatee branches. Lemurs exhibit a torpor state during the dry winter months, and in some cases undergo seasonal hibernation, which is unusual for primates, but is an affirmed physiological and behavioral strategy for squirrels. Hibernation is characterized by lower respiratory, heart and metabolic rates, as well as decreased body temperature. During this state, fatty acids stored in the adipose tissue are the main source of energy (Florant and Healy 2012); they are released in the circulation and bound by albumin (Bhattacharya et al. 2000). Interestingly, the positively selected residues localize in close proximity to the ligand-binding pockets of serum albumin, which can allocate not only fatty acids but also thyroxine; this latter is involved in the regulation of all the physiological processes that are shown to be slowed down during hibernation.

As for the manatee branch, a positively selected site localizes in close proximity to a thyroxine-binding site and at the FcRn interaction interface, as previously discussed. Manatees are not hibernators but, among marine mammals, exhibit a very low metabolic rate despite of high body mass; the herbivorous feeding behavior provides a low caloric intake. Intriguingly, during periods of reduced food consumption these mammals activate thyroid hormone-promoted lipolysis to provide for energy (Ortiz et al. 2000).

Overall, these data suggest that the selective pressure acting on albumin is related to the modulation of its extraordinary ligand-binding adaptability in response to variable environment conditions, possibly including adaptation to hibernation/torpor.

Because albumin represents an important carrier of several pharmacological compounds and also acts as a drug activator through its esterase-like activity (Kragh-Hansen 2013), pharmacokinetic experiments in animal models would benefit from taking into account that positive selection at ALB may result in wide variability of binding/activity among mammals, especially at “Drug site 1.”

Albumin and alpha-fetoprotein are the most abundant proteins in human adult and fetal serum, respectively. Curiously, subjects lacking ALB and ALF due to genetic defects develop normally and appear to be healthy, suggesting that these two genes, despite encoding extremely abundant proteins, are dispensable (Koot et al. 2004; Toye et al. 2012). Nevertheless, *Afp*^−/−^ female mice are infertile due to a dysfunction of the hypothalamic/pituitary axis leading to anovulation (Gabant et al. 2002; De Mees et al. 2007). Because congenital absence of *AFP* in humans has only been described in children (Sharony et al. 2004; Petit et al. 2009), it remains to be evaluated whether alpha-fetoprotein is also important for fertility in humans. Interestingly, afamin may also be involved in reproductive functions, as it is present at relatively high levels in human follicular fluid (Jerkovic et al. 2005; Angelucci et al. 2006) and its concentration increases in maternal serum during pregnancy (Hubalek et al. 2014). Likewise, growing evidence suggests that although CAA is associated with mild clinical symptoms (including hypercolestreremia, respiratory tract infections, and edema), placental dysfunction, preterm birth, and intrauterine growth retardation are more common in CAA subjects compared with the general population (Koot et al. 2004; Toye et al. 2012). As for GC, total deficiency has never been described in humans, although the serum concentration of the protein (and of 25-hydroxyvitamin D) varies depending on ethnicity (Powe et al. 2013). Mice lacking GC are healthy, but more susceptible to osteopathy when kept on vitamin D-deficient diets (Safadi et al. 1999).

Thus, genetic defects in albuminoid genes, although well-tolerated in the modern environmental setting of human populations (and of laboratory mice), might have represented targets of purifying selection during the evolution of humans and other mammals. This is even more likely if the possible involvement of AFP, AFM, and ALB in reproductive functions is considered. Indeed, analysis of selection coefficients for the four genes in the human and chimpanzee lineages indicated a clear prevalence of codons evolving under negative selection, with *AFM* and *GC* showing the strongest level of constraint in *P. troglodytes* and humans, respectively. In particular, human GC showed a large proportion of codons with very negative selection coefficients (γ < −50). The reasons why this gene evolved under stronger constrains compared with other albuminoid genes and to the chimpanzee counterpart are unclear. Vitamin D-binding protein prolongs the half-life of 25-hydroxyvitamin D and promotes its reabsorption in the kidney (Nykjaer et al. 1999). Therefore, GC functions as a reservoir of 25-hydroxyvitamin D and regulates its bioavailability (Powe et al. 2013). Thus, changes in skin characteristics (hair loss and variable pigmentation), as well as in the level of sun exposure due to lifestyle and migration to nonequatorial latitudes, might have resulted in stronger selective pressure on GC in humans. Indeed, light skin pigmentation in humans is thought to have evolved in response to decreased ultraviolet (UV) irradiation to ensure sufficient 25-hydroxyvitamin D biosynthesis (Chaplin and Jablonski 2009). Deficiency of vitamin D is associated with childhood rickets, osteomalacia, and fractures, in addition to several other conditions unrelated to bone metabolism. It is therefore conceivable that genes other than those involved in skin pigmentation, but related to 25-hydroxyvitamin D metabolism, have been targeted by natural selection. In line with this view, Ramagopalan et al. (2010) detected a significant enrichment of vitamin D receptor binding sites within regions of positive selection in Asian and European populations, but not in Africans.

Recent evidence indicated that black Americans have lower levels of GC and 25-hydroxyvitamin D than whites, but similar concentrations of bioavailable 25-hydroxyvitamin D (Powe et al. 2013), suggesting that vitamin D-binding protein acts to maintain the homeostatic control of 25-hydroxyvitamin D bioavailability. Ethnic differences were found to be largely explained by genetic effects, with two nonsynonymous variants (rs7041 and rs4588) account for a large portion of the variability in GC concentrations (Powe et al. 2013). The two SNPs also explain a much lower proportion of variability in 25-hydroxyvitamin D levels, and rs4588 showed an opposite effect on GC and 25-hydroxyvitamin D concentrations. Among the selected variants we detected in Asian populations, rs2298850 and rs11723621 are in strong LD with rs4588 and the selected alleles are in phase with the allele (rs4588-A) that associates with increased and decreased levels of vitamin D-binding protein and 25-hydroxyvitamin D, respectively. Nonetheless, the selected Asian variants are also in LD with rs2282679, identified in GWASs as the strongest association signal for 25-hydroxyvitamin D levels in Europeans, and also for GC concentrations (Ahn et al. 2010; Wang et al. 2010); in this case, though, the selected alleles are in phase with rs2282679-C, which decreases the concentrations of both 25-hydroxyvitamin D and its carrier. Indeed, haplotype analysis revealed that in European and Asian populations major *GC* haplotypes carry alleles with reported opposite effects at rs4588 and rs2282679. These data indicate that either association studies had some technical flaws or multiple *GC* variants in full or partial LD modulate vitamin D-related traits with opposite allelic effects. Clearly, future studies would greatly benefit from taking haplotype information into account. Moreover, we detected additional and independent selection signatures in CEU and YRI; these are located downstream the transcription end site of the gene and rs17766549 maps to a region where histone marks associated with regulatory elements have been described; analysis using HaploReg (Ward and Kellis 2012) also indicated that the variant affects a binding site for the sterol regulatory element binding protein, an important hepatic transcription factor.

Overall, these data indicate a complex selective scenario for *GC*, which likely results from its important role in vitamin D homeostasis and may also partially be affected by local environmental conditions (e.g., UV irradiation) and skin pigmentation. Indeed, the selected alleles in European and Asians are virtually absent in African populations, suggesting that they may represent an adaptation to life at nonequatorial latitudes.

## Supplementary Material

Supplementary fig. S1 and tables S1–S4 are available at *Genome Biology and Evolution* online (http://www.gbe.oxfordjournals.org/).

Supplementary Data
