# Supplementary material for: Albuminoid Genes: Evolving at the Interface of Dispensability and Selection
Source: Genome Biol Evol. 2014 Oct 27;6(11):2983–97. doi: 10.1093/gbe/evu235 (PMC4255767; doi:10.1093/gbe/evu235)
Supplement: Supplementary Data [file supp_6_11_2983__index.html]

Albuminoid genes: evolving at the interface of dispensability and selection — Albuminoid Genes: Evolving at the Interface of Dispensability and Selection — Supplementary Data 

# Albuminoid Genes: Evolving at the Interface of Dispensability and Selection

## Supplementary Data

files

**Files in this Data Supplement:**

- Supplementary Data - pdf file
