# Supplementary material for: Albuminoid Genes: Evolving at the Interface of Dispensability and Selection
Source: Genome Biol Evol. 2014 Oct 27;6(11):2983–97. doi: 10.1093/gbe/evu235 (PMC4255767; doi:10.1093/gbe/evu235)
Supplement: Supplementary Data [file supp_evu235_Supplementary.pdf]

Figure S1. Multiple sequence alignments

ALBUMIN

Human	MKWVTFISLLFLFSSAYSRGVFRRDAHKSEVAHRFKDLGEENFKALVLIAFAQYLQQCPF
Chimpanzee	MKWVTFISLLFLFSSAYSRGVFRRDAHKSEVAHRFKDLGEENFKALVLVAFAQYLQQCPF
Bonobo	MKWVTFISLLFLFSSAYSRGVFRRDAHKSEVAHRFKDLGEENFKALVLVAFAQYLQQCPF
Gorilla	MKWVTFISLLFLFSSAYSRGVFRRDAHKSEVAHRFKDLGEETFKALVLVAFAQYLQQCPF
Orangutan	MKWVTFISLLFLFSSAYSRGVFRRDAHKSEVAHRFKDLGEEKFKALVLIAFAQYLQQCPF
Gibbon	MKWVTFISLLLLFSSAYSRGVFRRDAHKSEIAHRFKDLGEENFKALVLVAFAQYLQQCPF
Rhesus	MKWVTFISLLFLFSSAYSRGVFRRDTHKSEVAHRFKDLGEEHFKGLVLVAFSQYLQQCPF
Crab-eating macaque	MKWVTFISLLFLFSSAYSRGVFRRDTHKSEVAHRFKDLGEEHFKGLVLVAFSQYLQQCPF
Olive baboon	MKWVTFISLLFLFSSAYSRGVFRRDAHKSEVAHRFKDLGEEHFKGL--------------
Marmoset	MKWVTFISLLFLFSSAYSRGVFRRDAHKSEVAHRFKDLGEEHFKDLVLVPFSQYLQQCPF
Squirrel monkey	MKWVTFISLLFLFSSAYSRGVFRRDAHKSEVAHRFKDLGEENFKALVLVAFSQYLQQCPF
Tarsier	MKWVTFISLLFLFSSAHSRGLFRRDAHKSEIAHRYKDLGETDFKYLVLITFSQYLQKCPF
Mouse lemur	MKWVTFISLLFLFSSAYSRGVFRRDTHKSELAHRFNDLGEGNFKALVLVTFSQFLQKCPF
Bushbaby	MKWVTFISLLFLFSSAYSRGVFRRDTHKSELAHRYNDLGEEHFKALVLVTFSQFLQKCPF
Chinese hamster	MKWVTFISLLFLFSSAYSRGVVRRETHKSEIAHRFKDLGEENFKGLVLIAFSQYLQQCPF
Squirrel	MKWVTFIFLLFLFSSAYSRGVFRREAGKSEIIKRFREFGEQEFKGLVLVTFSQILQKTSY
Golden hamster	MKWVTFLLLLFVSDSAFSRGLFRRDAHKSEIAHRFKDLGEQHFKGLVLIAFSQFLQKCPY
Mouse	MKWVTFLLLLFVSGSAFSRGVFRREAHKSEIAHRYNDLGEQHFKGLVLIAFSQYLQKCSY
Rat	MKWVTFLLLLFISGSAFSRGVFRREAHKSEIAHRFKDLGEQHFKGLVLIAFSQYLQKCPY
Naked mole rat	MKWVTFISLLLLFSSAYSRGVFRREAHKSEIAHRFTDLGEKHFKGLALITFSQYLQKCSF
Guinea pig	MKWVTFISLLFLFSSVYSRGVFRREAHKSEIAHRFNDLGEGHFKGLVLITLSQHLQKSPF
Rabbit	MKWVTFISLLFLFSSAYSRGVFRREAHKSEIAHRFNDVGEEHFIGLVLITFSQYLQKCPY
Pika	MKWVTFISLLFLVSSAYSRGLIRREAHKSEIAHRFKDVGEENFKGLVLIAFSQYLQKCPF
Pig	MKWVTFISLLFLFSSAYSRGVFRRDTYKSEIAHRFKDLGEQYFKGLVLIAFSQHLQQCPY
Alpaca	MKWVTFISLLFLFSSVYSRGVFRRDTYKSEIAHRFKDLGEDDFKGLVLIAFSQYLQQCPF
Bactrian camel	MKWVTFISLLFLFSSVYSRGVFRRDTHKSEIAHRFKDLGEDDFKGLVLIAFSQYLQQCPF
Dolphin	MKWVTFISLIFLFSSAYSRGVFRRDTHKSEIAHRFNDLGEENFKGLVLIAFSQYLQQSPF
Killer whale	MKWVTFISLIFLFSSAYSRGVFRRDTHKSEIAHRFNDLGEENFKGLVLIAFSQYLQQSPF
Cow	MKWVTFISLLLLFSSAYSRGVFRRDTHKSEIAHRFKDLGEEHFKGLVLIAFSQYLQQCPF
Sheep	MKWVTFISLLLLFSSAYSRGVFRRDTHKSEIAHRFNDLGEENFQGLVLIAFSQYLQQCPF
Goat	MKWVTFISLLLLFSSAYSRGVFRRDTHKSEIAHRFNDLGEENFQGLVLIAFSQYLQQCPF
Horse	MQWVTFVSLLFLFSSAYSRGVLRRDTHKSEIAHRFNDLGEKHFKGLVLVAFSQYLQQCPF
White rhinoceros	MKWVTFISLLFLFSSAYSRGVFRRHSYKSEIAHRFNDLGEPHFKGLVLVAFSQYLQQGTY
Cat	MKWVTFISLLLLFSSAYSRGVTRREAHQSEIAHRFNDLGEEHFRGLVLVAFSQYLQQCPF
Dog	MKWVTFISLFFLFSSAYSRGLVRREAYKSEIAHRYNDLGEEHFRGLVLVAFSQYLQQCPF
Ferret	MKWVTFISLLFLFSSAYSRGVTRREAQQSEIAHRYNDLGEEHFKGLVLVAFSQYLQQCPF
Panda	MKWVTFISLLFLFSSAYSRGVTRREAQQSEIAHRYNDLGEEHFRGLVLVAFSQYLQQCPF
Pacific walrus	MKWVTFISLLFLFSSAYSRGVVRRETQQSEIAHRYNDLGEEHFRGLVLVAFSQYLQQSPF
Weddell seal	MKWVTFISLLFLFSSAYSRGVMRREAQQSEVAHRYNDLGEEHFRGLVLVAFSQYLQQCPF
Black flying fox	MKWVTFISLLFLFSSAYSRGVFRRDTHKSEIAHRYNDLGEEHFRELVLITFSQYLQQCPF
Megabat	MKWVTFISLLFLFSSAYSRGVFRRDTHKSEIAHRYNDLGEEQFRELVLITFSQYLQQCPF
Hedgehog	MKWVTFLSLLFLISSAYSRGVIRRDAHKSEIAHRFSDLGEQNFKALVLITFSQFLQKCPY
Shrew	MKWATFISLLFLFNSAYSRGVFRRDAHKSEIAHRFKDLGEENFKGLVLIAFSQYLQKCPF
Star nosed mole	MKWVTFISLLFLFSSAYSRGVFRRDAHKSEIAHRFNDLGEEAFKGLVLVAFSQYLQKGPF
Hyrax	MKWITFIALLFLFSSASSRGLFRRDAHKSEIAHRYNDLGENIFKGL-LISLAQHL-----
Elephant	MKWVIFISLSFLFSSAYSRGVFRREAYKSEIAHRYKDLGEDLFKGLLLISFAQYLQKSPY
Manatee	MKWVTFISLLFLFSSAYSKGVFRRDTHKSEIAHRYNDLGEDLFTGLLLISFSQYLQKSPF
Cape golden mole	MKWVTFISLLFLFSSAYSRGVFRRDAHKSELAHRYTDLGEDVFKGLLLIAFSQYLQKCPY
Tenrec	MKWVTFISLLFLFSSASSRGLFRRDAHKSEIAHRYKDLGEEFFKGLLLITFSQYLQKCPY
Armadillo	MKWVIFISLLFLFSSAYSKGVFRRDAHKSEIAHRFNDLGKENFRLLTLVMFAQYLQKCPF
Sloth	MKWVTFISL-FLFSSAYSRGMVRREADKSEIAHRFKDLGEENFRG---------------
Opossum	MKWVTFISLIFLFSSVYSRDLFRRDAPKSEIAKRYRDLGEENVKALVLITFAQYLQKCPF
Tasmanian devil	MKWVTFISLIFLFSSVHSREIFRRDAPTSEIAKRYRDLGEENVKGLVLITFAQYLQKCPF
Platypus	M---

Human	EDHVKLVNEVTEFAKTCVADESAENCDKSLHTLFGDKLCTVATLRETYGEMADCCAKQEP
Chimpanzee	EDHVKLVNEVTEFAKTCVADESAENCDKSLHTLFGDKLCTVATLREKYGEMADCCAKQEP
Bonobo	EDHVKLVNEVTEFAKTCVADESAENCDKSLHTLFGDKLCTVATLREKYGEMADCCAKQEP
Gorilla	EDHVKLVNEVTEFAKTCVADESAENCDKSLHTLFGDKLCTVATLRETYGEMADCCAKQEP
Orangutan	EDHVKLVNEVTEFAKTCVADESAENCDKSLHTLFGDKLCTVATLRETYGEMADCCAKQEP

```
Gibbon                EDHVKLVNEVTEFAKTCVADESAENCDKSLHTLFGDKLCTVATLRETYGEMADCCAKQEP
Rhesus                EEHVKLVNEVTEFAKTCVADESAENCDKSLHTLFGDKLCTVATLRETYGEMADCCAKQEP
Crab-eating macaque   EEHVKLVNEVTEFAKTCVADESAENCDKSLHTLFGDKLCTVATLRETYGEMADCCAKQEP
Olive baboon          ----------------------------------------VATLRETYGEMADCCAKQEP
Marmoset              EEHLKLVNEVTEFAKTCVADETAENCDKSLHTLFGDKLCTVATLRETYGDMADCCAKQEP
Squirrel monkey       DDHVKLVNEVTEFAKTCVADESAENCDKSLHTLFGDKLCTVATLRETYGDMADCCAKQEP
Tarsier               QEHVKLMNEVIEFAKTCVADESAENCDKSLHTLFGDKLCTVASLREVYGEMADCCAKQEP
Mouse lemur           EDHVKLVNEITDFAKTCVADESAENCDKSLHTLLGDKLCTVASLREKYGEMADCCAKQEP
Bushbaby              EDHVKLVNEVTEFAKTCAADESAENCDKSLHTLLGDKLCTVASLREKYGEMADCCAKQEP
Chinese hamster       EEHVKLVGEVTEFAKTCVADETAENCDKSLHTLFGDKLCTIATLRETYGDMADCCAKQEP
Squirrel              DDLAKFTTEVTDLAKACVADEFGVDCNKPLDTIFGEEACKIATLRDTYGDMADCCANQDL
Golden hamster        EEHVKLVNEVTDFAKTCVADESAENCDKSLHTLFGDKLCAIPTLRDSYGELADCCAKKEP
Mouse                 DEHAKLVQEVTDFAKTCVADESAANCDKSLHTLFGDKLCAIPNLRENYGELADCCTKQEP
Rat                   EEHIKLVQEVTDFAKTCVADENAENCDKSIHTLFGDKLCAIPKLRDNYGELADCCAKQEP
Naked mole rat        EEHIKLVKEVTDFAKTCVADESAENCDKSIHTLFGDKLCAIQTLRETYGALADCCAKQEP
Guinea pig            EEHVKLVNEVTDFAKACVADESAQNCGKAIATLFGDKVCAIPSLRETYGELADCCAKEDP
Rabbit                EEHAKLVKEVTDLAKACVADESAANCDKSLHDIFGDKICALPSLRDTYGDVADCCEKKEP
Pika                  EEHVKLVNEVTEFAKTCVADESAENCGKSLHDIFGDKICAIPTLRDSYGDLAG-CAKQEP
Pig                   EEHVKLVREVTEFAKTCVADESAENCDKSIHTLFGDKLCAIPSLREHYGDLADCCEKEEP
Alpaca                DDHVKLVNEVTEFAKTCVADESAADCDKSLHILFGNKLCTVASLRETYGEMADCCEKQEA
Bactrian camel        DDHVKLVNEVTEFAKTCVADESAADCDKSLHTLFGDKLCTVASLRETYGEMADCCEKQEP
Dolphin               DEHVKLVNEITDFAKTCVADESAANCDKSLHTLFGDKLCAVASLRETYGEMADCCGKQDP
Killer whale          DEHVKLVNEITDFAKTCVADESAANCDKSLHTLFGDKLCAVASLRETYGEMADCCGKQEP
Cow                   DEHVKLVNELTEFAKTCVADESHAGCEKSLHTLFGDELCKVASLRETYGDMADCCEKQEP
Sheep                 DEHVKLVKELTEFAKTCVADESHAGCDKSLHTLFGDELCKVATLRETYGDMADCCEKQEP
Goat                  DEHVKLVKELTEFAKTCVADESHAGCDKSLHTLFGDELCKVATLRETYGDMADCCEKQEP
Horse                 EDHVKLVNEVTEFAKKCAADESAENCDKSLHTLFGDKLCTVATLRATYGELADCCEKQEP
White rhinoceros      EDHVKLVNEVNEFAKKCVADESAENCDKSLHTLFGDELCKVATNRETYGELADCCTKEEP
Cat                   EDHVKLVNEVTEFAKGCVADQSAANCEKSLHELFGDKLCTVASLRDKYGEMADCCEKKEP
Dog                   EDHVKLAKEVTEFAKACAAEESGANCDKSLHTLFGDKLCTVASLRDKYGDMADCCEKQEP
Ferret                EDHVKLVKEMTEFAKGCAADQSGADCGKSLHTLFGDKLCTVASLREKYGELADCCEKQEP
Panda                 EDHAKLAKEVTEFARGCAADQSGADCGKSLHTLFGDKLCTVASLREKYGELADCCEKQEP
Pacific walrus        EDHVKLAKEVTEFAKGCAADQSGADCGKSLHTLFGDRLCTVASLREKYGDLADCCEKQDP
Weddell seal          EDHVKLAKEVTEFAKGCAADQSGADCGKSLHTLFGDKLCTVASLREKYGELADCCEKQDP
Black flying fox      DEHSKLTNEVTEFAKTCVADESAANCDKSLHTLFGDKLCTVASLRETYGELADCCEKQDP
Megabat               DEHSKLTNEVTEFAKTCAADESAANCDKSLHTLFGDKLCTVASLRETYGELADCCEKQDP
Hedgehog              EEHVKLVKEVTDFAKGCVADESAENCEKSIHTLFGEKLCKMPSLREMYGEMADCCAKQDP
Shrew                 EDHVKLVQEVNEFAKACAADESGPNCDKSLPTLFGDKLCTVASLREKYGELADCCEKQEP
Star nosed mole       EDQLKLVTDVTEFAKTCVADESAANCDKPLQTLFGEKLCTVASVREKYGELADCCEKEEP
Hyrax                 ----------AEFAKTCVADESAADCDHSIHAIFGDKVCAVKAHPDLYADLTECCEKQEP
Elephant              DEHVQSVTAVTDLAKTCAADESAEHCGDSLHTIFGDKLCAVTAHQDTYGEFAECCGKQEP
Manatee               EDQEQSVKEVTEFAKTCVADESAENCDHSLHAIFEDKLCSVRAHSDVYAEFVECCEKKEP
Cape golden mole      EEHVKLVNEVTEFAKTCVANEAAENCDKSLHTLFGDKLCTIASLRDTYGELADCCEKQEP
Tenrec                EEHVKLVTEVTDFAKTCVADESAENCGKSLHTLFGDKLCTIASLRDMYGELADCCEKQEP
Armadillo             EEHVKLVNEVTEFAKTCVADESAENCGKSLHTLFGDELCSIANLRDSYGELADCCDKQEP
Sloth                 ------------------------------HTLLGDKACSVGSLRELYGELADCCGKQEP
Opossum               EDHVKLVDEVVQFAKGCAADETAENCGKSLHQLLGDKLCKIASLRESYGEMADCCAKEEP
Tasmanian devil       EDHVKLVSEVVEFAKGCAADESAENCGKSLHELLGDKLCKVPSLRENYGEMADCCAKEEP
Platypus              ------------------------------SLVFAEELCKADHLKDSYAELAELAECCTA
```

```
Human                 ERNECFLQHKDDNPNLPRLVRPEVDVMCTAFHDNEETFLKKYLYEIARRHPYFYAPELLF
Chimpanzee            ERNECFLQHKDDNPNLPRLVRPEVDVMCTAFHDNEGTFLKKYLYEVARRHPYFYAPELLF
Bonobo                ERNECFLQHKDDNPNLPRLVRPEVDVMCTAFHDNEGTFLKKYLYEVARRHPYFYAPELLF
Gorilla               ERNECFLQHKDDNPNLPRLVRPEVDVMCTAFHDNEETFLKKYLYEIARRHPYFYAPELLF
Orangutan             ERNECFLQHKDDNPNLPRLVRPEVDVMCTAFHDNEETFLKKYLYEIARRHPYFYAPELLF
Gibbon                ERNECFLQHKDDNPNLPPLVRPEVDVMCTAFHDNEETFLKKYLYEIARRHPYFYAPELLF
Rhesus                ERNECFLQHKDDNPNLPPLVRPEVDVMCTAFHDNEATFLKKYLYEVARRHPYFYAPELLF
Crab-eating macaque   ERNECFLQHKDDNPNLPPLVRPEVDVMCTAFHDNEATFLKKYLYEVARRHPYFYAPELLF
Olive baboon          ERNECFLQHKDDNPNLPPLVR---------------------------------------
Marmoset              ERNECFLQHKDDKPDLPPLVRPEVDVMCTAFQEHEDTFLKKYLYEVARRHPYFYAPELLF
Squirrel monkey       ERNECFLQHKDDKPDLPQLVRPEVDVMCSAFQADEDTFLKKYLYEVARRHPYFYAPELLF
Tarsier               ERNECFLEHKDDNPNLPSLIRPEADVMCTAFQENADMFLGNYLYEVARRHPYFYAPELLY
Mouse lemur           ERNQCFLAHKDDKPDLPPLERPETDVMCTSFQENENRFLG--------------------
Bushbaby              ERNQCFLDHKDDKPDLPPLVRPEVDVMCTSFQDNEKTFLGHYLYEVARRHPYFYGPELLF
Chinese hamster       ERNECFLKHKDDKPSLPALVRPEADVMCTSFQESENMFLGKYLYEVARRHPYFYAPELLY
```

Squirrel	ERFQCLIKYKEDTPTIPPLHPIDPDALCISFDESSQNVLGHFVYEVARRNPYLCGQKVMY
Golden hamster	ERNECFLKHKDDHPNLPPFVRPDAEAMCTSFQENAVTFMGHYLHEVARRHPYFYAPELLY
Mouse	ERNECFLQHKDDNPSLPPFERPEAEAMCTSFKENPTTFMGHYLHEVARRHPYFYAPELLY
Rat	ERNECFLQHKDDNPNLPPFQRPEAEAMCTSFQENPTSFLGHYLHEVARRHPYFYAPELLY
Naked mole rat	ERNECFLEHKEDNPALPPFKRPEPEAMCTSFNEDNQLFMGHYLYEVARRHPYFYAPELLY
Guinea pig	DRVECFLQHKDDNPNLPPFERPEPEALCTAFKENNDRFIGQFIFEVSRRHPYFYAPELLY
Rabbit	ERNECFLHHKDDKPDLPPFARPEADVLCKAFHDDEKAFFGHYLYEVARRHPYFYAPELLY
Pika	ERNQCFLKHKDDNPDLPPFVRPDPEVLCTAFHDNEKAFFGHYLHEVARRHPFFYAPELLY
Pig	ERNECFLQHKNDNPDIPKL-KPDPVALCADFQEDEQKFWGKYLYEIARRHPYFYAPELLY
Alpaca	ERNECFLQHKNDNPDLPKL-KPEPEALCTAFQENEKRFGGKYLYEIARRHPYFYAPELLY
Bactrian camel	ERNECFLQHKSDNPDLPKL-KPEPEALCTAFQENEKRFGGKYLYEIARRHPYFYAPELLY
Dolphin	ERNECLLKHKDDNPDLPKL-KPDPETFCTEFKENEQKFWGKYLYEIARRHPYFYAPELLY
Killer whale	ERNECLLKHKDDNPDLPKL-KPDPETLCTEFKENEQKFWGKYLYEIARRHPYFYAPELLY
Cow	ERNECFLSHKDDSPDLPKL-KPDPNTLCDEFKADEKKFWGKYLYEIARRHPYFYAPELLY
Sheep	ERNECFLNHKDDSPDLPKL-KPEPDTLCAEFKADEKKFWGKYLYEVARRHPYFYAPELLY
Goat	ERNECFLKHKDDSPDLPKL-KPEPDTLCAEFKADEKKFWGKYLYEVARRHPYFYAPELLY
Horse	ERNECFLTHKDDHPNLPKL-KPEPDAQCAAFQEDPDKFLGKYLYEVARRHPYFYGPELLF
White rhinoceros	ERNECFLQHKDDSPNFPPL-KAEADVLCAQFQADEQSFLGKYLYEIARRHPYFYAPELLY
Cat	ERNECFLQHKDDNPGFGQLVTPEADAMCTAFHENEQRFLGKYLYEIARRHPYFYAPELLY
Dog	DRNECFLAHKDDNPGFPPLVAPEPDALCAAFQDNEQLFLGKYLYEIARRHPYFYAPELLY
Ferret	ERNECFLTHKDDNPGFPPLVTPDPEVMCTAFQENEQQFLGKYLYEVARRHPYFYAPELLY
Panda	ERNECFLKHKDDNPGFPPLVTPEPDALCAAFQENEQRFLGKYLYEVARRHPYFYGPELLY
Pacific walrus	ERNECFLSHKDDNPGFPPLVTPEPDAICTAFQENEQKFLGKYLYEVARRHPYFYAPELLY
Weddell seal	ERNECFLTHKDDNPGFPPLVTPEPDAMCAAFQESEQKFLGKYLYEVARRHPYFYAPELLY
Black flying fox	ERNECFLKHKDDDPNLPPVVKPEPEALCTAFQENNNKFLENYLYEVARRHPYFYGPELLY
Megabat	ERNECFLKHKDDDPNLPPIVKPEPEALCTAFQENNNKFLENYLYEVSRRHPYFYGPELLY
Hedgehog	ERIDCFVQHKDDSPNLPPVVRPDPEVMCTAFQEDQLKFAGSYLYEVARRHPYFYAPELLY
Shrew	ERADCFASHRDDNPGFPLMVRPPVDELCASYQADAQMFAGKYLYEVARRYPYFYAPELLY
Star nosed mole	GRHECFLQHKDDNPGLPRFTRPAPDALCSAFQENEKKFAAHYLYEVARRHPYFYAPELLY
Hyrax	ERNECFQKHKDDNPDLPPLVRPTADALCASFHENEKNLLGHYLYEVARRHPYFYAPELIY
Elephant	ERNECFLKHKDDNPALPPLVRPPADALCASFEENERKFFGVYLYEVARRHPYFYAPELIY
Manatee	ERHNCFLQHKDDNPPVPPFVRGSADDLCTAFHENEKRFFGKYIYEVARRHPYFYAPELIY
Cape golden mole	ERNNCFLTHKDDNPGLPQLVRPPADDLCAAFQADEDKFFGEYLYQVARRHPYFYAPELLF
Tenrec	ERNECFLKHRDESPALPPIERPSADVLCTAFEDNEERFFGKYIYEVSRRHPYFYGPELLY
Armadillo	ERNECFLKHKDDNPDLPPLVRPDAEVMCTAYQENEQDFVEIYVHGVARRHPFFYGPELLY
Sloth	ERNQCFLKHKDDNPDLPPFARPEVAVICASFQENEEKFLGHYLYEISRRNPFFYAPELIH
Opossum	ERNQCFLSHKDDHPDLPKIVAPEPDKLCQEFQENENKVMGYYLHQVARRHPYFYAPALLA
Tasmanian devil	ERHRCFLSHKDDQPNLPKIEAPEPETLCKNFQENENRVLGFYLYEVARRHPYFYAPALLA
Platypus	EKAECLLKHKDETPEVPPFTRPEPAVLCKEYEDNRNQFLG--------------------

Human	FAKRYKAAFTECCQAADKAACLLPKLDELRDEGKASSAKQRLKCASLQKFGERAFKAWAV
Chimpanzee	FAERYKAAFTECCQAADKAACLLPKLDELRDEGKASSAKQRLKCASLQKFGERAFKAWAV
Bonobo	FAERYKAAFTECCQAADKAACLLPKLDELRDEGKASSAKQRLKCASLQKFGERAFKAWAV
Gorilla	FAARYKAAFTECCQAADKAACLLPKLDELRDEGKASSAKQRLKCASLQKFGERAFKAWAV
Orangutan	FAVRYKAAFTECCQAADKAACLLPKLDELRDEGKASSAKQRLKCASLQKFGERAFKAWAV
Gibbon	FAERYKAAFTECCQAADKAACLLPKLDGLRDEGKASSAKQRLKCASLQKFGERAFKAWAV
Rhesus	FAARYKAAFAECCQAADKAACLLPKLDELRDEGKASSAKQRLKCASLQKFGDRAFKAWAV
Crab-eating macaque	FAARYKAAFAECCQAADKAACLLPKLDELRDQGKASSAKQRLKCASLQKFGDRAFKAWAV
Olive baboon	-------------------------LDELRDQGKASSAKQRLKCASLQKFGDRAFKAWAV
Marmoset	FAQKYKAAFTECCQAADKGACLLPKLDELRDQGKASSAKQRLKCASLQKFGERAFKAWSV
Squirrel monkey	FAQKYKAAFTECCQAADKDACLTPKLDELQSEGKASSAKQRLKCASLQKFGERAFKAWVV
Tarsier	YGQKYKAVFTECCQAADKAACLFPK---------------------------------AV
Mouse lemur	-------------------------LDELKEEGMASSARQRLKCSSLEKFGERAFKAWAV
Bushbaby	FAEKYKAAFTECCQAADKAACLLPKLDTLKEEGMAASAKQRLKCSSLEKFGDRAFKAWAV
Chinese hamster	YAQKYKTALKECCAEADKAACLTPKLDALKEKALFSSAKQRLKCTSIQKFGDRAFKAWAV
Squirrel	FAEKYKGFLTECCKAADKGECLTQKTENLKKTIMLSSAKDRFKCSVLEKYEERGLKAWLI
Golden hamster	YAEKYSAIMTECCGEADKAACITPKLDALKEKALASSVNQRLKCSSLQRFGQRAFKAWAV
Mouse	YAEQYNEILTQCCAEADKESCLTPKLDGVKEKALVSSVRQRMKCSSMQKFGERAFKAWAV
Rat	YAEKYNEVLTQCCTESDKAACLTPKLDAVKEKALVAAVRQRMKCSSMQRFGERAFKAWAV
Naked mole rat	YAERYMDVLTECCQAADKATCLTPKLDALKEKALTSAAQQRLKCASIQKFGERAFKAWAL
Guinea pig	YAEKYKNALTECCEAADKAACLTPKLDAIKEKALVSSAQQRLKCASLQKFGERAFKAWSV
Rabbit	YAQKYKAILTECCEAADKGACLTPKLDALKEKALISAAQERLRCASIQKFGDRAYKAWAL
Pika	YAEKYKAILTECCSAADKGACLTPKLDALKEKALTDAFNERLKCASIQKFGDRAFKAWAL
Pig	YAIIYKDVFSECCQAADKAACLLPKIEHLREKVLTSAAKQRLKCASIQKFGERAFKAWSL
Alpaca	YAHQYKHVFEECCKEADKAACLLPKIDLEKG---TSSARQRLRCTSIQ-FGEEALKAWSV

```
Bactrian camel        YAHQYKHVFEECCKDADKAACLLPKLDALKERILASSARQRLRCTSIQKFGDRALKAWSV
Dolphin               FAHQYKGVFGECCQAADKGACLIPKI--L-EE--ASSARQRLKCTSIQKFGERALKAWSV
Killer whale          FAHQYKGVFAECCQAADKGACLIPKIETVREEVLASSARQRLKCTSIQKFGERALKAWSV
Cow                   YANKYNGVFQECCQAEDKGACLLPKIETMREKVLTSSARQRLRCASIQKFGERALKAWSV
Sheep                 YANKYNGVFQECCQAEDKGACLLPKIDAMREKVLASSARQRLRCASIQKFGERALKAWSV
Goat                  YANKYNGVFQECCQAEDKGACLLPKIETMREKVLASSARQRLRCASIQKFGERALKAWSV
Horse                 HAEEYKADFTECCPADDKAGCLIPKLDALKERILLSSAKERLKCSSFQNFGERAVKAWSV
White rhinoceros      YAEEYRDDLKECCQADDKAACLLPKIAVLRDRVLTSSAKERLKCASLQKFGERALKAWTV
Cat                   YAEEYKGVFTECCEAADKAACLTPKVDALREKVLASSAKERLKCASLQKFGERAFKAWSV
Dog                   YAQQYKGVFAECCQAADKAACLGPKIEALREKVLLSSAKERFKCASLQKFGDRAFKAWSV
Ferret                YTQQYKGVFTECCQAADKAACLTPKTEALREKVLVSSARERFKCASLQKFGDRAFKAWSV
Panda                 YAQQYKGVFAECCQAADKAACLTPKIDDLREKVLVSSAKERFKCASLQKFGDRAFKAWSI
Pacific walrus        YAQQYKQVFAECCQAADKAACLTPKIDALREKVLVSSAKERFKCASLQKFGDRAFKAWSV
Weddell seal          YAQQYKQVFAECCQAADKAACLTPKIDALREKVLVSSAKERFKCASLQKFGDRAFKAWSI
Black flying fox      YVKQYKAILTECCQAADKATCLAPKAKVLKEKLLASSAKQRHKCASIQKFGERAFKAWSI
Megabat               YVKKYKAILTECCQAADKATCLAPKAKVLKEKLQASSAKQRHRCAS------------SM
Hedgehog              FAAKYKDVLAECCQAADKGACLTPKMDELKGKALVSAAQQRFKCAGLQKFGERAFKAYAV
Shrew                 YAQKYKDALAECCSAADKAACLTPKIDDLKESVMTSGAKQRFKCAGIEKFGERAFKAWAV
Star nosed mole       YAKKYRQILAECCQADDKGACLTPKIDELKEVVLTSSAKQRFKCASIQKFGERAFKAWSV
Hyrax                 YSHEYKDVLKECCEAADKDACLTPK-----------------------------------
Elephant              YSEKYKDILTECCHADDKAACLKPKIDALKDTVLASAARQRLKCANIHKFGERAFKAWAV
Manatee               YSYKYREILAECCEAADKAACLTPKINSLKEKVLTASAKERLRCVSIEKFGERAFKAWGV
Cape golden mole      FSQKYKAVLTECCQAADKADCLTPKLDLLKEAVLTSSAKQRLKCANIEKFGERAFKAWAV
Tenrec                ISAKYKAALTECCHVADKAACLTPKLDELKEHALISSAKQRLRCASIEKFGPRAFNAWAV
Armadillo             YAQQYKVAFKECCQAADKAACLTPKMDALKQSALISSAKQRFKCAGIEKFGERAFKAWGV
Sloth                 YTHRYKDALKECCSAEDKAACLAPKLEKLKEKVLFSAAKQRLKCSNLEKFGERPFKAWAV
Opossum               YAIQYRDAVRECCAAADKATCLNDKLTDLREKVLSAGAKQRFRCASLDKFGERAVKAGLV
Tasmanian devil       YAHQYKEAVKGCCQEADKGACFDNKLSVLRDKVISSGAKQRFRCSSLDEFGERAVKAGLV
Platypus              ---------------------LSLQLPELKDKVLVTTAKQRFRCAAYQKFGERAMKAVLV


Human                 ARLSQRFPKAEFAEVSKLVTDLTKVHTECCHGDLLECADDRADLAKYICENQDSISSKLK
Chimpanzee            ARLSQRFPKAEFAEVSKLVTDLTKVHTECCHGDLLECADDRADLAKYICENQDSISSKLK
Bonobo                ARLSQRFPKAEFAEVSKLVTDLTKVHTECCHGDLLECADDRADLAKYICENQDSISSKLK
Gorilla               ARLSQRFPKAEFAEVSKLVTDLTKVHTECCHGDLLECADDRADLAKYICENQDSISSKLK
Orangutan             ARLSQRFPKAEFAEVSKLVTDLTKVHTECCHGDLLECADDRADLAKYICENQDSISSKLK
Gibbon                ARLSQRFPKAEFAEVSKLVTDLTKVHTECCHGDLLECADDRADLAKYICENQDSISSKLK
Rhesus                ARLSQKFPKAEFAEVSKLVTDLTKVHTECCHGDLLECADDRADLAKYMCENQDSISSKLK
Crab-eating macaque   ARLSQKFPKAEFAEVSKLVTDLTKVHTECCHGDLLECADDRADLAKYMCENQDSISSKLK
Olive baboon          ARLSQKFPKAEFAEVSKLVTDLTKVHTECCHGDLLECADDRADLAKYMCENQDSISSKLK
Marmoset              ARVSQRFPKADFTEVTKVVTDLTKVHTECCHGDLLECADDRAELAKYMCENQDSLSSKLK
Squirrel monkey       ARLSQKFPKADFAEVTKVVTDLTKVHTECCHGDLLECADDRADLAKYMCDNQDSLSSKLK
Tarsier               ARMSQKFPKADFAEVTKLVTDFTKVHKECCHGDLLECADDRADLAKYMCENQDSVSSKLK
Mouse lemur           ARLSQRFPKADFAEVTKLVKDLTKIHTECCHGDLLECADDRAELAKYMCEHQDSLSSKLK
Bushbaby              ARLSQRFPKAEFAEVSKLVTDLTKVHTECCHGDLLECADDRADLAKYMCENQDSLSSKLK
Chinese hamster       ALLSQKYPKADFAEITKIAKDLTKVHTECCHGDLLECADDRADLAKYMCDNQDSISSKLK
Squirrel              SRLSRKFPQAEFTEITKIATDLTKIIKESCSGDLLESTHDRVTLANYICENQDKISKKVG
Golden hamster        ARMSQKFPKADFAEITKLATDLTKLTEECCHGDLLECADDRAELAKYMCENQASISSKLQ
Mouse                 ARLSQTFPNADFAEITKLATDLTKVNKECCHGDLLECADDRAELAKYMCENQATISSKLQ
Rat                   ARMSQRFPNAEFAEITKLATDLTKINKECCHGDLLECADDRAELAKYMCENQATISSKLQ
Naked mole rat        ARMSQKFCKAEFIEISRIVNDLTKVNKECCRGDLLECAADRASLVKYICANQETISSKLK
Guinea pig            ARLSQKFPKAEFAEISTIVTSLTKVTKECCHGDLLECADDRQELAKYMCEHQDSISSKLK
Rabbit                VRLSQRFPKADFTDISKIVTDLTKVHKECCHGDLLECADDRADLAKYMCEHQETISSHLK
Pika                  SRLSQEFPKADFMEISKLVTDLTKVHKECCQGDLLECADDRAELTKYICEHQDKISAQLK
Pig                   ARLSQRFPKADFTEISKIVTDLAKVHKECCHGDLLECADDRADLAKYICENQDTISTKLK
Alpaca                GHLSQKFPKADFAEISKIVTDLTKIHKECCHGDLLECADDRADLAKYFCDNQETISSKLK
Bactrian camel        GHLSQKFPKADFAEISKIVTDLTKIHKECCQGDLLECADDRADLAKYFCDNQETISSKLK
Dolphin               ARLSQKFPKADFAEVSKIVTDLTKVHKECCHGDLLECADDRADLAKYICENQATISSKLQ
Killer whale          ARLSQKFPKADFAEVSKIVTDLTKVHKECCHGDLLECADDRADLAKYICENQATISSKLQ
Cow                   ARLSQKFPKAEFVEVTKLVTDLTKVHKECCHGDLLECADDRADLAKYICDNQDTISSKLK
Sheep                 ARLSQKFPKADFTDVTKIVTDLTKVHKECCHGDLLECADDRADLAKYICDHQDALSSKLK
Goat                  ARLSQKFPKADFTDVTKIVTDLTKVHKECCHGDLLECADDRADLAKYICDHQDTLSSKLK
Horse                 ARLSQKFPKADFAEVSKIVTDLTKVHKECCHGDLLECADDRADLAKYICEHQDSISGKLK
White rhinoceros      ARLSQKFPKADFAEISKLVTDLAKIHTECCHGDLLECADDRADLAKYFCDNQDSISSKLK
Cat                   ARLSQKFPKAEFAEISKLVTDLAKIHKECCHGDLLECADDRADLAKYICENQDSISTKLK
Dog                   ARLSQRFPKADFAEISKVVTDLTKVHKECCHGDLLECADDRADLAKYMCENQDSISTKLK
```

```
Ferret               ARLSQKFPKADFAEVSKLVTDLTKVHKECCHGDLLECADDRADMAKYICENQDSISTKLK
Panda                ARLSQKFPKADFAEVSKVVTDLTKVHKECCHGDLLECADDRADLAKYMCENQDSISSKLK
Pacific walrus       ARMSQKFPKADFAEVTKLVTDLTKIHKECCHGDLLECADDRADLAKYMCENQDSISTKMK
Weddell seal         ALMSQKFPKADFAEVSKLVTDLTKIHKECCHGDLLECADDRADLAKYMCENQDSISSKMK
Black flying fox     ARLSQRFPKADFMDLSKLVTDLSKIHKECCHGDLLECADDREDLAKYVCDNQDSFSSKLK
Megabat              ARVSQRFPKADFMELSKIVSDLSKIHKECCHGDLLECADDRGDLVKYMCDNQDSFSSKLK
Hedgehog             ARMSQKFPAADFMEISKIVTDLTKIHKECCHGDLLECADDRADLVKYTCENQDTISSKLK
Shrew                ARLSQKFPNADFAEISKIVDTLTKINKECCHGDLLECADDRVELGKYMCDNKDSISSKLG
Star nosed mole      SRLSQKFPKADFAEISKIVGDLTKVNKECCEGDLLECADDREALAKYICENQDTISSKLK
Hyrax                ----------------------------------------AELAKHICDHQGELSSKLK
Elephant             THLSQRFPKADFAEVSKLATDLGKVYQECCHGDLLECADDRADLAKYICDNQETLSSKLK
Manatee              SRLSQRFPKAEFTEISKIVADLAKIHQECCHGDLLECADDRADLAKYICDNQDSLSSKLK
Cape golden mole     SQMSQKFPKAEFSDITKVVLDLAKIHQECCHGDLLECADDRADLAKYMCENQDLFSSKLK
Tenrec               ARLSQRFPKADFPQISQLVTGLAKIHTECCHGDLLECADDRADLAKYTCENQETISSKLK
Armadillo            SSTSQKFPKTDFAEVTKIVTDLTKIHKECCNGDLLECADDRADLAKYICDNQDSISSKLK
Sloth                ATITQSFSHADFAEITKLVTDYTKINQECCQGDLLKCADDREDLVKYICENQGSISNKLK
Opossum              ARLSQKFPKADFAEIHKIVEDLANVHKECCHGDLLECADDRAALSDYVCQNKDSISSKLT
Tasmanian devil      ARLSQKFPKADFAEIHKIVEDLAKIHTECCHGDLLECADDRVALSEYLCNNKETISTKLG
Platypus             AKTSQRFPKADFGDIHKVVEDLAKVVKECCHGDQLECMEDRADLTKYLCDNQDTFSSKLK
```

```
Human                ECCEKPLLEKSHCIAEVENDEMPADLPSLAADFVESKDVCKNYAEAKDVFLGMFLYEYAR
Chimpanzee           ECCEKPLLEKSHCLAEVENDEMPADLPSLAADFVESKEVCKNYAEAKDVFLGMFLYEYAR
Bonobo               ECCEKPLLEKSHCLAEVENDEMPADLPSLAADFVESKEVCKNYAEAKDVFLGMFLYEYAR
Gorilla              ECCEKPLLEKSHCLAEVENDEMPADLPSLAADFVESKDVCKNYAEAKDVFLGMFLYEYAR
Orangutan            ECCEKPLLEKSHCLAEVENDEMPADLPSLAADFVESKDVCKNYAEAKDVFLGMFLYEYAR
Gibbon               ECCEKPLLEKSHCLAEVENDEMPADLPSLAADFVESKDVCKNYAEAKDVFLGMFLYEYAR
Rhesus               ECCDKPLLEKSHCLAEVENDEMPADLPSLAADYVESKDVCKNYAEAKDVFLGMFLYEYAR
Crab-eating macaque  ECCDKPLLEKSHCLAEVENDEMPADLPSLAADYVESKDVCKNYAEAKDVFLGMFLYEYAR
Olive baboon         ECCDKPLLEKSHCLAEVENDEMPADLPSLAADYVESKEVCKNYAEAKDVFLGMFLYEYAR
Marmoset             ECCEKPLLEKSHCLAHVENDEMPADLHALTDDYVESKDVCKNYAEAKDVFMGMFLYEYSR
Squirrel monkey      ECCDKPLLEKSHCLSHAENDDMPADLPALAAEFVESKDVCKNYAEAKDVFLGMFLYEYSR
Tarsier              ECCDKPLLEKSHCLSEVENDDMPANLPALTTDFVEDKEVCKNYAEAKDVFLG--------
Mouse lemur          ECCEKPVLEKSHCLAHVENDDIPADLPSIAADFVEDKEVCKNYAEAKDVFLGTFLNEYSR
Bushbaby             ECCEKPLLEKSHCLAHVENDDLPTDLAPLAADFVEDKDVCKNYAEAKDVFLGTFLYEYSR
Chinese hamster      ECCDKPLLEKAHCIAETEHDDPPADLAALEADFVEDKDVCKNYAEAKDVFLGTFLYEYAR
Squirrel             ECCVKPLLERYHCIIDLEEDDKLADLPALTADYAEDKDVCKNYAEAKDIFLGMFLYEYSR
Golden hamster       ACCDKPVLKKSHCLSEVENDDLPADLPSLAADFVEDKEVCKNYAEAKDVFLGTFLYEYAR
Mouse                TCCDKPLLKKAHCLSEVEHDTMPADLPAIAADFVEDQEVCKNYAEAKDVFLGTFLYEYSR
Rat                  ACCDKPVLQKSQCLAEIEHDNIPADLPSIAADFVEDKEVCKNYAEAKDVFLGTFLYEYSR
Naked mole rat       ECCEKPLLEKAHCIMEAERDELPDGLPELTTDFIEDRDVCTHYTGAKDVFLGTFLFEYSR
Guinea pig           ECCVKPTLQKAHCILEIQRDELPTELPDLAVDFVEDKEVCKNFAEAKDVFLGTFLYEYSR
Rabbit               ECCDKPILEKAHCIYGLHNDETPAGLPAVAEEFVEDKDVCKNYEEAKDLFLGKFLYEYSR
Pika                 ECCDKPLLQKSHCIANVHHDDTPADLPTLASLFVEDKDVCKNYQETKDIFLGSFLYENAR
Pig                  ECCDKPLLEKSHCIAEAKRDELPADLNPLEHDFVEDKEVCKNYKEAKDVFLGTFLYEYSR
Alpaca               ECCEKPLLEKSHCIHEAERDEIPENLPAITDQFAEDKDVCKHYKEEKDVFLGMFLHEYAR
Bactrian camel       ECCEKPLLEKSHCIHEAERDEMPENLPAITEQFAEDKDVCKHYTEEKDVFLGMFLHEYAR
Dolphin              KCCHKPLLEKSHCISEVEKDELPENLSLLAADFAEDKEVCKNYNEAKDVFLGTFLYDYAR
Killer whale         KCCDKPLLEKSHCISEVEKDELPENLSPLAADFAEDKEVCKNYNEAKDVFLGTFLYEYAR
Cow                  ECCDKPLLEKSHCIAEVEKDAIPENLPPLTADFAEDKDVCKNYQEAKDAFLGSFLYEYSR
Sheep                ECCDKPVLEKSHCIAEVDKDAVPENLPPLTADFAEDKEVCKNYQEAKDVFLGSFLYEYSR
Goat                 ECCDKPVLEKSHCIAEIDKDAVPENLPPLTADFAEDKEVCKNYQEAKDVFLGSFLYEYSR
Horse                ACCDKPLLQKSHCIAEVKEDDLPSDLPALAADFAEDKEICKHYKDAKDVFLGTFLYEYSR
White rhinoceros     ACCDKPLLQKSHCIAEFEGDELPSDLPPLAADFAEDKEVCKHFQDAKDVFLGTFLYEYAR
Cat                  ECCGKPVLEKSHCISEVERDELPADLPPLAADFVEDKEVCKNYQEAKDVFLGTFLYEYSR
Dog                  ECCDKPVLEKSQCLAEVERDELPGDLPSLAADFVEDKEVCKNYQEAKDVFLGTFLYEYSR
Ferret               ECCDKPLLEKSQCLAEVERDELPGDLTPLAADFVEDKEVCKNYQEAKDVFLGTFLYEYSR
Panda                ECCDKPVLEKSQCLSEVEKDELPGDLPLLAADFVEDKEVCKNYQEAKDVFLGTFLYEYSR
Pacific walrus       ECCDKPLLEKSHCLTEVERDELPGDLSPLAADFVEDKEVCKNYQEAKDVFLGTFLYEYSR
Weddell seal         ECCDKPLLEKSHCLTEVERDELPGDLSPIAADFVEDKEVCKNYQEAKDVFLGTFLYEYSR
Black flying fox     ECCDKPLLEKSHCISELENDDLPNDLPSITTDFVEDKDVCKNYKEAKDVFLGTFLYEYSR
Megabat              ECCDKPLLEKSHCLSEVENDDLPNDLPSITTDFVDDKDVCKNYKEAKDAFLGTFLYEYSR
Hedgehog             SCCDKPLLEK-HCIAEAERDDLPTNLPLLAADFAVDKVVCKNYPEAKNIFLDTFLHEYAR
Shrew                KCCEKPLLEKGHCIAELEKDDMPADLSPIEADFVEDKEVCKNYAEAKDVFLGTFLYELSR
Star nosed mole      ECCGKPIIEKSHCIYELEKDEVPGDLPSIAVDFVDDKEVCKNYQEAKDVFLGTFLYEYSR
Hyrax                TCCDKPVLEKSHCIVELEKDEPPADLHPLAETFVDDNEVCKHYQEAKDAFLGSFLHEYAR
```

```
Elephant              ECCDKPVLAKSHCIAELDKDDPPADLPSIVPDYVEDKDVCKNYQEAKDIFLGTFLYEYSR
Manatee               GCCDKPVLEKSHCILEVEKDDVPAGLPPIAETYVEDKDVCKNYHEAKDIFLGTFLYEYSR
Cape golden mole      DCCAKSVLEKSHCISEVEKDDIPADLPSIAVTYVDDKEVCKNYAETKDLFLGMFLYEYAR
Tenrec                ECCEKPVLEKSHCIAEVENDDIPDDLTPLAADFVEDKEVCKNYEEERDIFMGTFLHEYAR
Armadillo             KCCDQPLSEKSHCIYELEKDEAPADLPPINVDFVDDKDVCKNYAEAKDVFLGMFLYEYAR
Sloth                 QCCDKPLLEKSHCILEIEKDDLIADLPALTTDFADDKEVCKHYAEAKDVFLGTFLYELSR
Opossum               KCCDKSLVEKSQCIADLENDDLPADLPDFDETYVTSKEACQNYKEAKDLFLANYLYDSAR
Tasmanian devil       ECCDKPLIEKSQCIADLGNDAIPDGLPDLLAEYVNTKDACQNYKEAKDLFLATYLYDNGR
Platypus              TCCDKPLVVRSQCVVDLENDDTPADLPTMASIYVEDKEVCNNYAGAKDLFLATFLYDYAR


Human                 RHPDYSVVLLLRLAKTYETTLEKCCAAADPHECYAKVFDEFKPLVEEPQNLIKQNCELFE
Chimpanzee            RHPDYSVVLLLRLAKTYETTLEKCCAAADPHECYAKVFDEFKPLVEEPQNLIKQNCELFE
Bonobo                RHPDYSVVLLLRLAKTYETTLEKCCAAADPHECYAKVFDEFKPLVEEPQNLIKQNCELFE
Gorilla               RHPDYSVVLLLRLAKTYETTLEKCCAAADPHECYAKVFDEFKPLVEEPQNLIKQNCELFE
Orangutan             RHPDYSVVLLLRLAKTYETTLEKCCAAADPHECYAKVFDEFKPLVEEPQNLIKQNCELFE
Gibbon                RHPDYSVVLLLRLAKAYETTLEKCCAAADPHECYAKVFDEFKPLVEEPQNLIKHNCELFE
Rhesus                RHPDYSVMLLLRLAKAYEATLEKCCAAADPHECYAKVFDEFQPLVEEPQNLVKQNCELFE
Crab-eating macaque   RHPDYSVMLLLRLAKAYEATLEKCCAAADPHECYAKVFDEFQPLVEEPQNLVKQNCELFE
Olive baboon          RHPDYSVMLLLRLAKAYEATLEKCCAAADPHECYAKVFDEFQPLVEEPQNLVKQNCELFE
Marmoset              RHPDYSAMLLLRLAKAYEATLEKCCATADPHGCYAKVFDEFQPLVEDPQNLVKHNCELFE
Squirrel monkey       RHPDYSVMLLLRLAKAYEATLEKCCATADPHGCYAKVFDEFQPLVEDPQNLVKHNCELFE
Tarsier               -------------------------------------------------NLVKQNCEL--
Mouse lemur           RHPEQSPFVLLRVAKTYEATLEKCCAAADPHACYANV-----------------------
Bushbaby              RHPEYSVFLLLRLAKHYEATLEKCCATDDPHTCYAKVLDELQHDVEEPQNLVKQNCELFE
Chinese hamster       RHHGDSTLMVLRI-KAYEATLERCCATADPPSCYGKVMEEFKALSEETHNLVKQNCELFE
Squirrel              RHPEYGSLLLLRIAKAYEAKLEKCCAEADPPACYGKVFEEFEPLATEPQNVVKQNCDLYE
Golden hamster        RHPDYSVALLLRLAKKYEATLEKCCAEADPSACYGKVLDEFQPLVEEPKNLVKANCELFE
Mouse                 RHPDYSVSLLLRLAKKYEATLEKCCAEANPPACYGTVLAEFQPLVEEPKNLVKTNCDLYE
Rat                   RHPDYSVSLLLRLAKKYEATLEKCCAEGDPPACYGTVLAEFQPLVEEPKNLVKTNCELYE
Naked mole rat        RNPDYSIGMLLRLAKAYEAKLEKCCAEADPPACYATAFHELQPLIDEPKNLVQQNCELYE
Guinea pig            RHPEYSIGMLLRIAKGYEAKLEKCCAEADPHACYAKVFDRLQPLIDEPKKLVQQNCELFD
Rabbit                RHPDYSVVLLLRLGKAYEATLKKCCATDDPHACYAKVLDEFQPLVDEPKNLVKQNCELYE
Pika                  RHPDYPVTVLLKLAKVYEATLQKCCATDDPHACYAKVLDEFQPLVDEPKALIKQNCDLFD
Pig                   RHPDYSVSLLLRIAKIYEATLEDCCAKEDPPACYATVFDKFQPLVDEPKNLIKQNCELFE
Alpaca                RHPEYAVSLMLRIAKEYEATLEDCCAKDDPHACYATVFDKLQHLADEPQNLVKQNCELFE
Bactrian camel        RHPEYAVSLLLRIAKEYEATLEDCCAKDDPHACYATVFDKLQHLADEPQNLVKQNCELFE
Dolphin               RHPEYSVSLLLRIAKGYEATLEDCCAKDDPPACYATVFEKLRPLVEEPKNLIKQNCELFE
Killer whale          RHPEYSVSLLLRIAKGYEATLEDCCAKDDPPACYATVFEKLQPLVEEPKNLIKQNCELFE
Cow                   RHPEYAVSVLLRLAKEYEATLEECCAKDDPHACYSTVFDKLKHLVDEPQNLIKQNCDQFE
Sheep                 RHPEYAVSVLLRLAKEYEATLEDCCAKEDPHACYATVFDKLKHLVDEPQNLIKKNCELFE
Goat                  RHPEYAVSVLLRLAKEYEATLEDCCAKEDPHACYATVFDKLKHLVDEPQNLIKKNCELFE
Horse                 RHPDYSVSLLLRIAKTYEATLEKCCAEADPPACYATVFDQFTPLVEEPKSLVKKNCDLFE
White rhinoceros      RHPDYSVSLLLRIAKQYEATLEKCCVADDPPACYGKVFDELKTLVDEPVKLVKENCETYE
Cat                   RHPEYSVSLLLRLAKEYEATLEKCCATDDPPACYAHVFDEFKPLVEEPHNLVKTNCELFE
Dog                   RHPEYSVSLLLRLAKEYEATLEKCCATDDPPTCYAKVLDEFKPLVDEPQNLVKTNCELFE
Ferret                RHPEYSVSLLLRLAKEYEATLEKCCATADPPSCYGKVFDEFKPLVEEPQNLVKVNCELFE
Panda                 RHPEYSVSLLLRLAKEYEATLEKCCATDDPPTCYGKVLDEFKPLVEEPQNLVKANCELFE
Pacific walrus        RHPEYAISLLLRLAKEYEATLEKCCATDDPPSCYGKVLDEFKPLVEEPQNLVKTNCELFE
Weddell seal          RHPEYAISLLLRLAKEYEATLEKCCATDDPPTCYGKVLDEFKPLVEEPQNLVKTNCELFE
Black flying fox      RHPEYAVSLLLRIAKGYEATLERCCATDDAHACYSKVFDELQPLVDEPQKLMKRNCELFE
Megabat               RHPEYAISMLLRIAKEYEATLERCCATDDAHACYSKVFDELQPLVDEPQKLMKQNCEFFE
Hedgehog              RHHDYSVSLLLRLAKLYEAKLEKCCPTADPHMCYGKVFDDLKPLVEEPQKLVKENCELFS
Shrew                 RHPEYSVVMLLRLAKGYEAVLEKCCATGDPQECYSRV-DELKPLIDEPQNLVKHNCELYG
Star nosed mole       RHPEYSVLMLLRIAKGYEATLEKCCATDDPHSCYSKVFDELKPLVDEPQTLVKENCALFE
Hyrax                 RHLDYSVIMLLRAAKDYEGTLERCCATDDPHACYAKVLDEIQ-LNDEPHNVVQTNCEKFE
Elephant              RHPEYSAFLLLRIAKEYEATLERCCAAADPHACYAKVLDAFTPLVEEPQGLVKQNCEHFE
Manatee               RHPDYSMFLLLRIAKAYEATLEKCCAADDPHACYAKVLDEFKPLIEEPQNLVKQNCEDFE
Cape golden mole      RHPEYSAFLLGRLVKKYEATLEKCCATDDPPTCYGKVLDELNTLVEEPQRVVKQNCELFD
Tenrec                RHHDYSVSLLLRLAKLYEAKLEKCCPTADPHMCYGKVLEEMKPLIEEPQKLVKKNCDLFN
Armadillo             RHLDYPVALLLRLTKVYEAKLEKCCATEDPHACYSNVLTEFQPLIDEPHDMVKQNCELFD
Sloth                 RHLDFPITLLLRLAKIYEAKLEKCCATADPHACYSKVFDEFQPIIDEPKNLVKHNCELFE
Opossum               RGANLAVTTLLRLAKGYETTLEECCATDDPPSCYAKVTLQRKAIIDDSNNLVKQNCDIFE
Tasmanian devil       RHPELAVTTLLRIAKDYETTLEECCATADPPTCYAKAGEHRKNFIDESQNLIKENCDLFD
Platypus              RHTDFSSVLLLRVAKGYQAKLTECCAGDDPNACLAKAAEELKTYVAETKALVKDNCDLYD
```

Human	QLGEYKFQNALLVRYTKKVPQVSTPTLVEVSRNLGKVGSKCCKHPEAKRMPCAEDYLSVV
Chimpanzee	QLGEYKFQNALLVRYTKKVPQVSTPTLVEVSRNLGKVGSKCCKHPEAKRMPCAEDYLSVV
Bonobo	QLGEYKFQNALLVRYTKKVPQVSTPTLVEVSRNLGKVGSKCCKHPEAKRMPCAEDYLSVV
Gorilla	QLGEYKFQNALLVRYTKKVPQVSTPTLVEVSRNLGKVGSKCCKHPEAKRMPCAEDYLSVV
Orangutan	QLGEYKFQNELLVRYTKKVPQVSTPTLVEVSRNLGKVGSKCCKHPEPKRMPCAEDYLSVV
Gibbon	QLGEYKFQNELLVRYTKKVPQVSTPTLVEVSRNLGKVGSKCCKHPEAQRMPCTEDYLSVV
Rhesus	QLGEYKFQNALLVRYTKKVPQVSTPTLVEVSRNLGKVGAKCCKLPEAKRMPCAEDYLSVV
Crab-eating macaque	QLGEYKFQNALLVRYTKKVPQVSTPTLVEVSRNLGKVGAKCCKLPEAKRMPCAEDYLSVV
Olive baboon	QLGEYKFQNALLVRYTKKVPQVSTPTLVEVSRNLGKVGAKCCKLPEAKRMPCAEDYLSVV
Marmoset	QFGEYKFQNELLVRYTKKVPQVSTPTLVEVSRNLGKVGTKCCKQPEAKRMPCVEDYLSVV
Squirrel monkey	QLGEYKFQNELLVRYTQKVPQVSTPTLVEVARNLGKVGTKCCKQPEAKRMPCAEDYLSVV
Tarsier	EQGEYNFQNALLVRYTKKVPQVSTPTLVEVSRNLGRVGTKCCKLSETQRMSCAEDY----
Mouse lemur	----------LIVRYTKKAPQVSTPTLVELSRKLGKGGTKCCKLDESKRMGCTEDFLALV
Bushbaby	KLGEYGFQNALLVRYTKKVPQVSTPTLVEVSRSLGRVGSKCCKMDEAKRMGCAEDYLSVV
Chinese hamster	QLGEYGFQNVLLIHYTKKSPQVQTSTLVEIGRKLGSLGSRCCKHPESERMHCAEEYLSLV
Squirrel	QLGEYKFQNALLIRYTQKAPQVSTPTLVEASRNLGRVGTKCCKLPESHRMPCVEDYLTAI
Golden hamster	KLGEYGFQNALIVRYTQKAPQVSTPTLVEAARNLGKVGSKCCVLPEAQRLPCVEDYISAI
Mouse	KLGEYGFQNAILVRYTQKAPQVSTPTLVEAARNLGRVGTKCCTLPEDQRLPCVEDYLSAI
Rat	KLGEYGFQNAILVRYTQKAPQVSTPTLVEAARNLGRVGTKCCTLPEAQRLPCVEDYLSAI
Naked mole rat	KLGEYGFQNALVVRYTQKIPQISTATLVEASRNLGKVGTKCCALPESKRLPCTENYLALI
Guinea pig	KLGEYGFQNALAVRYTQKAPQVSTPTLVEYARKLGSVGTKCCSLPETERLSCTENYLALI
Rabbit	QLGDYNFQNALLVRYTKKVPQVSTPTLVEISRSLGKVGSKCCKHPEAERLPCVEDYLSVV
Pika	ELGEYNFQNLLIVRYTKKAPQVSTPTLVEIARGLGKVGSKCCKLGEAERFRCVEDYVSVV
Pig	KLGEYGFQNALIVRYTKKVPQVSTPTLVEVARKLGLVGSRCCKRPEEERLSCAEDYLSLV
Alpaca	KLGEYGFQNDILVRYTKKLPQVSTPTLVEVARGLGRVGTKCCTLPESNRMSCAEDYLSLV
Bactrian camel	KLGEYGFQNDILVRYTKRLPQVSTPTLVEVARGLGRVGTKCCTLPESNRMSCAEDYLSLI
Dolphin	KLGEYQFQNALIVRYTKKVPQVSTPTLVEVSRNLGRVGSKCCKNPESERMSCAEDYLSLV
Killer whale	KLGEYQFQNALIVRYTKKVPQVSTPTLVEVSRNLGRVGSKCCKNPESERMSCAEDYLSLV
Cow	KLGEYGFQNALIVRYTRKVPQVSTPTLVEVSRSLGKVGTRCCTKPESERMPCTEDYLSLI
Sheep	KHGEYGFQNALIVRYTRKAPQVSTPTLVEISRSLGKVGTKCCAKPESERMPCTEDYLSLI
Goat	KHGEYGFQNALIVRYTRKAPQVSTPTLVEISRSLGKVGTKCCAKPESERMPCTEDYLSLI
Horse	EVGEYDFQNALIVRYTKKAPQVSTPTLVEIGRTLGKVGSRCCKLPESERLPCSENHLALA
White rhinoceros	RLGEYDFQNELIVRYTHKVPQASTPTLVEISRKMGKVGTKCCKVSEPQRLPCSENYLSLI
Cat	KLGEYGFQNALLVRYTKKVPQVSTPTLVEVSRSLGKVGSKCCTHPEAERLSCAEDYLSVV
Dog	KLGEYGFQNALLVRYTKKAPQVSTPTLVEVSRKLGKVGTKCCKKPESERMSCADDFLSVV
Ferret	KLGEYGFQNALLVRYTKKLPQVSTPTLVEVSRKLGKVGTKCCTKPETERMSCAEDYLAVV
Panda	KLGEYGFQNALLVRYTKKVPQVSTPTLVEVSRKLGKVGTKCCKKPESERMSCAEDYLSVV
Pacific walrus	KLGEYGFQNALLVRYTKKVPQVSTPTLVEVSRKLGKVGTRCCKKPESERMPCAEDYLSVV
Weddell seal	KLGEYGFQNALLVRYTKKVPQVSTPTLVEVSRKLGKVGTRCCKKPDSERMPCAEDYLSVV
Black flying fox	NLGAYGFQNALIIRYTKKMPQVSTPTLLVISKELANMGNKCCTLPESKRMACAEDYLSLV
Megabat	NLGPYGFQNMLTIRYTKKMPQVSTPTLLVISKELANMGNKCCTLPESKRMACAEDYLSLV
Hedgehog	TVGEYQFQNALLVRYTKKIPQVSTPTLVDTVRKLGRVGTRCCGLPESERLLCAVDYLSLV
Shrew	NLQEYGFQNALLIRYTKKMPQVSTPTLVEVSRNLGKVGTKCCSLAEGERLPCAEDYLSVV
Star nosed mole	NLGEYGFQNALIVRYTKKMPQVSTPSIVEVSRSLGKVGTKCCTRPESNRMACAEDFLSVV
Hyrax	KLGEYDFTNSLLVRYTRKTPQVSTPTLVKVARKMGKVGSKCCKLADSQRVACAEDY----
Elephant	QLGEYGFTNELVVRYTRKVPQVSTPTLVEVSRKLGRVGSKCCKLAEAKRMPCAEDYLSLV
Manatee	HLGEYGFTNKLLVRYTRKVPQVSTPTLVEVSRKLGRVGTKCCKLAESERMPCADDFLSVI
Cape golden mole	QLGEYGFQNALVVRYTRKLPQVSTPTLVEVSRNLGRVGTKCCKLAETERMSCAEDYLSLV
Tenrec	KIGEYGFQNALIIRYTQKVPQVSTPTLVETSRGLGRVGTKCCSLPEAQKMPCVEDHLSVI
Armadillo	QLGEYGFTNLLAIRYTHKFPEVSTPTLVEISRKLGRTGSACCKRPEAERMSCVEEYLTLI
Sloth	QHGDYGFTNLLLIRYTKKFPEVSAPTLVDISRRLGKTGTWCCKKPESERLSCVEEYLNLI
Opossum	KAGEYGFANELVVRYTKKMPQVSTPTVVELSQKLAKVGSKCCKLSDDEKMGCAEGYLAIV
Tasmanian devil	KVGEYGFENALLVRYTRKAPQVSTPTLLALGHKLAKVGSKCCKVSDNEKLGCAEGYLAIV
Platypus	KLGEYAFQNTVLVRYTRKLPQVSTPTLLDVSSHLARVGTRCCKLDEGHRLACADDYLALV

Human	LNQLCVLHEKTPVSDRVTKCCTESLVNRRPCFSALEVDETYVPKEFNAETFTFHADICTL
Chimpanzee	LNQLCVLHEKTPVSDRVTKCCTESLVNRRPCFSALEVDETYVPKEFNAETFTFHADICTL
Bonobo	LNQLCVLHEKTPVSDRVTKCCTESLVNRRPCFSALEVDETYVPKEFNAETFTFHADICTL
Gorilla	LNQLCVLHEKTPVSDRVTKCCTESLVNRRPCFSALEVDETYVPKEFNAETFTFHADICTL
Orangutan	LNQLCVLHEKTPVSERVTKCCTESLVNRRPCFSALEVDETYVPKEFNADTFTFHADICTL
Gibbon	LNRLCVLHEKTPVSDRVTKCCTESLVNRRPCFSALEVDETYVPKEFNAETFTFHADICTL
Rhesus	LNRLCVLHEKTPVSEKVTKCCTESLVNRRPCFSALELDEAYVPKAFNAETFTFHADMCTL
Crab-eating macaque	LNRLCVLHEKTPVSEKVTKCCTESLVNRRPCFSALELDEAYVPKAFNAETFTFHADMCTL

Olive baboon	LNRLCVLHEKTPVSEKVTKCCTESLVNRRPCFSALELDEAYVPKAFNAETFTFHADMCTL
Marmoset	LNQLCVLHEKTPVSDRVTKCCTESLVNRRPCFSALEVDETYLPKAFNAETFTFHADMCTL
Squirrel monkey	LNQLCVLHEKTPVSDRVTKCCTESLVNRRPCFSALTVDETYVPKAFNAETFTFHADMCTL
Tarsier	--
Mouse lemur	LNRLCVLHEKTPVSDRVTKCCTESLADRRPCFSELEADETYVPKPFTAETFTFHADVCTL
Bushbaby	LNRLCVLHEKTPVSDRVTKCCTESLVNRRPCFSALEVDETYVPKEFHAETFTFHADMCTL
Chinese hamster	LNKMCLAHEKAPVSDRITKCCSESLVNRRPCFSALPPDDTFEPKKFEADSFTFHADICTI
Squirrel	LNTVCVMHEKNPVSERITKCCSESFVNKRACFSALSVDDTYVPKEFHADTFTFHADICTL
Golden hamster	LNRVCVLHEKTPVSEQVTKCCTGSVVERRPCFSALPVDETYVPKEFKAETFTFHADICSL
Mouse	LNRVCLLHEKTPVSEHVTKCCSGSLVERRPCFSALTVDETYVPKEFKAETFTFHSDICTL
Rat	LNRLCVLHEKTPVSEKVTKCCSGSLVERRPCFSALTVDETYVPKEFKAETFTFHSDICTL
Naked mole rat	LNRLCVLHEKTPVSDRVTKCCTESLVNRRPCFSALQLDETYVPKQFTDETFTIHADVCTL
Guinea pig	LNRLCILHEKTPVSERVTKCCTESLVNRRPCFSALHVDETYVPKPFHADSFTFHADICTL
Rabbit	LNRLCVLHEKTPVSEKVTKCCSESLVDRRPCFSALGPDETYVPKEFNAETFTFHADICTL
Pika	LNRFCVLHEKTPVSEGVTKCCTESLVDRRACFSDLTPDSTYVPKEFHVETFTFHADICTL
Pig	LNRLCVLHEKTPVSEKVTKCCTESLVNRRPCFSALTPDETYKPKEFVEGTFTFHADLCTL
Alpaca	LNRLCVLHEKTPVSPRVTKCCTESLVNRRPCFSSLAADETYEPKEFDDKTFTFHADLCSV
Bactrian camel	LNRLCVLHEKTPVSPRVTKCCTESLVNRRPCFSSLTADETYEPKEFDEKTFTFHADLCSV
Dolphin	LNRLCVLHEKTPVSERVTKCCTESLVNRRPCFSALTVDETYEPKAFDEKTFTFHADLCTL
Killer whale	LNRLCVLHEKTPVSERVTKCCTESLVNRRPCFSALTVDETYEPKAFDEKTFTFHADLCTL
Cow	LNRLCVLHEKTPVSEKVTKCCTESLVNRRPCFSALTPDETYVPKAFDEKLFTFHADICTL
Sheep	LNRLCVLHEKTPVSEKVTKCCTESLVNRRPCFSDLTLDETYVPKPFDEKFFTFHADICTL
Goat	LNRLCVLHEKTPVSEKVTKCCTESLVNRRPCFSDLTLDETYVPKPFDGESFTFHADICTL
Horse	LNRLCVLHEKTPVSEKITKCCTDSLAERRPCFSALELDEGYVPKEFKAETFTFHADICTL
White rhinoceros	LNRLCVLHEKTPVSERVTKCCTDSLVNRRPCFSALQLDETYIPKEFNAETFTFHADICTL
Cat	LNRLCVLHEKTPVSERVTKCCTESLVNRRPCF-ALQVDETYVPKEFSAETFTFHADLCTL
Dog	LNRLCVLHEKTPVSERVTKCCSESLVNRRPCFSGLEVDETYVPKEFNAETFTFHADLCTL
Ferret	LNRLCVLHEKTPVSDRVTKCCSESLVNRRPCFSALAGDETYVPKEFNAETFTFHADLCTL
Panda	LNRLCVLHEKTPVSERVTKCCTESLVNRRPCFSALEVDETYVPKEFNAETFTFHADLCTL
Pacific walrus	LNRLCVLHEKTPVSERVTKCCTESLVNRRPCFSALEVDEAYVPKEFNAETFTFHADLCTL
Weddell seal	LNRLCVLHEKTPVSERVTKCCTESLVNRRPCFSALEIDEAYVPKEFNAETFTFHADLCTL
Black flying fox	LNRLCVLHEKTPVSDKITKCCTDSLVNRRPCFSSLEADETYVPKEFNAETFTFHADVCTL
Megabat	LNRLCVLHEKTPVSDKITKCCTDSLVNRRPCFSSLEADETYVPKEFNAETFTFHADVCTL
Hedgehog	LNHLCVLHEKTPVSDKVTKCCSESLVNRRPCFSALEADETYVPKDFSAETFTFHADICTL
Shrew	LNRLCVLHDKTPVSDKVTKCCSESLVNRRPCFSALEADETYVPKDFNAEMFTFHADICTL
Star nosed mole	LNRLCVLHEKTPVSEKVTKCCTESLVNRRPCFSALQLDDTYKPKEFNAETFTFHADICTL
Hyrax	-----------PVSDKITKCCTESL-NRRPCFTALE-DDTYVPKPFSAETFTFHADLCTL
Elephant	LNRLCVLHEKTPVSERITKCCTESLVNRRPCFTALQTDETYVPKEFNAETFTFHADLCTL
Manatee	LNRLCVLHEKTPVSERITKCCTESLVNRRPCFTALHADETYVPKEFNDETFTFHADLCTL
Cape golden mole	LNRLCVLHEKTPVSEKVTTCCSNSLVYRRPCFSALEVDETYAPKEFSAETFTFHSDLCTL
Tenrec	LNWLCVLHEKTPVSERVTKCCTESLVNRRPCFSALEVDETYVPKEFIPETFTFHAELCTL
Armadillo	LNHLCVLHEKTPVSEVVTKCCSESLVNIRPCFSALPPNEAYVPKEFSA-TFTFHAELCTL
Sloth	LNRLCVLHEKTPVSEQVTKCCTESLVNRRPCFSALPPKEDYVPKEFNLATFTFHADICEL
Opossum	VDKLCRQHEKTPVSDKITKCCTESLVNRRPCFTALGVDETYEPKAFSADTFTFHADLCTL
Tasmanian devil	IDNLCRLHEKTPVSDRITKCCKDSLVNRRPCLSALGVDDTYVAKPFSADTFTFHADLCTL
Platypus	LDKMCRLHEKTPVSDRVTKCCTDSFADRRPCFSALGVDETFVPKEFNADTFTFHADLCTL

Human	SEKERQIKKQTALVELVKHKPKATKEQLKAVMDDFAAFVEKCCKADDKETCFAEEGKKLV
Chimpanzee	SEKERQIKKQTALVELVKHKPKATKEQLKAVMDDFAAFVEKCCKADDKETCFAEEGKKLV
Bonobo	SEKERQIKKQTALVELVKHKPKATKEQLKAVMDDFAAFVEKCCKADDKETCFAEEGKKLV
Gorilla	SEKERQIKKQTALAELVKHKPKATKEQLKTVMDDFAAFVEKCCKADDKETCFAEEGKKLV
Orangutan	SEKERQIKKQTALVELVKHKPKATKEQLKTVMEDFAAFVEKCCKADDKETCFAEEGKKLV
Gibbon	SEKDRQVKKQTALVELVKHKPKATKEQLKTVMEDFAAFVEKCCKADDKETCFAEEGKKLV
Rhesus	SEKEKQVKKQTALVELVKHKPKATKEQLKGVMDNFAAFVEKCCKADDKEACFAEEGPKFV
Crab-eating macaque	SEKEKQVKKQTALVELVKHKPKATKEQLKGVMDNFAAFVEKCCKADDKEACFAEEGPKFV
Olive baboon	SEKEKQVKKQTALVELVKHKPKATKEQLKGVMDNFAAFVEKCCKADDKEACFAEEGPKFV
Marmoset	SEKEQQVKKQTALAELVKHKPKATNEQLKTVMEDFAAFVEKCCKADDKEACFAEEGPKLV
Squirrel monkey	SEKAQQVKKQTALVELVKHKPQATKEQLKTVMENFAAFVEKCCKADDKEACFAAEGPKLV
Tarsier	-----------ALVELVKHKPKATEEQLKDVMGDFTVFVDKCCKAENKEACFAEEGKTCG
Mouse lemur	PEKEKQIKKQTALVELVKHKPKATDEQLKAVMTDFTTFVETCCKADDKEGCFSEEGPTCC
Bushbaby	PDKEKQLKKQTALVELVKHKPKATDEQLKAVMGKFTDFVEKCCKADDKEACFAEEGPKLV
Chinese hamster	PDTEKQIKKQTALVELVKHKPHATNDQLKTVMGDFTAFVDKCCKAEDKEACFAEEGPTCC
Squirrel	PETEQQIKKQTALAELVKHKPTATIDQLKTVMGDFVAFLDKCCKADDKDTCFSQEGPKFV
Golden hamster	PEKEKQMKKQAALVELVKHKPKATGPQLRTVLGEFTAFLDKCCKAEDKEACFSEDGPKLV
Mouse	PEKEKQIKKQTALAELVKHKPKATAEQLKTVMDDFAQFLDTCCKAADKDTCFSTEGPNLV

```
Rat                     PDKEKQIKKQTALAELVKHKPKATEDQLKTVMGDFAQFVDKCCKAADKDNCFATEGPNLV
Naked mole rat          PEEEQQIMKQMTLAELVKHKPKATAEQMKDIMKNFGIFLEKCCHAENKDDCFSKEGSNFV
Guinea pig              PEKEKQVKKQMALVELVKHKPKASEEQMKTVMGDFAAFLKKCCDADNKEACFTEDGPKLV
Rabbit                  PETERKIKKQTALVELVKHKPHATNDQLKTVVGEFTALLDKCCSAEDKEACFAVEGPKLV
Pika                    PQHKREYKKQKALVELVKYKPSATKEQLKKVVGDFTAMVEKCCAADDKEACFSEEWKTCV
Pig                     PEDEKQIKKQTALVELLKHKPHATEEQLRTVLGNFAAFVQKCCAAPDHEACFAVEGPKFV
Alpaca                  SEPEKQIKKQ-ALAELLKHKPKATDEQLKTVMEKFVAFVEKCCAAVDKEGCFSVEGQTSC
Bactrian camel          SEPEKQIKKQTALAELLKHKPKATDEQLKTVMEKFVAFVDKCCAAVDKEACFTVEGPLLV
Dolphin                 PENEKQIKKQIALVELVKHKPKVTEEQLKTVMGDFAAFVDKCCAADDKEACFALEGPKLV
Killer whale            PENEKQIKKQIALVELVKHKPKVTEEQLKTVMGDFAAFVDKCCAADDKEACFALEGPKLV
Cow                     PDTEKQIKKQTALVELLKHKPKATEEQLKTVMENFVAFVDKCCAADDKEACFAVEGPKLV
Sheep                   PDTEKQIKKQTALVELLKHKPKATDEQLKTVMENFVAFVDKCCAADDKEGCFVLEGPKLV
Goat                    PDTEKQIKKQTALVELLKHKPKATDEQLKTVMENFVAFVDKCCAADDKEGCFLLEGPKLV
Horse                   PEDEKQIKKQSALAELVKHKPKATKEQLKTVLGNFSAFVAKCCGAEDKEACFAEEGPKLV
White rhinoceros        PDDEKKIKKQTALVELVKHKPKATNEQLKAVIEDFAAFINKCCAAENKEACFAEEGPKLV
Cat                     PEAEKQIKKQSALVELLKHKPKATEEQLKTVMGDFGSFVDKCCAAEDKEACFAEEGPKLV
Dog                     PEAEKQVKKQTALVELLKHKPKATDEQLKTVMGDFGAFVEKCCAAENKEGCFSEEGPKLV
Ferret                  PDAEKQVKKQSALVELLKHKPKATEEQLKTVMGDFGAFVDKCCAAENKEGCFAEEGPKLV
Panda                   PEAEKQVKKQSALVELLKHKPKATEEQLKTVMGDFGAFVDKCCAAENKEGCFAEEGPKLV
Pacific walrus          PEAEKQIKKQSALVELVKHKPKATEEQLKTVMGNFGAFVEKCCAAEEKEACFAEEGPKLV
Weddell seal            PEAEKQVKKQSALAELVKHKPKATEEQLKTVMGDFGAFVEKCCAAENKEACFAEEGPKLV
Black flying fox        PDHEKHLKTQTALVELLKHKPKAADEQLKTVMGNFSAFIEKCCTADDKEACFAEEGPKLV
Megabat                 PDSEKHLKEQTALVELLKHKPKATDEQLKTVMGNFSAFIEKCCTADDKEACFAEEGPKLV
Hedgehog                PDTEKEIKKQTTLVELLKHKPKATDAQLKTVMGDFTAFLTKCCAEADKEACFATEGPTCC
Shrew                   PDSEKQFKKQAALVELLKHKPKATEEQLKPVMAEFAAFVEKCCGAEDKEACFATEGPKLI
Star nosed mole         PEAEKQIKKQSALVELLKHKPQATGDQLKTVMGDFGGFIEKCCASDDKETCFSTEGPKLV
Hyrax                   PDDQKQSKKQTVLVELLKHKPKATDEQLKKVIGLFKAVVEKCCAAEDKEACFGVEAKTCY
Elephant                PEDQKQIKKQSVLVELVKHKPKATDEQLKTVTGQFTGMVEKCCAAEDKEACFAEEGPKLV
Manatee                 PEDQKQIKKQTVLVELLKHKPKATDDKLKTVIGEFTAMVEKCCKADSPEACFTLEGPKLV
Cape golden mole        PEDQKQTKKQTVLVELVKHKPKATDDQLKKVAGEFSAMVEKCCKVEDHEVCFAEEGPKLV
Tenrec                  PDDQKQIKKQSVLAELVKHKPTATEDALKAVSGEFVIMMEKCCTDADQEGCFGREGPTCG
Armadillo               P-DE--I----TLVELVKHKPKATYDQIKVVMTDFTAMVEKCCKAENKEACFAEEGPKLV
Sloth                   SEEEKQIKKQVVLVEVLKHKPKATDEQLKTVKEKVTGFLDKCCHAENKEACFIAEGQNIH
Opossum                 PEEEKQTKKQTVLAELVKHKPKITQDQLKAVISDFTAFVDKCCKADSQEGCFAEDGPKLV
Tasmanian devil         PEEEKQDKKQGILVELVKHKPKITDDQLKGVITDFTAFVDKCCKADNQEACFAEDGPKLV
Platypus                PEDQQKAKKQSVLVELVKHKPKATDDQLKGIVTDFTAMVTKCCGEADKEACFATEGANLV


Human                   AASQAAL-
Chimpanzee              AASQAAL-
Bonobo                  AASQAAL-
Gorilla                 AASQAAL-
Orangutan               AASQAAL-
Gibbon                  AASQAAL-
Rhesus                  AASQAAL-
Crab-eating macaque     AASQAAL-
Olive baboon            AASQAAL-
Marmoset                AASETAL-
Squirrel monkey         AESQAAL-
Tarsier                 KASQPSG-
Mouse lemur             KAYQPTL-
Bushbaby                AESQAAL-
Chinese hamster         CATQPAL-
Squirrel                AASQAAL-
Golden hamster          ASSQAAL-
Mouse                   TRCKDAL-
Rat                     ARSKEAL-
Naked mole rat          QACKDTF-
Guinea pig              AKCQATL-
Rabbit                  ESSKATL-
Pika                    KQSQLTP-
Pig                     IEIRGIL-
Alpaca                  CASQPTL-
Bactrian camel          AATRTALA
Dolphin                 VKTREAI-
Killer whale            VTTREAI-
```

Cow	VSTQTAL-
Sheep	ASTQAAL-
Goat	ASTQAAL-
Horse	ASSQLAL-
White rhinoceros	AKSQTALL
Cat	AAAQAAL-
Dog	AAAQAAL-
Ferret	AAAQAAL-
Panda	ATAQAAL-
Pacific walrus	ATAQAAL-
Weddell seal	AKAQAALA
Black flying fox	ASSQATLA
Megabat	ASSQATLA
Hedgehog	YVSQPSL-
Shrew	ASSQAALA
Star nosed mole	AAAQAAL-
Hyrax	KASQPTL-
Elephant	TASQAAL-
Manatee	ATSKAAL-
Cape golden mole	AAAQATLA
Tenrec	SVFQPIM-
Armadillo	KEAEPTL-
Sloth	NQASPTL-
Opossum	ASAQAAL-
Tasmanian devil	ATTQAAL-
Platypus	TKSKAALA

VITAMIN D-BINDING PROTEIN (GC)

Human	MKRVLVLLLAVAFGHALERGRDYEKNKVCKEFSHLGKEDFTSLSLVLYSRKFPSGTFEQV
Chimpanzee	MKRVLVLLLAVAFGHALERGRDYEKNKVCKEFSHLGKEDFTSLSLVLYSRKFPSGTFEQV
Bonobo	MKRVLVLLLAVAFGHALERGRDYEKNKVCKEFSHLGKEDFTSLSLVLYSRKFPSGTFEQV
Gorilla	MKRVLVLLLAVAFGHALERGRDYEKNKVCKEFSHLGKEDFTSLSLVLYSRKFPSGTFEQV
Orangutan	MKRVLVLLLAVAFGHALERGRDYEKNKVCKEFSHLGKEDFTSLSLVLYSRKFPSGTFEQV
Gibbon	MKRVLVLLLAVAFGHALERGRDYEKNKVCKEFSHLGKEDFTSLSLVLYSRKFPSGTFEQV
Rhesus	MKRVLVLLLAVAFGHALERGRDYEKNKVCKEFTHLGKEDFTSLSLVLYSRKFPSGTFEQV
Crab-eating macaque	MKRVLVLLLAVAFGHALERGRDYEKNKVCKEFTHLGKEDFTSLSLVLYSRKFPSGTFEQV
Olive baboon	MKRVLVLLLAVAFGHALERGRDYEKNKVCKEFTHLGKEDFTSLSLVLYSRKFPSGTFEQV
Marmoset	MKKALVLLLAVAFGHALERGRDYEKDKVCKEFSDLGKGDFTSLSLVLYSRKFPSGTFEQI
Squirrel monkey	MKKVLVLLLAVAFGHALERGRDYEKDKVCKEFSDLGKEDFTSLSLVLYSRKFPSGTFEQI
Tarsier	MKRVLVLLFAVAFGHALERGRDYEKDKVCKEFTNLGKDDFTSLSLVQYSRKFPSGTFEQV
Mouse lemur	--------------------RDYEKDKVCKEFANLGKDDFTSLSLVLYSR-FLS-TFEQV
Bushbaby	-------------------GRDYEKDKVCKEFAHLGKDEFTSLSLVLYSRKFPSGTFEQV
Chinese tree shrew	MKGVLVLLLAAALGHALERGRDYEKEKVCKEFISLGKEDFTTLSLVLYSRKFPSGTFEQI
Squirrel	MKRVLVFLLAVAFGHALERGRDYEKEKVCQELASLGKDDFRSLSLVLYSRKFSSGTFEQV
Ground squirrel	MKRVLVFLLAVAFGHALERGRDYEKEKVCQELASLGKDDFRSLSLVLYSRKFSSGTFEQV
Golden hamster	MKRVVALLLVLALGHALERGRDYEKDKVCKELAMLGKDDFRSLSLILYSRKFPSGTFEQV
Kangaroo rat	--------------------RDYEKDKVCKELAMLGKEDFRSLALVLYSRKFSRGTFEQV
Mouse	MKRVLVLLLALAFGHALERGRDYEKDKVCNELAMLGKEDFRSLSLILYSRKFSSSTFEQV
Rat	MKRVLVLLLALAFGHALERGRDYEKDKVCQELSTLGKDDFRSLSLILYSRKFPSSTFEQV
Naked mole rat	MKTVLVLLLTVALGHALERGRDYERDKVCKELTQLGKDDFRTLSLVLYSRKFSSGTFEQV
Guinea pig	MES-LLLCLPVLLCLKLKLGRDFERDKVCKELTNLGKDEFRTLSLILYSRKFSRGTFEQV
Rabbit	MKRVLLLLLAVVCGHALERGRDYEKDKVCKELATLGKDDFRTLSLVLYSRKFPSGTFDQV
Pika	MK-ILVLLLALAYGHALERGRDYEKDKVCKELSALGKDDFRSLSLVLYSRKFPSGTFEQV
Pig	--------------------RDYEKDKVCKELASLGKDDFTSLSMVLYSRKFPSGTFEQI
Alpaca	MKRILVFLLAVACVRALERGQDYEKDKVCKELASLGKDDFTSLSMVLYSRKFPSGTFEQV
Bactrian camel	MAKGLSCLLSAVLFSPLQRGRDYEKDKVCKELASLGKDDFTSLSMVLYSRKFPSGTFEQI
Dolphin	MKRILVFLLAVAFVHALERGRDYEKDKVCKELASLGKDDFTSLSMVLYSRKFPSATFEQI
Killer whale	MKRILVFLLAVAFVHALERGRDYEKDKVCKELASLGKDDFTSLSMVLYSRKFPSATFEQI
Cow	MKRILVFLLAVAFVHALERGRDYEKDKVCKDLASLGRDDFTSLSMVLYSRKFPSGTFEQI
Sheep	MKRILVFLLAVAFVHALERGRDYEKDKVCKELASLGKEDFTSLSMVLYSRKFPSGTFEQI
Goat	MKRILVFLLAVAFVHALERGRDYEKDKVCKELASLGKEDFTSLSMVLYSRKFPSGTFEQI
Horse	MKRIPVVLLAVAFVHALERGRDYEKDKVCKEFTSLGKDDFTSLSMVLYSRKFPSGTFEQI
White rhinoceros	MKKILAVLLAVAFVHTLERGRDYEKDKVCKELASLGKDDFATLSMVLYSRKFPSGTFEQI
Cat	MKRILVLLLAVAFVHALERGRDYEKDKVCKELANLGKDDFTSLSMVSYSRKFPSSTFEQI

Dog	MKTNIFVLLSIIIYSRPQKGRDYEKEKVCKELANLGKDDFTSLSMVLYSRKFPSSTFEQI
Ferret	MKGILVLLLAVAWVHALERGRDYEKDKVCKELANLGKDDFASLSMVLYSRKFPSGTFEQI
Panda	MKRTLVLLLAVAFVHALERGRDYEKDKVCKELANLGKEDFTSLSMVLYSRKFPSSTFEQI
Pacific walrus	MKVILVLLLAVAFVHALERGRDYEKDKVCKELASLGKEDFTSLSMVLYSRKFPSGTFEQI
Weddell seal	MKVILVLLLAVAFVHALERGRDYEKDKVCKELASLGKEDFTSLSMVLYSRKFPSGTFEQI
Megabat	-----------------SAGRDYEKDKVCKELSNLGKDDFTSLSMVLYSRKFPSGTFEQV
David's myotis	MKRILFLLLTV---HALERGREYEKDKVCKELNNFGKDDFTSLSMVLYSRKFPSGTFEQI
Microbat	MKRILFLLLTV---HALERGREYEKDKVCKELINFGKDDFTALSMVLYSRKFPSGTFEQI
Hedgehog	--------------------KDYEKDKVCKELAQMGKDDFRSLSIILYSRKFPSGTFEQI
Shrew	MKTALILLLALAGVHALERGREYEKEKVCKELAHLGKDGFRS------NGEFSFGTFEQI
Star nosed mole	MQRSRHKNVEMMKGTSPDRCRDYEKDKVCKELSILGKDDFRSLSMVLYSRKFPGGTFEQV
Elephant	-------------------GRDYEKDKVCKELSNLGKDDFRSLSMVLYSRKFPSGTFEQV
Hyrax	-----------------------------------GKEDFRSLSMVLYSRKFPSGTFEQV
Cape golden mole	--------------------RDYEKDKVCKELSNLGKDDFRSLSMVLYSRKFPSGTFEQV
Tenrec	MKGVLGLLLMVSFVHALERGRDYEKSKVCAELSNLGKDDFRSLSMISYSRKFASGTFEQV
Armadillo	MKGILFLLFAVGFGHAIERGRDYEKSKVCKELADLGKDDFRFLSMVLYSRKFPSGTFQQV
Sloth	---SMVLYSRKFPSGTFEQV
Tasmanian devil	MSRIMLLLLSVTFIQADYRGRDYEKSKVCKEFANLGKDDFRSLSFVLYSRKFPSGTFEQV
Platypus	MRGFPILLLTLALAQALHRGRDYEKEKVCREFASLGKDDFRSLSIVMHSKKYPRASFSEV

Human	SQLVKEVVSLTEACCAEGADPDCYDTRTSALSAKSCESNSPFPVHPGTAECCTKEGLERK
Chimpanzee	SQLVKEVVSLTEACCAEGADPDCYDTRTSALSAKSCESNSPFPVHPGTAECCTKEGLERK
Bonobo	SLLVKEVVSLTEACCAEGADPDCYDTRTSALSAKSCESNSPFPVHPGTAECCTKEGLERK
Gorilla	SQLVKEVVSLTEACCAEGADPDCYDTRTSALSAKSCESNSPFPVHPGTAECCTKEGLERK
Orangutan	SQLVKEVVSLTEACCVEGADPDCYDTRTSALSAKSCESKSPFPVHPGTAECCTKEGLERK
Gibbon	SQLVKEVVSLTEACCVEGADPDCYDTRTSALSAKSCESNSPFPVHQGTAECCTKEGLERK
Rhesus	SQLVKEVVSLTEACCAEGADPDCYDTRTSALSAKSCESNSPFPVHPGTAECCTTEGLERK
Crab-eating macaque	SQLVKEVVSLTEACCAEGADPDCYDTRTSALSAKSCESNSPFPVHPGTAECCTTEGLERK
Olive baboon	SQLVKEVVSLTEACCAEGADPDCYDTRTSALSAKSCESNSPFPVHPGTAECCTREGLERK
Marmoset	TKLVEEVVSLTETCCAEGADPDCYDTRTSALSAKSCERNSPFPVHPGTAECCTKEGLERK
Squirrel monkey	TKLVEEVVSLTETCCAEGADPDCYDTRTSALSAKSCESNSPFPVHPGTAECCTKEGLERK
Tarsier	SQLVNEVVSLTETCCANWADPDCYETQTSALSAKSGENDSPFPVHPGNVECCTKEDQEKK
Mouse lemur	SQLV-EVVFLTEACCAEA--DDCYDS------------------------------LERK
Bushbaby	KQLVTEVVSLTEACCAEGANPDCYDNRTSALSARSCERDSPFPAHPGTAECCAREGLERK
Chinese tree shrew	SQLVKEVVSLTETCCAEGADPNCYDTRTSALSARSCESDSPFPVHPGTPECCTKEGLEQK
Squirrel	SQLVKEVVSLTEDCCAEGADPDCYDTRTSALSAKSCESDSPFPVHSGTPECCTKEGLERK
Ground squirrel	SQLVKEVVSLTEDCCAEGADPDCYDTRTSALSAKSCESDSPFPVHSGTPECCTKEGLERK
Golden hamster	SQLVKEVVTLTEECCAEGADPNCYDTRTSELSVKSCASDAPFPVHPGTPECCTQEGLERK
Kangaroo rat	SQLVKDVVSLTEECCAKGADPDCYDTRTSELSVKSCETDSPFPVHPGTSECCSKEGLERK
Mouse	NQLVKEVVSLTEECCAEGADPTCYDTRTSELSVKSCESDAPFPVHPGTPECCTKEGLERK
Rat	SQLVKEVVSLTEECCAEGADPNCYDTRTSELSIKSCESDAPFPVHPGTSECCTKEGLERK
Naked mole rat	SQLVQEVVALTEECCAEGADPNCYDTRTSALSAKSCESDSPFPVHKGTPECCTKEGLERK
Guinea pig	SQLVQEVVALTEECCAEGAAPDCYDSRTAALSAKSCESDSPFPVHEGTPACCAAEGLERK
Rabbit	MKLVKEVVSLTEDCCAEDADPGCYDNRTSALSATSCESDSPFPVHPGTAECCTKEGLERK
Pika	TQLVKQVVSLTEDCCAEDADPNCYDDRTTALSVLSCASDSPFPEHPGTAECCTREGLERK
Pig	SHLVNEVVSLTEACCAEGADPDCYDNRTSALSDKSCESDSPFPVHPGTAECCTKEGLEKK
Alpaca	SHLVSEVVSLTEACCTEEADPDCYDNRTSALSAKSCDSDSPFPVHPGTAECCTKEGLERK
Bactrian camel	SHLVNEVVSLTEACCTEEADPDCYDNRTSALSAKSCESDSPFPVHPGTAECCTTEGLERK
Dolphin	SHLVNEVVSLTEACCAEGADPDCYDNRTSALSAKSCESDSPFPVHPGTPECCTKEGLEKK
Killer whale	SHLVNEVVSLTEACCAEGADPDCYDNRTSALSAKSCESDSPFPVHPGTPECCTKEGLEKK
Cow	SHLVNEVVSLTVTCCAEGADPDCYDNRTSALSDKSCESNSPFPVHPGTPECCTHEGLEKK
Sheep	SHLVNEVVSLTITCCAEGADPDCYDNRTSALSDKSCESNSPFPVHPGTPECCTHEGLEKK
Goat	SHLVNEVVSLTITCCAEGADPDCYDNRTSALSDKSCESNSPFPVHPGTPECCTHEGLEKK
Horse	SNLVNEVVSLTEACCAEGADPGCYDNRTSALSVKSCESDSPFPVHPGTPECCTKEGLERK
White rhinoceros	SNLVNEVVSLTEACCAEGANPDCYDNRTSALSVKSCESDSPFPVHPGTAECCTKEGLERK
Cat	SHLVNEVVSLTEACCAEGADPDCYDLRTSALSAKSCESDSPFPVHPGTAECCTKEGLEQK
Dog	SHLVNEVVSLTETCCAEGADPDCYDHRTSALSARSCEKDSPFPVHPGTAECCTREGLERK
Ferret	SQLVKEVVSLTETCCAEGADPDCYDRRTSALSARSCEKDSPFPVHPGTAECCTREGLERK
Panda	SHLVNEVVSLTEACCAEGADPECYDLRTSALSARSCEKDSPFPVHPGTAECCTREGLERK
Pacific walrus	CHLVNEVVSLTEACCAEGADPDCYDRRTSALSARSCEEDSPFPVHPGTAECCTREGLEQK
Weddell seal	CHLVNEVVSLTEACCAEGADPDCYDRRTSALSARSCEKDSPFPVHPGTAECCTREGLEQK
Megabat	SHLVKEVVSLTEACCAEEADPDCYDNRTSALSAKSCESDAPFPVHPGTAECCTQEGLERK
David's myotis	SHLVKEVVSLTEACCAEGADPDCYDNRTSALSAKSCDSDSPFPVHPGTAECCTQEGLERK
Microbat	SHLVKEVVSLTEACCAEGADPDCYDNRTSALSAKSCESDSPFPVHPGTAECCTQEGLERK
Hedgehog	SHLINEVVSLTEACCAEGADSDCYDNRTSALSAKSCESNSPFPVHPGTPECCAQEGLEQK

Shrew	THHIDEVVSMTEDCCAPGSDPGCYDSRTSALSEKSCESDSPFFPVHPGTSECCASEGLERK
Star nosed mole	QQLVNEVVSLTEDCCAEGADPDCYDTRTSALSARSCESDAPFFPVHPGTAECCTREGLEQK
Elephant	SQLVNEVVSLTEDCCAEGADPNCYDTRTSALSIKSCESDSPFFPVHPGTAECCTEEGLERK
Hyrax	SQLVDEVVSLTEECCAEGADPGCYDNRTSALSVKSCESDSPFFPVHPGTAECCTKEGLERK
Cape golden mole	RKLVEEVVSLTEDCCAEEADPQCYDNRTSALSVKSCESDSPFFPVHPGTEECCTKEGLERK
Tenrec	KQLVQAVVSLTEDCCAEGADPNCYDDRTSALSARSCEQGSPFFPVHPGTAECCTKEGLERK
Armadillo	RQLVNEVVSLTEACCADGADPDCYDNRTSALSAKSCERDSPFFPVHPETAECCTREGLEQK
Sloth	SQLVNEVVSLTEACCAEGADPDCYDKRTSALSAKSCESDSPFFPVHPGTAACCTSEGLERK
Tasmanian devil	KALVNEVVSLTETCCDVNADPNCYDTRTSELSAKSCEKDAPYPKHPETDKCCTFEGLERK
Platypus	IALVNEVVSLTETCCAQGADPDCYDEGASALSAKSCEKDSPFFPRHPDVAECCAKGGLERK

Human	LCMAALKHQPQEFPTYVEPTNDEICEAFRKDPKEYANQFMWEYSTNYGQAPLSLLVSYTK
Chimpanzee	LCMAALKHQPQEFPTYVEPTNDEICEAFRKDPKEYANQFMWEYSTNYGQAPLSLLVSYTK
Bonobo	LCMAALKHQPQEFPTYVEPTNDEICEAFRKDPKEYANQFMWEYSTNYGQAPLSLLVSYTK
Gorilla	LCMAALKHQPQEFPTYVEPTNDEICEAFRKDPKEYANQFMWEYSTNYGQAPLSLLVSYTK
Orangutan	LCMAALKHQPQEFPTYVEPTNDEICEAFRKDPKEYANQFMWEYSTNYGQAPLSLLVSYTK
Gibbon	LCMAALKHQPQEFPTYVEPTNDEICEAFRKDPKEYANQFMWEYSTNYGQAPLSLLVSYTK
Rhesus	LCMAALKHQPQEFPTYVEPTNDEICEAFRKDPKEFADKFMWEYSTNYGQAPLSLLVSYTK
Crab-eating macaque	LCMAALKHQPQEFPTYVEPTNDEICEAFRKDPKEFADKFMWEYSTNYGQAPLSLLVSYTK
Olive baboon	LCMAALKHQPQEFPTYVEPTNDEICEAFRKDPKEFADKFMWEYSTNYGQAPLSLLVSYTK
Marmoset	LCMAALKHPPEEFPSYVEPTNDEICEAFRKDPKEFANHFMWEYSTNYGQAPLSLLVSYTK
Squirrel monkey	LCMAALKHPPQEFPTYVEPTNDEICEAFRKDPKEFANHFMWEYSTNYGQAPLSLLVSYTK
Tarsier	LCMVALKHRPQEFPTYVEPTNDEICEAFRKSPKGFADQFMYEYSSNYGQAPLPLLVSYTK
Mouse lemur	LCM-ALKHPPQEFPTYVEPTNEEICEAFRKDPKGFAAQFLYEYSSNYGQAPLPLLVSYTK
Bushbaby	LCMAALKHQPQEFPTYVEPTNDEICEAFRKDPKGFADQFMYEYSSNYGQAPLPLLVSYTK
Chinese tree shrew	LCMAALKHQPQEFPTYVEPTNDEICEAFRKDPKEFADQFIYEYSSNYGQAPLPLLVSYTK
Squirrel	LCMAALKHQPQEFPTYVEPTNDEICEAFRKDPKGFADQFLYEYSSNYGQAPLPLLIGHTK
Ground squirrel	LCMAALKHQPQEFPTYVEPTNDEICEAFRKDPKGFADQFLYEYSSNYGQAPLPLLIGHTK
Golden hamster	LCMAALKHQPQEFPTYVEPTNDEICEAFRKDPKGFADQFLYEYSSNYGQAPLSLLVGYTK
Kangaroo rat	LCMAALHHQPQEFPTYVEPTNEEICEAFRKDPKGFADQFLYEYSSSYGQAPLPLLVSYTK
Mouse	LCMAALSHQPQEFPTYVEPTNDEICEAFRRDPKGFADQFLYEYSSNYGQAPLPLLVAYTK
Rat	LCMAALSHQPQEFPAYVEPTNDEICEAFRKDPKGFADQFLFEYSSNYGQAPLPLLVGYTK
Naked mole rat	LCMAALSHQSREFPTYVEPTNDEICEAFRKDPRGFADQFLYEYSSNYGQVPLPLLVGYTK
Guinea pig	LCMAALSHQPQEFPTYVEPTNEEICEAFKKDPRRFADQFVYEYSSNYGQVPLLLLVSYTK
Rabbit	LCMAALKHPPQEFPTYVEPANDEICEAFRQDPMEFADKFLYEYSSNYGQAPLPILVSYTK
Pika	LCMAALKHPPQEFPTYVEPTNDEICEAFRRDPMEFADKFLYEYSVNYGQAPLPILVSYTK
Pig	LCMAALKHQPQEFPTYVEPTNDEICEAFRKDPKGFANQFMYEYSINYGQAPLTLLVSYTK
Alpaca	LCMAALKHQPQEFPTYVEPTNDEICEAFRKDPKGFANQFLYEYSINYGQAPRQILVSYTK
Bactrian camel	LCMAALKHQPQEFPTYVEPTNDEICEAFRKDPKGFANQFLYDYSINYGQAPLTILVSYTK
Dolphin	LCMAALKHQPQEFPTYVEPTNDEICEAFRKDPKGFADQFMYEYSINYGQAPLTLLVSYTK
Killer whale	LCMAALKHQPQEFPTYVEPTNDEICEAFRKDPKGFADQFMYEYSINYGQAPLTLLVSYTK
Cow	LCMAALKHQPQEFPTYVEPTNDEICEAFRKDPKDFADRFMYEYSINYGQAPLTLLVGYTK
Sheep	LCMAALKHQPQEFPTYVEPTNDEICEAFRNDPKGFADQFMYEYSINYGQAPLTLLVGYTK
Goat	LCMAALKHQPQEFPTYVEPTNDEICEAFRNDPKGFADQFMYEYSINYGQAPLTLLVGYTK
Horse	LCMAALKHQPQEFPTYVEPTNEEICEAFRKDPKGFAEQFMYEYSINYGQAPLPLLVGYTK
White rhinoceros	LCMAALKHQPQEFPTYVEPQNDEICEAFRKDPKGFAEQFIYEYSINYGQAPLPLLVGYTK
Cat	LCMAALKHQPQEFPTYVEPTNDEICEAFRKDPKDFAEQFMYEYSINYGQAPLPLLVGYTK
Dog	LCMAALKHQPQEFPTYVEPTNDEICEAFRKDPKDFAEKFMYEYSINYGQAPLSLLVGYTK
Ferret	LCMATLRHQPQEFPTYVEPTNDEICEAFRKDPKDFAEQFMYEYSINYGQAPLSLLVGYTK
Panda	LCMAALKHKPQEFPTYVEPTNDEICEAFRKDPKDFAEQFMYEYSINYGQAPLSLLVSYTK
Pacific walrus	LCMAALRHQPQEFPTYVEPTNDEICEAFRKDPKDFAEQFMYEYSINYGQAPLSLLVSYTK
Weddell seal	LCMAALRHQPQEFPTYTEPTNDEICEAFRKDPKDFAEQFMYEYSINYGQAPLSLLVSYTK
Megabat	LCMASLKHRPQEFPTYVEPTNDEICEAFRKDPKGFANQFMYDYSINYGQAPLPLLVGYTK
David's myotis	LCMAALKHQPQEFPTYVEPSNDEICEAFRKDPKEFANQFMYEYSINYGQAPLPLLVSYTK
Microbat	LCMAALKHQPQEFPTYVEPSNDEICEAFRKDPKEFANQFMYEYSINYGQAPLPLLVSYTK
Hedgehog	LCMAALKHPPQEFPTYTEPTNDEICEAFRKDPKEFAEHFLYDYSSNYGQAPLPLLVGYTK
Shrew	LCMAALKHQPQEFPTYVELTNDEICEAFRKDPKGFAEQFMYEYSINYGQAPLPLLVRYTK
Star nosed mole	LCMAALKHQPEEFPTYVEPTNDEICEAFRKDPKGFAEQFMYEYSKNYGQAPLLLLGGYIK
Elephant	LCMAVLKHQPQEFPTYVEPTNDEICEAFRKDPKQFADQFIYEYSINYGQAPLPLLVSYVK
Hyrax	LCMAVLKHQPQEFPTYVEPTNDEICEAFRRDPKDFANQFAYEYSINYGQAPLSLLVSYVK
Cape golden mole	LCMATLKHQPQEFPTYVEPTNDEICEAFRKDPKEFADQFIYEYSSNYGQAPLPLLVNYVK
Tenrec	LCMAALKHQPQEFPTYVEPTNDEICEAFRKDPKDFADQFIYEYSSNYGEAPLPLLVSYIK
Armadillo	LCMAALKHQPQEFPTYVEPTNLEICEAFRKDPKEFAERFMYEYSINYGHASLALLVSYTK
Sloth	LCMATLKHQPQEFPTYVEPTNDEICEAFRKDPKEFADQFIYEYTINYG-APVSLLVSYTK
Tasmanian devil	LCMAALTHPPQEFPTYVEPTNNEICDSFRMDPRGFADEFLYEYSSNYGQAPLPLLVSYTR

Platypus	LCMAALMQPPQEFPTYVEPSNDETCEAFKKDPKGFAEQFLYEYSSNYGQAPLRLLLGYTK

Human	SYLSMVGSCCTSASPTVCFLKERLQLKHLSLLTTLSNRVCSQYAAYGEKKSRLSNLIKLA
Chimpanzee	SYLSMVGSCCTSASPTVCFLKERLQLKHLSLLTTLSNRVCSQYAAYGEKKSRLSNLIKLA
Bonobo	SYLSMVGSCCTSASPTVCFLKERLQLKHLSLLTTLSNRVCSQYAAYGEKKSRLSNLIKLA
Gorilla	SYLSMVGSCCTSASPTVCFLKERLQLKHLSLLTTLSNRVCSQYAAYGEKKSRLSNLIKLA
Orangutan	SYLSMVGSCCTSASPTVCFLKERLQLKHLSLLTTLSNRVCSQYAAYGEKKSRLSNLIKLA
Gibbon	SYLSMVGSCCTSASPTVCFLKERLQLKHLSLLTTLSNRVCSQYAAYGEKKSRLSNLIKLA
Rhesus	NYLSMVGSCCTSESPTVCFLKERLQLKYLSLLTTLSNRVCSQYAAYGEKKSRLSNLIKLA
Crab-eating macaque	NYLSMVGSCCTSESPTVCFLKERLQLKYLSLLTTLSNRVCSQYAAYGEKKSRLSNLIKLA
Olive baboon	NYLSMVGSCCTSENPTVCFLKERLQLKYLSLLTTLSNRVCSQYAAYGEKKSRLSNLIKLA
Marmoset	SYLSMVGSCCISANPTTCFLNERLQIKHLSLLTTMSNRVCSQYAAYGEKKSRLSNLIKLA
Squirrel monkey	SYLSMVGSCCISANPTACFLNERLQIKHLSLLTTMSNRVCSQYAAYGEKKSRLSNLIKLS
Tarsier	SYLSMVGSCCTSTSPTVCFLKERLQMKHLSLLTTMSNRVCSQYVAYGKEKSRLSQLIKLA
Mouse lemur	SYLSMVGSCCTSASPTVCFLKERLQSKHLSLLTTLSNRVCSQYAAYGKEKSRLSHLIKLA
Bushbaby	SYLSMVGSCCTSPSPTVCFLKERLQMKPVSVLTTMSNRVCSQYAAYGKEKSRRSHLIKLA
Chinese tree shrew	SYLSMVGSCCTSASPTVCFLKERLQSKQLSLLTTLSNRVCSQYAAYGKEKSRLSHLIKLA
Squirrel	SYLSMVGTCCTSASPTVCFLKERLHMRQFSLLTTMSNRVCSQYAAYGKEKSRLSHLIKLA
Ground squirrel	SYLSMVGTCCTSASPTVCFLKERLHMRQFSLLTTMSNRVCSQYAAYGKEKSRLSHLIKLA
Golden hamster	SYLSMVGSCCTSASPTVCFLKERLQIKHLSLLTTMSNRVCSQYAAYGKEKSRLSYLIKLA
Kangaroo rat	SYLSMVGSCCTSANPTVCFLRERLQIKQLSLLTSMSNRVCSQYAVYGKEKLRLSHLIKLA
Mouse	NYLSMVGSCCTSANPTVCFVKERLQMKHLSLLTTMSNRVCSQYAAYGKEKSRLSHLIKLA
Rat	SYLSMVGSCCTSAKPTVCFLKERLQMKQLSLLTTMSNRVCSQYAAYGKEKSRMSHLIKLA
Naked mole rat	IYLSMVGSCCTSPSPNVCFLKERLQIRHLSLLTAMSNQVCSQYAAYGKEKSRLSHLIKLA
Guinea pig	SYLSMVGSCCTSPNPTVCFLKERLQIRHLSLLTTMSNRVCSQYAVNGKEKSQISHLIKLA
Rabbit	SYLSMVGTCCTSASPTVCFLKERLQIKHLSLLTTLSNRVCSQYAAYGKEKSRRSHLIKLA
Pika	SYLSMVGTCCTSPNPTVCFLKERLQIKHLSLLTTLSNRVCSQYAVYGMEKSRRSHLIKLA
Pig	SYLSMVGSCCTSPSPTVCFLKERLQLKHLSLLTIMSNRLCSQYAAYGKDKSRLSHLIKLA
Alpaca	SYLSMVGSCCTSPSPTVCFLRERLQLKHLSLLTTVSNRICSQYAAYGKEKSRLSHLIKFA
Bactrian camel	SYLSMVGSCCTSPSPTVCFLRERLQLKHLSLLTTVSNRICSQYAAYGKEKSRLSHLIKFA
Dolphin	SSLSMVGLCCTSPNPTVCFLKERLQLKHLSLLTTMSNRICSQYSAYGKEKSRLSHLIKFA
Killer whale	SSLSMVGLCCTSPNPTVCFLKERLQLKHLSLLTTMSNRICSQYSAYGKEKSRLSHLIKFA
Cow	SYLSMVGSCCTSPNPTVCFLKERLQLKHFSLLTIMTNRICSQYAAYGKEKSRLSHLIKFA
Sheep	SYLSMVGSCCTSPNPTACFLKERLQLKHLSLLTIMSNRICSQYAAYGKEKSRLSHLIKFA
Goat	SYLSMVGSCCTSPNPTACFLKERLQLKHLSLLTIMSNRICSQYAAYGKEKSRLSHLIKFA
Horse	SYLSMVGSCCTSPSPTVCFLKERLQTKHLSLLTIMSNRLCSQYAAYGKEKSRLSHLIKLA
White rhinoceros	SYLSMVGSCCTSPSPTACFLKERLQIKHLSLLTIMSNRACSQYAAYGKEKSRLSHLIKLA
Cat	SYLSMVGSCCTSPSPTVCFLKERLQMKHLSLLTTMSNRVCSQYAAYGKEKSRLSHLVKLA
Dog	SYLSMVGSCCTSSSPTACFLKERLQMKHLSLLTTMSNRICSQYAAYGKEKSRLSHLIKLA
Ferret	SYLSMVGSCCTSPHPTVCFLKERLQMKHLSLLTTMSNRVCSQYAAYGKEKSRLSHLIKLA
Panda	SYLSMVGSCCTSPSPTVCFLKERLQMKHLSLLTTMSNRICSQYAAYGKEKSRLSHLIKLA
Pacific walrus	SYLSMVGSCCTSPIPTVCFLKERLQMKHLSLLTTMSNRICSQYAAYGKEKSRLSHLIKLA
Weddell seal	SYLSMVGSCCTSPSPTVCFLKERLQMKHLSLLTTMSNRVCSQYAAYGKEKSRLSHLIKLA
Megabat	SYLSMVGSCCTSSSPTVCFLKERLQIKHLSLLTIMSNRICSQYAAYRKEKSRLSHLIKLA
David's myotis	SYLSMVGSCCTSSSPTVCFLKERLQIKHLSLLTIMSNRICSQYAAYEKEKSRLSHLIKLA
Microbat	SYLSMVGSCCTSSSPTVCFLKERLQIKHLSLLTIMSNRICSQYAAYEKEKSRLSHLIKLA
Hedgehog	SYLSMVGSCCTSPNPTICFLKERLQIKQLSLLTTMSNRLCSQHVAYGKEKSRLSHLIKFA
Shrew	TYLSMVGSCCTSSSPTVCFLKERLQMKHLSLLTTMSNRLCSQHVAYGKEKSRLSLFIKLA
Star nosed mole	SYLSMVGSCCTSPNPTVCFLKERLQIKHLSLLTTMSNRLCSQHVAYGKEKSRLSHLIKLA
Elephant	SYLSMVGSCCTSSSPTVCFLKERLQIKHLSLLTVMSNRICSQYVAYGKEKSRLSHLIKLA
Hyrax	SYLSMVGSCCTSSNPTVCFLKERLQIKHLSLLTIMSNRLCSQHVAYGKEKSRLSHLIKLA
Cape golden mole	SYLSMVGSCCTSSRPIECFLKERIQIKHLSLLTTMSNRLCSQHVAYGKDKSRLSHLIKLA
Tenrec	SYLSMVGSCCTSSSTTVCFLKERLQIKHLSLLTMM-NRICSY--AYGMNKSRLSHLIKLA
Armadillo	SYLSMVASCCTSRSSTICFLKERLQMKYLSLLTTISNRVCSQYAAYGKERSRLSHLIKLA
Sloth	SYLSMVASCCTSSSNVV----ERLQIKHLSLLTTMSNRICSQYTVYGKEKSRQSHLIKLA
Tasmanian devil	NYLSMVGTCCTSANPTACFLAERLQNKHLSLLTTMSNRVCSQFAVYGMKKTRFSYIVKYA
Platypus	SYLSMVGTCCFSPKPNTCFLHEKLQSKQISVLTTMSNSMCSRYAAYGK-KFKYSSMLKIA

Human	QKVPTADLEDVLPLAEDITNILSKCCESASEDCMAKELPEHTVKLCDNLSTKNSKFEDCC
Chimpanzee	QKVPTADLEDVLPLAEDITNILSKCCESASEDCMAKELPEHTVKLCDNLSTKNSKFQDCC
Bonobo	QKVPTADLEDVLPLAEDITNILSKCCESASEDCMAKELPEHTVKLCDNLSTKNSKFQDCC
Gorilla	QKVPTADLEDVLPLAEDITNILSKCCESASEDCMAKELPEHTVKLCDNLSTKNSKFEDCC
Orangutan	QKVPTADLEDVLPLAEDITNILSKCCESASEDCMAKELPEHTVKLCDNLSTKNSKFEDCC

Gibbon	QKVPTADLEDVLPLAEDITNILSKCCESTSEDCMAKELPEHTVKLCDNLSTKNSKFEDCC
Rhesus	QKVPTADLEHVLPLAEDVTNILSKCCESASEDCMAKELPEHTVKLCDNLSKKNSKFEECC
Crab-eating macaque	QKVPTADLEHVLPLAEDVTNILSKCCESASEDCMAKELPEHTVKLCDNLSKKNSKFEECC
Olive baboon	QKVPTADLEHVLPLAEDVTNILSKCCESASEDCMAKELPEHTVKLCDNLSKKNSKFEECC
Marmoset	QKVPTADLEDVLPVAEDITNVLSKCCESTSDDCMAKELPLHTVKICEHLSTKNSKFEDCC
Squirrel monkey	QKVPTADLEDVLPVAEDITNVLSKCCESISDDCMAKELPLHTVKICEHLSTKNSKFEDCC
Tarsier	QKVPTADLEDVLPLAEDLTNILSKCCESTSEDCMAKELPEYTVKICDNLSTKNSKFEDCC
Mouse lemur	QKVPAVDLEDILPLAEDVTNILSKCCQSTSEDCMAKELPEYTIKICDNLSTKNSKFGDCC
Bushbaby	QKVPTADLEDVLPLAEDVTNILTKCCPSTSEDCMAKELPEHTVKVCEHLSTKSSKFGDCC
Chinese tree shrew	QKVPTAELEDVLPLAEDINKILSKCCESTAEDCMAKELPEHTVKICDNLSTKNSKFEDCC
Squirrel	QKVPTADLEAVLPLAEDITRILSKCCESTSEDCMAKELPEHTVKICDNLSTKNSKFEDCC
Ground squirrel	QKVPTADLEAVLPLAEDITRILSKCCESTSEDCMAKELPEHTVKICDNLSTKNSKFEDCC
Golden hamster	QKVPTANLEDVVPLAEDLTQILSRCCESTSEDCMAKELPEHTLKICDNLSTKNSKFEECC
Kangaroo rat	QKAPAAELDHVLPLAEDATNILARCCESTSEDCMAKELPEYTVKICDNLSSVSAKIKDCC
Mouse	QKVPTANLENVLPLAEDFTEILSRCCESTSEDCMASELPEHTIKICQNLSKKNSKFEECC
Rat	QKVPTANLEDVLPLAEDLTEILSRCCKSTSEDCMARELPEHTLKICGNLSKKNSKFEECC
Naked mole rat	QKVPTADLKDVLPVAEDMTAILSKCCESTSEDCMAKELPEYTVRVCDNLSKKNSKFENCC
Guinea pig	QKAPTADLKDVLSLAEDITTILSKCCESTSEDCMAKELPEYTVRVCDNLSKKNSKFEECC
Rabbit	QKAPTAALKEVLPLAEDITNILSKCCESTSEDCMAKELPEHTVKICDSLSTKNPKFEECC
Pika	QRVPTADLK---------------------------LPEHTVKICDSLSTKNSKFEECC
Pig	QKVPTANLEDVLPLAEDVATILSKCCDSASEDCMAKELPEYTVKICDNLSSKNSKFTDCC
Alpaca	QKVPTADLKDVLPLAEDVTTILSKCCGSASEDCMAKELPEYTVKICDSLSTKNSKFKDCC
Bactrian camel	QKVPTADLEDVLPLAEDVTTILSKCCGSASEDCMAKELPEYTVKICDSLSTKNSKFKDCC
Dolphin	QKVPTANLEDVLPLAEGVTTILSKCCESASEDCMAKELLEYTVKICDNLSTKNSKFKDCC
Killer whale	QKVPTANLEDVLPLAEGVTTILSKCCESASEDCMAKELPEYTVKICDNLSTKNSKFKDCC
Cow	QKVPTAHLEDVLPLAEDITTILSKCCDSVSEDCI-KELPEYAVKLCDNLSTKNSKFKDCC
Sheep	QKVPTAHLEDVLPLAEDITTILSKCCESASEDCMPKELPEYTVKLCDNLSTKNSKFKDCC
Goat	QKVPTAHLEDVLPLAEDITTILSKCCESASEDCMPKELPEYTVKLCDNLSTKNSKFKDCC
Horse	QKVPTADLEDVLPLAEDITTILSKCCESTSEDCMAKELPEYSLKICDKLSTKNSKFHDCC
White rhinoceros	QKVPTADLEDVLPLAEDITTILSTCCKSTSEDCMAKELPEYSLKICDKLSTKNSKFHDCC
Cat	QKVPTADLEDVLPLAEEITTILSKCCESTSEDCMAKELPEYTVKICDNLATKNSKFKDCC
Dog	QKVPTAELEDVLPLAEEINTVLSKCCESTSEDCMAKELPEYTVKICDNLSTKNSKFKDCC
Ferret	QKVPTAELEDVLPLAEEINTVLSKCCESTSEDCMAKELPEYTVKICDNLSTKDSKFKDCC
Panda	QKVPTAELEDVLPLAEEINTILSKCCESTSEDCMAKELPEYTVKICDNLSTKNSKFKDCC
Pacific walrus	QKVPTANLEDVLPLAEEINTILSKCCESTSEDCMAKELPEYTVKICDNLSTKNSKFKDCC
Weddell seal	QKVPTANLEDVLPLAEEINTILSKCCESTSEDCMAKELPEYTVKICDNLSTKNSKFKDCC
Megabat	QKVPTADLEDVLPLAEDVTTILSKCCESTSEDCMAKELPEHTIKICDNLSTKNSKFNDCC
David's myotis	QKAPTANLEDVLPLAEDVTTILSKCCGSTSEDCMAKELPEHTVKICDNLSTKNDKFRDCC
Microbat	QKAPTANLEDVLPLAEDVTTILSKCCGSTSEDCMAKELPEHTVKICDNLSTKNDKFRDCC
Hedgehog	QKVPTADLKEVLPLAEGITIILSKCCESTSEDCMAKELPEHTVKICNSLSSKNSKFKDCC
Shrew	QKVPTADLKDVLPLAEDITRILSKCCESTSEDCMAKELPEHTVKLCNNLSNKNSKFQDCC
Star nosed mole	QKVPTADLKDVLPLAEDATAILSKCCESTTEGCMAKELPEHTVKICDNLSTKDSKFKDCC
Elephant	QKAPTADMGDVLPLAEDVTNILSKCCESTSEDCMAKELPEHTVKICDNLSTKNSKFEDCC
Hyrax	QKAPSADLGDVLPLAEDITNILSKCCESTSEDCMAKQLPEHTVKICDNLSTKNSKFEDCC
Cape golden mole	QKAPTADLEDVLPLAEDVTNILSKCCESASEDCMAKELPEHTVKICDNLSTKNSKFEDCC
Tenrec	QKAPTAALQDVLPLAEDLTNLLSKCCESPSEDCMAKELPEHTVKICDKLSTKNSKFEDCC
Armadillo	QKAPTADLEDVLLLAEDITEILSKCCDSPSEDCLPKEVGESTIIITHHLLRRHSS-----
Sloth	QKVPTADLEDILPLVEDISEILSKCCGSSSEDCMAKELPEHTVKICDNLATKNSKFEDCC
Tasmanian devil	QKVPTAALEDVVELAEDMTNVLSDCCNSILEDCMANELADHTVKVCEKLSTKDKRFEHCC
Platypus	QKVPSADFKDAEFLSEDSIRMLSKCCDSDAEDCMA-LLPEHVEKVCDRLSTKDSQIQSCC

Human	QEKTAMDVFVCTYFMPAAQLPELPDVELPTNKDVCDPGNTKVMDKYTFELSRRTHLPEVF
Chimpanzee	QEKTAMDVFVCTYFMPAAQLPELPDVELPTNKDVCDPGNTKVMDKYTFELSRRTHLPEVF
Bonobo	QEKTAMDVFVCTYFMPAAQLPELPDVELPTNKDVCDPGNTKVMDKYTFELSRRTHLPEVF
Gorilla	QEKTAMDVFVCTYFMPAAQLPELPDVELPTNKDVCDPGNTKVMDKYTFELSRRTHLPEVF
Orangutan	QEKTAMDVFVCTYFMPAAQLPELPDVELPTNKDVCDPGNTKVMDKYTFELSRRTHLPEVF
Gibbon	QEKTAMDVFVCTYFMPAAQLPELPDVELPTNKDVCDPGNTKVMDKYAFELSRRTHLPEVF
Rhesus	QEKTAMDIFVCTYFMPAAQPPELPEVELPTNKDVCDQGNTKVMDKYTFELSRRTHLPEVF
Crab-eating macaque	QEKTAMDIFVCTYFMPAAQPPELPEVELPTNKDVCDQGNTKVMDKYTFELSRRTHLPEVF
Olive baboon	QEKTAMDIFVCTYFMPAAQPPELPEVELPTNKDVCDQGNTKVMDKYTFELSRRTHLPEVF
Marmoset	QEKTPMDVFVCIYFMPAAQTPELPDVELPTNKDVCDSGNTKALDKYTFELSRRTRLPEVF
Squirrel monkey	QEKTPMDVFVCVYFMPAAQAPELPDVELPANKDVCDSGNTKALDKYTYELSRRTHLPEVF
Tarsier	QEKTPMDIFVCTYFMPAAQPPELPDVELPTNKDVCEQGNTKARDQYAFELSRRTHLPEVF
Mouse lemur	QEKTPMDIFMCTYFMPAAQPPELPDVKLPTSKDVCNPGSTKAMDQYTFELSRRTQVPEVF
Bushbaby	QEKTPMDVFMCTYFMPAAQPPELPDVKLPTGRDVCDHGNTKAVDQYTFELSRRTHVPEVF

Chinese tree shrew	REKTPMDIFVCTYFMPAAQPPELPDIEMPTNKDVCDQGHARVLDQYTFQLSRRTHVPEVF
Squirrel	QEKTPMDIFMCTYFMPAAQPPQLPDVQFPTNKDVCDAKNTKAMDQYAFELSRRTHIPEVF
Ground squirrel	QEKTPMDIFMCTYFMPAAQPPQLPDVQFPTNKDVCDAKNTKAMDQYAFELSRRTHIPEVF
Golden hamster	QEKTPMDIFMCTYFMPAAEPLQLPVIKLPTETDLCGQSTTQAMDRYTFELSRRTRVPEVF
Kangaroo rat	QEKTPMDIFVCTYFMPALQPAQLLPVKLPVTQDLCNQQNTKAVARYTFELNKRTNIPEVF
Mouse	QENTPMNIFMCTYFMPAAEPLQLPAIKLPTGKDLCGQSTTQAMDQYTFELSRRTQVPEVF
Rat	YETTPMGIFMCSYFMPTAEPLQLPAIKLPTSKDLCGQSATQAMDQYTFELSRRTQVPEVF
Naked mole rat	QEEAPLGIFTCIYFMPAALPLELPAIELPTSTEICDQRNTKALDQYTFELTRRTPIPEVF
Guinea pig	REETPLGIFTCVYFMPAAPQVKLQAVELPTNTEVCGQKSTKALDQYTFELSRRTPIPEVF
Rabbit	QEKTPMDIFVCTYFMPAAQPPEPANVELPTSKDVCDSKNINVMDQYTFELSRKTHIPEVF
Pika	NEKTPMGIFVCTYFMPAAQPPELPAVKLPTSKDVCDSTNTNVMDQYTFELGRRTQIPEVF
Pig	QEKTPMDIFICTYFMPAAPRPELPDVKLPTNKDVCDKGNPKVLDQYIFELSRKTHIPEVF
Alpaca	QEKTPMDIFVCTYFMPAAPTPELPDVKLPTNKDVCDKENTEVLDQYAFELSRKTHIPEVF
Bactrian camel	QEKTPMDIFVCTYFMPAAPTPELRDVKLPTNKDVCDKENTKVLDQYAFELSRKTHIPEVF
Dolphin	QERTPMDIFVCTYFMPAAPNPDLPDVKLPTNKDVCDKGNTKVLDQYAFDLSRKTHIPEVF
Killer whale	QERTPMDIFVCTYFMPAAQNPDLPDVKLPTNKDVCDKGNTKVLDQYTFDLSRKTHIPEVF
Cow	QEKTPMEIFVCAYFMPASPNPELPDVKLPMNKDVCDEGNTKVLDQYIFELSRKTRIPEVF
Sheep	QEKTPMDIFVCAYFMPASPNPKLPDVQLPTNKDVCDKGNTNVLDQYIFELSRKTQIPEVF
Goat	QEKTPMDIFVCAYFMPASPNPKLPDVQLPTNKDVCDKGNTNVLDQYIFELSRKTQIPEVF
Horse	QEKTPMDVFVCTYFMPAAQSPELPDVELPTNKDVCDKGNTKPIDQYIFELSARTHIPEVF
White rhinoceros	QEKTPMGIFVCTYFMPAAQPLELPDVELPTNKEVCSKGNTKALDQYTFELSGRTHIPEVF
Cat	QEKTPMDVFMCAYFMPAAPTPELPAVELPTGQDLCDKGNTKAIDQYIFELSRRTPLPEVF
Dog	QEKTPMDIFVCAYFMPAAPTPELPAIELPANADVCDKGNTKAIDQYTFELSRRTHLPEVF
Ferret	LEKTPMDVFVCIYFMPAAPTPELPAIELPTNSDVCDKGNTKAIDQYTFELSRRTHLPEVF
Panda	QEKTPMDVFVCTYFMPAAPTPELPAVELPTSTDVCDKGNTKAIDQYTFELSRRTHLPEVF
Pacific walrus	QEKTPMDVFVCAYFLPAAPTPELPAIEWPTNTDVCDKGNAKAIDQYTFELSRRTHLPEVF
Weddell seal	QEKTPMDVFVCAYFLPAAPTPELPAIEWPTNTDVCDKGNAKAIDQYTFELSRRTHLPEVF
Megabat	QEKTPMDIFMCIYFTPAAQPPELPEVEMPTNKDMCGNGNTRAMDKYAFELSRRTHIPEVF
David's myotis	QERTPMDIFMCVYFTPATQPPELPEVALPTSKDVCGKGNTKAMDRYTFELSRRTDVPEVF
Microbat	QERTPMDIFMCVYFTPATQPPELPEVELPTSKDVCGKGNTKAMDRYTFELSRRTDVPEVF
Hedgehog	QEKTAMDTFVCIYFMQNTQSPELPDVELPQNKDVCGKAHARALDQYAYEVSRRTQLPEVF
Shrew	QEKTPMDVFVCAYFTPAAQSPEMPDAQMPTNKIICGKGSTKAMDQYAFELSRKTHIPEVF
Star nosed mole	QEKTPMDVFVCIYFMPEAQPPELPEVELPTNKDVCSKHSTKVMDQYAFELSRRTHVPEVF
Elephant	QEKTPMDIFVCTYFMPAAQPPELPAVELPTNKEVCDKESTKATDQYIFELSRRTHIPEVF
Hyrax	EEKTPMDVFVCAYFMPAAQPLELPAAELPTNKDVCDKENTEATDQYTFELSRRTHIPEVF
Cape golden mole	QEKTPMDIFVCTYFMPAAQPLKLQAVELPTEKDMCNKGNTKAKDQYIFELSRRTHIPEVF
Tenrec	QEKTPMDIFVCAYFMPAAQPPELPAVDLPTNKAVCSRENTKAKEQYAFELSRRTQIPEVF
Armadillo	-----QLLFTCIVF--SINPHKYPNEILFIYLQLKESHYFFSYGRYAFQLSRSSHIPEVF
Sloth	QEKTPMDVFVCTYFMPAAQPPELPAVELPTNKDICDKGNTKATDQYTFQLSRRTHIPEVF
Tasmanian devil	GESSPMDIFLCIYSMPAAQALKLSEFIKPSHEEICSTENNKALDRYMYEQSRRKNIPEVF
Platypus	QENTPMDIVLCLYSKPPAKSPKPADLPRPTNEDMCSTENPKALDRYIFEIGRRYHVPEVF

Human	LSKVLEPTLKSLGECCDVEDSTTCFNAKGPLLKKELSSFIDKGQELCADYSENTFTEYKK
Chimpanzee	LSKVLEPTLKSLGECCDVEDSTTCFNAKGPLLKKELSSFIDKGQELCADYSENTFTEYKK
Bonobo	LSKVLEPTLKSLGECCDVEDSTTCFNAKGPLLKKELSSFIGKGQELCADYSENTFTEYKK
Gorilla	LSKVLEPTLKSLGECCDVEDSTTCFNAKGPLLKKELSSFIDKGQELCADYSENTFTEYKK
Orangutan	LSKVLEPTLKSLGECCDVEDSTTCFNAKGPLLKKELSSFIDKGQELCADYSENTFTEYKK
Gibbon	LSKVLEPTLKSLGECCDVEDSTTCFNAKGPLLKKELSSFIDKGQVLCADYSENTFTEYKK
Rhesus	LVKVLEPTLKSLGECCDVEDSTTCFNTKGPLLKQELSSFIDKGQKLCAGYSENTFTEYKK
Crab-eating macaque	LVKVLEPTLKSLGECCDVEDSTTCFNTKGPLLKQELSSFIDKGQKLCAGYSENTFTEYKK
Olive baboon	LVKVLEPTLKSLGECCDVEDSTTCFNTKGPLLKQELSSFIDKGQKLCAGYSENTFTEYKK
Marmoset	LSKVLDPTLKRLGECCDVEDSTACFNVKGPLLKKELASFIDKGQELCADYSENTFTEYKK
Squirrel monkey	LSKVLDPTLKRLGECCDAKDSTACFNVKGPLLKKELASFIDKGQELCADYSENTFTEYKK
Tarsier	LSKVLEPTLKSLGECCTVEDSATCFNAKGPLLKKELSSFINKGQELCEDYSENTFTEYKK
Mouse lemur	LSKILEPTLKSLGECCDLKDPTACLKAKSPLLTKELSSFISKGQELCADYSENTFTEYKK
Bushbaby	LSKVLEPTLKSLGECCDLEDPATCLTAKGPQLEKELSSFIAKGQELCADYSENTFTEYKK
Chinese tree shrew	LSKILEPTLKSLGECCVSEDSTACFNAKGPLLKKELSSFVHKGQELCADYSENTFTEYKK
Squirrel	LSKILTLTLKTLSECCDSQNPASCFVTKGSLMKRKQSLFLDKGQELCADYSENTFTEYKK
Ground squirrel	LSKILTLTLKTLSECCDSQNPASCFVTKGSLMKRKQSLFLDKGQELCADYSENTFTEYKK
Golden hamster	LSKVLETTLKSLSECCDAQDSMACFSTQSPILKKQLTSFIERGQEMCADYSENTFTEYKK
Kangaroo rat	LSKILEPTLKSLSECCDLQDPSTCFNDKNPQLTVVLNSFLGKVHELCADYSENTFTEYKK
Mouse	LSKVLEPTLKTLRECCDTQDSVACFSTQSPLLKRQLTSFIEKGQEMCADYSENTFTEYKK
Rat	LSKVLDTTLKTLRECCDTQDSVSCFSTQSPLMKRQLTSFIEKGQEMCADYSENTFTEYKK
Naked mole rat	LSKIVETTLRSLNECCDWEDSAACFTDKGSQLKKKLSSFIEKGQEVCADYSENTFTDYKK
Guinea pig	LSKILEPTLKSLNECCDSEDSASCFTDKGSQLKKELSSFIEKGQELCADYSENTFTEYKK

```
Rabbit              LSKVLEPTLKSLSECCHSADSTACLNAKGPVLKKEVSSFIDKGQELCAGYSENTFTEYKK
Pika                LSKVLEPTLKSLSECCASEDSTACLNAKGPKLKKELSSFIDKGQELCKDYSENTFTEYKK
Pig                 LSKILEPTLKSLDECCHSEDSTACFKAKGPQFKKELSSFIEKGQELCADYSENTFTEYKK
Alpaca              LSKILEPTLRGLAECCNSGESTACLNEKGPQLKKELSSFIEKGQELCADYSENTFTEYKK
Bactrian camel      LSKILEPTLRGLAECCNSGESTACLHEKGPQLKKELSSFIAKGQELCADYSENTFTEYKK
Dolphin             LSKILEPTLKSLDECCHSEDSAACFNAKGPQLTRELSSFIEKGQELCADYSENTFTEYKK
Killer whale        LSKILEPTLKSLDECCHSEDSTACFNAKGPQLTTELSSFIEKGQELCADYSENTFTEYKK
Cow                 LTKILESTLKSLDECCHSESSTACLNAKGPQLTRELSSFIQKGQELCADYSENTFTEYKK
Sheep               LSKILEPTLKSLDECCHSESSAACLNEKGPQLTRELSSFIQKGQELCADYSENTFTEYKK
Goat                LSKILEPTLKSLDECCHSESSAACLNEKGPQLTRELSSFIQKGQELCADYSENTFTEYKK
Horse               LSKILDSTLKNLDECCHSEDSTACLNAKGPQLKKELSSFIGKGQELCADYSENTFTEYKK
White rhinoceros    LSKILEPTLKSLDECCHSEDSAACFKAKGPQLKKELSSFIDKGQELCGDYSENTFTEYKK
Cat                 LSKILEPTLKSLAECCESEDSAACINTQGPQLKKELSSFIDKGQKLCADYSENTFTEFKK
Dog                 LSKILEPTLKSLAECCDSEDSTGCMNAQGPQLKKELSSFIDKGQKLCADYSENTFTEFKK
Ferret              LSKILEPTLKSLDECCDSGDATGCMNAQGPQLKKELSSFIEKGQKLCADYSENTFTEYKK
Panda               LSKILEPTLKSLAECCDSEDSTGCMNAQGPQLKKELSSFIDKGQKLCADYSENTFTEYKK
Pacific walrus      LSKILEPTFKSLAECCDSEDATGCMNAQGPQLKKELSSFIDKGQKLCADYSENTFTEYKK
Weddell seal        LSKILEPTFKSLAECCDSEDATGCMNAQGPQLKKELSSFIDKGQKLCADYSENTFTEYKK
Megabat             LSKILAPTLKSLADCCDSEDSAACFNAKVPQLKKELSSFIDKGQELCADYSENTFTEYKK
David's myotis      LSKILEPTFKTLGECCDSKDPAVCFDAKVPQLKEELSSFIDKGHELCADYSENTFTEYKK
Microbat            LSKILEPTFKSLGECCDSKDPAVCFDAKVPQLKAELSSFIDKGHELCADYSENTFTEYKK
Hedgehog            LSKILEPTLKSLDDCCHSEDSTTCFNMEAPLLKEKLSTFIENGQELCADYSEHTFTEYKK
Shrew               LSKILEPTLNSLGECCSSGDATACFNSKGPQLKEELSSFIEKGQELCADYSENTFTEYKK
Star nosed mole     LSKILVPTLKSLSECCQSEDTTACFNAKGPELKTELSSFIEKGQELCADYSENTFTEYKK
Elephant            LTTVLEPTLISLGECCDSEDSTTCFNTKGPLLKKELSSFIEKGQDMCADYSENTFTEYKK
Hyrax               LSKALQPTLKALGECCDSEDSTACFNTKGPQLKKELSSFIEKGQEICADYSENTFTEYKK
Cape golden mole    LSKALEPTLKSLEECCDSGDYTACFLAKGPLLKKELSSFIEKGQELCADYSENTFTEYKK
Tenrec              LSKALDATLTSLDECCGSGDAAACLQAKGPLLQKELSSFIEKGQEICADYSENTFTEYKK
Armadillo           LSKILDPTLKSLDECCDAKDSTACFKAKAPLLQKELSSFIDKGQELCADYSENTFTEYKK
Sloth               LSKILEPTLKSLGECCDSKDSASCFKTKNPVLQKKLSSFIEKGQELCADYSENTFTEYKK
Tasmanian devil     VTKLHGLMFKVMSDCCKSVEPEACLRSQRPLARNEMALFFTKAEELCGDFSQHTFTEYKK
Platypus            LSKILDGITRAVSGCCSGEDPHTCLGVVRSQMKREMVVYLAKAKELCGDYSELTFTEYKK
```

```
Human               KLAERLKAKLPDATPTELAKLVNKHSDFASNCCSINSPPLYCDSEIDAELKNIL
Chimpanzee          KLAERLKAKLPDATPTELAKLVNKRSDFASNCCSINSPPLYCDSEIDAELKNIL
Bonobo              KLAERLKAKLPDATPTELAKLVNKRSDFASNCCSINSPPLYCDSEIDAELKNIL
Gorilla             KLAERLKAKLPDATPTELAKLVNKRSDFASNCCSINSPPLYCDSEIDAELKNIL
Orangutan           KLAERLRAKLPDATPTELAKLVNKRSDFASNCCSINSPPLYCDSEIDAELKNIL
Gibbon              KLAERLRAKLPDATPTELAKLVNKRSDFASNCCSINSPPLYCDSEIDAELKNIL
Rhesus              KLAEQLRAKLPDATPTELAELVNKRAKFASNCCFTNSPPLYCGSEIDAELKNIL
Crab-eating macaque KLAEQLRAKLPDATPTELAELVNKRAKFASNCCFTNSPPLYCGSEIDAELKNIL
Olive baboon        KLAEQLRAKLPDATPTELAELVNKRAKFASNCCFTNSPPLYCDSEIDAELKNIL
Marmoset            KLAERLGAKLPDVTPKKLAELVDRRSDFASHCCSVNSPPLYCDSEIDTELKNIL
Squirrel monkey     KLSERLRAKLPDVTPKELAGLVDKRSDFASHCCSVNSPPLYCDSEIDTELKNIL
Tarsier             KLAERLKAKLPDATTAELTELVNKRSDFASKCCSINSPPLYCDSEIDAEMKNAL
Mouse lemur         ------------------------------------------------------
Bushbaby            KLAERLRAKLPDAEPTELEALLEKRSDFASKCCFVNSPPLYCDSQFGVHLPLNV
Chinese tree shrew  KLAERLAAKQPDATPSQLAKLVEERSDFASKCCSINSPPLYCDSEIDAVMKNI-
Squirrel            KLEERLRAKMPNATPMELTGVVDKLSDFASKCCSINSPPLYCGSQIDAEMKDIL
Ground squirrel     KLEERLRAKMPNATPMELTGVVDKLSDFASKCCSINSPPLYCGSQIDAEMKDIL
Golden hamster      KLAERLRTKTPNASPGELEDMVDKRSDFASKCCSINSPPLYCSSQIDAEMRDIL
Kangaroo rat        KLTERLRAKYPDVMPDYLEALVHRNSDFASKCCSMNSPPLYCDSLIDNEMKDVL
Mouse               KLAERLRTKTPNTSPAELKDMVEKHSDFASKCCSINSPPLYCSSQIDAEMIDTL
Rat                 KLAERLRTKMPNASPEELADMVAKHSDFASKCCSINSPPRYCSSQIDAEMRDIL
Naked mole rat      KLSERLRAKLPDATPTELAGLVDKHSEFASKCCSINSPPRYCDSQIDAEMKDIL
Guinea pig          KLTERLRTKLPGATPTELAGQVERHSDFASKCCSINSPPRYCDAQVRRHNRVLL
Rabbit              KLSQQLRAKLPEATSAELAELVEKHSDFASKCCSINSPPNYCDSEIDAEIKNLP
Pika                KLSQQLRAKLPDATSMELADVVNKHSDFASKCCSINSPPLYCDSE---------
Pig                 KLAERLRGKWPDATETELEELVKKRSDFASKCCSINSPPLYCDSEVNHDIPSIS
Alpaca              KLAERLRGKLPDATATELKELVDKHSDFASKCCSINSPPLYCDSEIDAEMNTL-
Bactrian camel      KLAERLRGKLPDATAMELKELVDKRSDFASKCCSINSPPLYCDSEIDAKMNTL-
Dolphin             KLAERLRGKLPDATETELEELVDKRSDFASKCCSINSPPLYCNSEIDAVMNTLQ
Killer whale        KLAERLRGKLPDATETELEELADKRSDFASKCCSINSPPLYCNSEIDAVMNTLQ
Cow                 KLAERLRGKFPDATETDLQELVAKRSDFASKCCSVNSPPLYCNSEIDAEINTLQ
Sheep               KLAERLRGKFPDATETDLQELVAKLSDFASKCCSINSPPLYCSSEIDAEINTLQ
```

Goat	KLAERLRGKFPDATETDLQELVAKLSDFASKCCSINSPPLYCSSEIDAEINTLQ
Horse	KLAERLRAKLPDAAATELEELVGKRSDFASKCCSINSPPLYCDSEIDAEMK--N
White rhinoceros	KLAERLRAKLPDATATELEELVEKRSDFASKCCTINSPPLYCDSEIDAEMK--N
Cat	KLAERLSAKLPDVSALELQGLIDKRSDFASKCCSINSPPLYCDSEV--------
Dog	KLAEGLKAQFPDASATDLQGLIDKRSDFASKCCSINSPPIYCDSET-LKAKEIR
Ferret	KLAEQLKAQLPDASALELQGLIDKRSDFASKCCSINSPPLYCDSEIDVEMKNIL
Panda	KLAEQLKAQMPDASSMDLQGLIDRRSDFASKCCSINSPPLYCDSEVR-KFNPLL
Pacific walrus	KLAEQLRAQFPDASAVELQGLTDKRSDFASKCCSINSPPLYCDSEIDVEMKNIL
Weddell seal	KLAEQLRAQLPEASAMELQGLIDKRSDFASKCCSINSPPLYCDSEIDVEMKNIL
Megabat	KLAERLKAKLPDATATELEGLVDKRSDFASKCCSLNSPPLYCDSEIDAEMKNTL
David's myotis	KLTERLRAKLPDAPDTELEELVEKRSDFASKCCSINSPPLYCDAKIDAEMKNTL
Microbat	KLTERLRAKLPDAPDTELEELVNKRSDFASKCCSINSPPLYCDAEIDAEMKNTL
Hedgehog	KLAGRLRTKLPDATDAQLEELIEKRSDFASKCCSINSPPLYCNS----------
Shrew	KLAGRLRAKLPDASDSELEELVDKRSDFATKCCSMNSPPLYCDSEIDAEMKTAL
Star nosed mole	KLAERLRAKLPDATDTELEELVDKRSDFATKCCSINSPPLYCDSELGNQLTFLV
Elephant	KLAERLRAKLPDATPTDLADLVDKRSDFASKCCSINSPPLYCNSQVGAFNSLLT
Hyrax	KLAERLKAKLPDVAPADLAELADKRSDFASKCCSVNSPPLYCDAEIDAQMKILP
Cape golden mole	KLAERLRAKLPDTTPTDLAKLVDKHSDFASKCCYINSPPLYCDSEVGANTIQLL
Tenrec	KLTERLRAKLPDTSATDLAQLVDKHSDFASKCCSINSPPLHCDSEIEAEMKNIL
Armadillo	KLAERLRAKLPEVTSTDLGELVNKFSDFASKCCFINSPPIYCSSEIDAQMNS--
Sloth	KLAERLRAKLSDATSTDLLELVDKFSDFASKCCSINSPPLYCDSE---------
Tasmanian devil	RLTEHFKTKFPETNSKIIKEMVENRTDFASKCCLMNSPPLYCNTQIDTEIKNFC
Platypus	GLTEKFSQKQPDASPATIKELVERRATFASSCCISNAPPRYCSTQVPAPRPLSF

ALPHA-FETOPROTEIN

Human	MKWVESIFLIFLLNFTESRTLHRNEYGIASILDSYQCTAEISLADLATIFFAQFVQEATY
Chimpanzee	MKWVESIFLIFLLNFTESRTLHRNEYGIASILDSYQCTAEINLTDLATIFFAQFVQEATY
Bonobo	MKWVESIFLIFLLNFTESRTLHRNEYGIASILDSYQCTAEINLTDLATIFFAQFVQEATY
Gorilla	MKWVESIFLIFLLNFTESRTLHRNEYGIASILDSYQCTAEISLADLATIFFAQFVQEATY
Orangutan	MKWVESIFLIFLLNLTESRTLHRNEYGIASILDSYQCAAEINLTDLATIFFAQFVQEATY
Gibbon	MKWVESIFLIFLLNFTESRTLHRNEYGIASILDSYQCTAEINLADLATIFFAQFVQEATY
Rhesus	MKWVESIFLIFLLNFTESRTLHRNEYGIASILDSYQCTAEINLPDLATIFFAQFVQEATY
Crab-eating macaque	MKWVESIFLIFLLNFTESRTLHRNEYGIASILDSYQCTAEINLPDLATIFFAQFVQEATY
Olive baboon	MKWVESIFLIFLLNFTESRTLHRNEYGIASILDSYQCTAEINLPDLATIFFAQFVQEATY
Marmoset	MKWVGSIFLIFLLNFTESRTPHRNEYGIASILDSYQCTAEINLTDLATIFFAQFIQEATY
Squirrel monkey	MKWVGSIFLIFLLNYTESRTPLRNEYGIASILDSYQCTAEINLTDLATIFFAQFVQEATY
Tarsier	MKWVESIFLIFLLSFAESRTLHRNEYGIASILDSSQCKEEVNLANLATIFYAQFFQVATY
Mouse lemur	MKWVESIFLIFLLSFTESRTLHRNAYGIASILDSSQCTADMNLADLATIFFAQFVQEATY
Bushbaby	MKWVELFVLIFLLSFTESRTLHRNAYGIASILDSSRCSTEVNLADIATIFFAQFIQEAPY
Chinese tree shrew	MRWLVSISLILLLNFTESRTLHKNAYGIASVLDSSQCSAEVNLVNIATIICAQFVKEATY
Squirrel	MKWVVSIFLAFLLTFTESRTLHNNAYGIASTLDSSQCSAEVNLVDLTTIFFAQFVHGATY
Golden hamster	MEWRVAIFFVFLLNVAESRMLHKHEFGIASTLDSSQCSTEKNMLSLATITFSQFVPEATF
Kangaroo rat	MKYAIPILLISLLSLSESKTLHRNAYGKDSTLDSSQCSSKKNLVNLATIFLAQFVEKATY
Mouse	MKWITPASLILLLHFAASKALHENEFGIASTLDSSQCVTEKNVLSIATITFTQFVPEATE
Rat	MKWSASISFLLLLNFAEPRVLHTNEFGIESTLDSSQCPTEKNMFNVATIVVAQFVQDATK
Naked mole rat	MKWLVSLILIFLLRFTDSRTLHKNAYGIASTLDSSQCSSEMNLADIATIFFAQFVQEATY
Guinea pig	MKLVASVILVFLLCFTDSKTLHKNAYGIASMLDSSQCSSEMNLANVATILFSQFVQDATY
Rabbit	MKWVVSIFLL-LLNFTESKTLHSNAYGIDSILDSSKCSAGMNLVDLATIFVTQFAQEATY
Pika	MKSVISMFLLFLLSVAESRTLHTNEYGIASILDSLQCSAEKNLVDLATISLAQFVPEATY
Pig	MKWVVSIFLIVLLNFTESRTMHENAYGIASILDSSQCSAEMNLVDLATIFFAQFVQEATY
Alpaca	MKWIVSIFLIFLLNFTESRTMHKNAYEIASILDSSQCSAEMNLVGLATIFFAQFVQEATY
Bactrian camel	MKWIVSIFFIFLLNFTESRTMHKNAYEIASILDSSQCSAEMNLVGLATIFFAQFVQEATY
Dolphin	MKWEVSIFLIFLLNFTESRTMQKNAYGIASILDSSPCSAEMNVVDLATIFFAQFVQEATY
Cow	MKWVVSFFLLFLLNFSDSRTMHKNAYGIDSILDSSPCSSGTNLVGLATIFFAQSVQGATY
Horse	MKWVVSILLIFLLNSTESRTMHSNAYGIASALDSFQCSPEMNLVDLATIFFAQFVQEATY
Cat	MKWVISIFLTFLLNFSEPRTMHRNAYGIASILDSSQCSAEMNFVDLATIFFAQFVQEATY
Dog	MKWVVSFFSIFLLNFSESRTMHRNAYGIASILDSSQCSAEMNLVDLATIFFAQFVQEATY
Ferret	MKWVVSIPLIFLLNFSESRTVHRNAYGIVSILDSSQCSAEVNLVDLATIFFAQFVQEATY
Panda	MKWVVSISLIYLLNFSESRTMHRNAYGIASILDSSQCSAEMNLADLATIFFAQFVQEATY
Pacific walrus	MKWVVSISLIFLLNCSESRTMHRNAYGIASILDSSQCSAEMNLVDLSTIFFAQFVQEATY
Weddell seal	MKWVVSISLIFLLNFSESRTMHRNAYGIASILDSSQCPAEMNLVDLATIFFAQFVQEATY
Black flying fox	MKWALSIFL-ILLNITESRTMHSNMYGMASILNSSQCSAEVNLVDLATIFFAQFVQEATY
Megabat	MKWALSIFL--------------------SILNSSQCSAEVNLVDLATIFFAQFVQEATY

```
Microbat             MKWVVSILLIFLLNTTESRTTHNNAYGIASILDSSQCSAEVNLADLATIFFAQFVQEATY
Hedgehog             MKWVVSAFFIFLLSFSESRTMHRNAYGIASILDSSQCTMEVNLLDAFTILFAQFVQDASY
Shrew                MKWVVSIFLIFLLSFTESRTMHKNAYGIASILDSSQCSTEANLVDLATIAFAQFIHEAAY
Hyrax                MKLVVAIFLISLLNFTESRALYKTVSGAASMLNSSQCFSETNLVNLATIFFAQFV-EATY
Elephant             MKWVVAIFLIFVPNFTDSRTLHKNAYGVASLLNSSICSSEMNLVDLATTFFAQFVQEATY
Cape golden mole     MKWIVAISLISLLNFAESRTLHKNAYGIASVLNSSQCSYKTNLADIATIFFAQFVQEATY
Tenrec               MKWLV-ILLISLLRFTESRTLHKNVYGIASVLNSPQCSSEMNIDDLATIFFAQFVPKATY
Armadillo            MKWVVSIFVIFLLNFSESRTMHTNAYGIASVLHSFQCSTEMDLVDLATIFFAQFIQEATY
Sloth                MKWVVSIFVIFLLNFSESRTMHKNAYGIASILHSSQCSSEMDLVDLSTIFFAQFVQEATY
Opossum              MKWEASIFLLLFLSFAESKLLHTSALELGSTSNSPHSITEANLRDLATIFFVQFIPEATY
Tasmanian devil      MKWETSILLLILFGFAEPKILHSSSSDIVSTTNPPQCSTEGNLVDIATIFFAQFIPEATY
Wallaby              MKWETSILLLVLLGFAESKILHTGTSGIASAPSSPQCTTEGNLADIATIFFAQFIPEATY


Human                KEVSKMVKDALTAIEKPTGDEQSSGCLENQLPAFLEELCHEKEILEKYGHSDCCSQSEEG
Chimpanzee           KEVSKMVKDALTAIEKPTGDEQSAGCLENQLPAFLEELCREKEILEKYGHSDCCSQSEEG
Bonobo               KEVSKMVKDALTAIEKPTGDEQSAGCLENQLPAFLEELCHEKEILEKYGHSDCCSQSEEG
Gorilla              KEVSKMVKDALTAIEKPTGDEQSAGCLENQLPAFLEELCHEKEILEKYGLSDCCSQSEEG
Orangutan            KEVSKMVKDALTAIEKPTGDEQSAGCLENQLPAFLEELCHEKEILEKYGLSDCCSQSEEG
Gibbon               KEVSKMVKDVLTAI----GDEQSAG-KYFLLPAFLEELCHEKEILEKYGLSDCCSQSEEG
Rhesus               KEVSKMVKDVLTAIEKPTGDEQSAGCLENQLPAFLEELCHEKDILEKYGLSDCCSQSEEG
Crab eating macaque  KEVSKMVKDVLTAIEKPTGDEQSAGCLENQLPAFLEELCHEKDILEKYGLSDCCNQSEEG
Olive baboon         KEVSKMVKDVLTAIEKPTGDEQSAGCLENQLPAFLEELCHEKDILEKYGLSDCCSQSEEG
Marmoset             MEVSKMVKDVLTAIEKPAGEEQSAGCLENQLPAFLEEMCHEKEILEKYGLSDCCSQSEEE
Squirrel monkey      MEVNKMVKDVLTAIEKSTGEEQSAECFENQLPVFLEEMCHEKEILEKYGLSDCCSQSEEG
Tarsier              EEVSKMVKDVLTTIKKST----DNGCFENQLFTFLEEICHDKEISEKYGLSDCCSQSEEE
Mouse lemur          KEVSKMVKDVLAVIEKPT--ERTAGCFENQISVFLEEICHEKEISEKYGLSHCCSHSEEE
Bushbaby             REISKMVKDVVTVIEKPTGSGQASGCSENQISIFLEEICHEKEISEKYGLSDCCSQSDED
Chinese tree shrew   EEVSKMVKDVLTVIEKPTGSEQASGCFENQLPAFLEEICHEKEISEKYGFSDCCSRSEKE
Squirrel             PEIRKMTNHVLAVIKKPTDE-----CLENQMSAFLEEICHEKEIFEKYGLSDCCSRSGEE
Golden hamster       EEINKMTNDVLTAIKKPTSEEQHGGCLENQMSVFLDEICHEKELSDKHGYSHCCSQSGEE
Kangaroo rat         EEVSKMVTDVLTVIKKPTDSEQPEGCLESPLFAFMDEICHEEEISEKSGLSDCCKQSGEE
Mouse                EEVNKMTSDVLAAMKKNSGD----GCLESQLSVFLDEICHETELSNKYGLSGCCSQSGVE
Rat                  AEVNKMSSDALAAMKENTGD----GCLENQLSVFLDEICHETELSNKYGFSGCCNQSGVE
Naked mole rat       EEVSKMVTDLLTVVKKPTGSEQPAECFENRLSAFLEEICHERAIAGKYGLADCCAHSEEE
Guinea pig           EEVRKMVTDVLTVIKKPTGSEQLAECLENQLSTFLEEICHERATHEKYGLADCCSRS-EE
Rabbit               EEISKMVRDVLTIIKAPNKDSSASWLISLKLPAFLEEICHETEISEKCGLAECCRQGGDD
Pika                 EEVSKMTRDVLAVIEKASNSELPAGCLENQLPAFLEEICHETEISEKYGVAECCRQSGQE
Pig                  KEVNQMVKDVLTVIEKSTGSEQPAGCLENQVSVFLEEICHEEEIPEKYGLSHCCSQSGEE
Alpaca               KEVSKMVRDVLTVIEKSTGSEKPTGCLENQVSAFLEEICHEREIPEKYGLSGCCNQSGEE
Bactrian camel       KEVSKMVRDVLTVIEKSTGSEKPTGCLENQVSAFLEEICHEREIPEKYGLSGCCNQSGEE
Dolphin              KEVSQMVKDVLTVIEKSTGSEQPAGCLENQVSAFLEEICHEKEIPGKYGLSDCCSQSGEE
Cow                  EEVSQMVKDVLTIIEKPTGSKQPAGCLENQVSAFLEEICREKEIPEKYGLSDCCSRTGEE
Horse                KEVSKMVKDVLTVTEKSTGSEQPTGCSENRLSAFLEEICHEEEIPEKYGLSGCCSQNEEE
Cat                  KEVSKMVKDILTVIEKSTGSEQPVGCLENQLSAFLDEICHEKEISEKYGLSDCCSQSEEE
Dog                  KEVSKMVKDILTVIEKSTGSEQPGGCLENQLPAFLEEICHEKEISEKYGLADCCSQREEE
Ferret               KDVSKMVKDTLTVIEKSTGSEQPVGCLEGQLSVFLEKICHEKEISEKYGLSDCCSQSDEE
Panda                KEVSKMVKDILTVIEKSTGSEQPVGCLENQLSAFLEEICEEKEILEKYGLSDCCSQSEEE
Pacific walrus       KEVSKMVKDTLTVIEKSTGSVHPVECLENQLSVFLEEICHEKEISEKYGLSDCCSQSEEE
Weddell seal         KEVSKMVKDTLTVIEKSTGSVHPVECLENQLSIFLEEICHEKEISEKYGLSDCCSQSEEE
Black flying fox     KEVNKMVKDVLTVIEKPNGSEQPSGCLENQLSAFLEEICHEKEIPQKYGLLDCCSQREEE
Megabat              KEVNKMVKD-LTKIY----AEEKIGCAEILLSAFLEEICHEKEIPQKYGLLDCCSQREEE
Microbat             QEVNKMVKDVLPVIGKPTGSEQPAGCLENQLSAFLEEICHEKEISEKYGLSDCCSQHEEE
Hedgehog             PEVSQMAKDVLTKIEKPTGSEQPAGCMEDQISTFLEEICHEKEIIEKYRLSNCCSQSGQE
Shrew                PEVRKMVKDVLTVIEKPLGSDQPVGCLDDQLSAFLDEICHEKEIVGKYKLSDCCNQNGKE
Hyrax                QEVNKM------------------------LSSFLDEICHEKEISDKYRFSDCCDKTEEE
Elephant             QEVNKMLIDRIASLKE----------LYNNLSAFLEEICHEKEICEKYGLSDCCSQREEE
Cape golden mole     QEVTKMSKDVLTVLEKPTGSEQHAECAENQLSAFLEEICHEKEISEKYGLSDCCSKNEEE
Tenrec               QEVNKMTNDVVAVIKKPTGSEQHGECSDNQLSAFLEEMCHEKDISEKYGFSECCAKSAEE
Armadillo            KEVSKMVKDVLSVIEKPTGSEQPAGCLEHKLSAFLEEICHEKEISEKYGLSNCCSKSEEE
Sloth                KEVGIMVKDILSVLEKSAGSEQTEGCLENQLSAFLEEICHEREISEKYGLSNCCSKSEEE
Opossum              EEVDKLVKDFLADFEKLGAKEKSKECLEKQLSALLEEICHDKDISDKHGLADCCSVVGSD
Tasmanian devil      QEVSKLVKDFLADFERPVANEDPKDCLERQLSALLEEICHEKDISEKYGLADCCSKAGGN
Wallaby              QEIGKLVKDFLADFQKIVA-EKPKDCLEKQLPAFLEEICHDKDISEKYGLAGCCSKAGSD


Human                RHNCFLAHKKPTPASIPLFQVPEPVTSCEAYEEDRETFMNKFIYEIARRHPFLYAPTILL
```

```
Chimpanzee            RHNCFLAHKKPTPASIPLFQVPEPVTSCEAYEEDRETFMNKFIYEIARRHPFLYAPTILL
Bonobo                RHNCFLAHKKPTPASIPLFQVPEPVTSCEAYEEDRETFMNKFIYEIARRHPFLYAPTILL
Gorilla               RHNCFLAHKKPTPASIPLFQVPEPVTSCEAYEEDRETFMNKFIYEIARRHPFLYAPTILL
Orangutan             RHNCFLAHKKPTPASIPLFQVPEPVTSCEAYEEDRETFMNKFIYEIARRHPFLYAPTILL
Gibbon                RHNCFLAHKKPTPASIPLFQVPEPVTSCEAYEEDRETFMSKFIYEIARRHPFLYAPTILL
Rhesus                RHNCFLAHKKPTPASIPVFQVPEPVTSCEAYEEDRETFMNRFIYEIARRHPFLYAPTILL
Crab eating macaque   RHNCFLAHKKPTPASIPVFQVPEPVTSCEAYEEDRETFMNRFIYEIARRHPFLYAPTILL
Olive baboon          RHNCFLAHKKPTPASIPVFQVPEPVTSCEAYEEDRETFMNRFIYEIARRHPFLYAPTILL
Marmoset              RHSCFLAHKKPTPTSMQLFQVPEPVVSCEAYEEDRETFMNKYIYEIARRHPFLYAPTILL
Squirrel monkey       RHSCFLAHKKPTPTSMQLFQVPEPVMSCEAYEEDRETFMNKYIYEIARRHPFLYAPTILL
Tarsier               RHKCLLAHRKTAPASIPPFQVPEPVTSCQAYKEDIETYMNRYIYEVARRHPFLYAPTLLA
Mouse lemur           RLSCFLAHKKAAPASIPAFQAPEPVASCKAYEEDRETFLN--------------------
Bushbaby              RLNCLLTHKKPTPVSIPPFQLPEPILSCKAYEEDRAAFINRYIYEIARRNPFLYAPVVLS
Chinese tree shrew    RHDCMLSHKKATPASIPPHQVLEPATSCKAYEENRETFINKYIYEISRSHPFLYAPTILS
Squirrel              RHSCLLAHKKAAPA-LPPFQVPEPATSCKAYEEDRETFMNRCIYEISRRHPFLYAPTILS
Golden hamster        RHQCLLARKKTASASVLPFSFPEAAESCKAYKENREMFMNRVIYEVSRRFPFMYAPAILS
Kangaroo rat          RHKCFLAHKRAAPASITPFPGPDPKRICQAYEENKETFINRYIYDVSRSHPFLYSPTVLS
Mouse                 RHQCLLARKKTAPASVPPFQFPEPAESCKAHEENRAVFMNRFIYEVSRRNPFMYAPAILS
Rat                   RHQCLLARKKTAPDSVPPFHFPETAESCPAYEENRAMSINTFIYDVSKRNPFLYAPTILY
Naked mole rat        RHKCFLARKKAATVSIPPLQAPDVVTSCKVYEENREMFINKYIYEVSRRHPFLYTPTALS
Guinea pig            RHKCLLTRKRAAPASIPPLQVPDMVTSCKAYEENRETVLNKYIYEVSRRHPFLYTPTALS
Rabbit                RHRCFLAHKKAAHASIPPFQVPEPVSGCKAYEENREAFMNRYIYEISRRHPFLYAPTILS
Pika                  RHRCFLAHKKATAASIPPFQLPEADTGCKAYEENREASVNRYIYEISRRHPFLYAPTILS
Pig                   RHNCFLARKKAAPASIPPFQVPEPVTSCKAYEENRELFMTRYIYEIARRHPFLYAPTILS
Alpaca                RHNCFLAHKKATPASIPPFQVPEPVTSCKAYEENREWFMNQYIYEIARRHPFLYAPTLLS
Bactrian camel        RHNCFLAHKKATSASIPPFQVPEPVTSCKAYEENREWFMNQYIYEIARRHPFLYAPTLLS
Dolphin               RHDCFLAHKKAVPASIPPFQVPEPVTSCKAYEEDRELFMNRYVYEIARRHPFLYAPTILS
Cow                   RHDCFLAHKKAAPASIPPFPVLEPVTSCKSYKENRELFINRYIYEIARRHPVLYAPTILS
Horse                 RHNCLLARKKDSPASIPPFQVPEPVTSCKAYEENREMFLNRYLYEIARRHPFLYSSTALY
Cat                   RHNCLLAHKKATPSSIPPFQVPEPVTSCKAYEENRDMFLNRYIYEIARRHPFLYAPTILS
Dog                   RHNCFLAHKKAAPPSIPPFQVAEPVTSCKAYEENRDMFMNRYIYEIARRHPFLYAPTILS
Ferret                RQNCLLAHKKAAPASIPPLQVPEPGTSCKAYEGSKDMFMNRYIYEIARRHPFLYAPTILS
Panda                 RHNCLLGHKKAAPATIPPFQVPEPVTSCKAYEENRDMFMNRYIYEIARRHPFLYAPTILS
Pacific walrus        RHNCLLTHKKAAPASIPPFQVPEPVTSCKAYEENRDMFMNRYIYEIARRHPFLYAPTILS
Weddell seal          RHNCLLAHKKAAPASIPPFQVPEPVTSCKAYEENRDMFMNRYIYEIARRHPFLYAPTILS
Black flying fox      RHNCLLTHKNAAPASIPPFQVPEPVTSCKAYEESREIFMNRCIYEIARRHPFLYAPTLLS
Megabat               RHNCLLTHKNAAPASIPPFQVPEPVTSCKAYEESREIFMNRCIYEIARRHPFLYAPTLLS
Microbat              RHNCFLAHKKASPASIPPFQLPEPVTGCKEYKENREAFMNRYIYEIARRHPFLYAPILLS
Hedgehog              RHDCLLAHKNAAPASIPAFQVPEPVSSCKAFEENRGAFMNQYIYEIARRHPYLYAPAILY
Shrew                 RHSCLLARKKAASDFVSPFQLPEPVSSCKAYEENRETFMNRFIYDIARRHPFLYAPTILS
Hyrax                 RHNCLLAHKKAAPASIPPFQFPEPVTGCKEYEENRDVFMNRYIYEVARRHPFMYAPTVLL
Elephant              RHNCLLARKKAAPASIPPFQFPEPVTSCKAYEENREMFMNQYIYEIARRHPFLYAPTVLF
Cape golden mole      RHNCLLAHKKAAPATIPPFELPEPVTACKAYEENPETFMNRYIYEIARRHPFLYAPTILY
Tenrec                RHNCLLAHKRAAPATLPPFQFPEADAGCRAYEENRESFLNRYIYELARRHPFLYAPTILF
Armadillo             RHNCLLARKKAAPASIPPFQVPEPVTSCQAYEENQKMFMNRYIYEIARRHPFLYAPTILS
Sloth                 RPNCFLAHKKAALASIPPLQVPEPVTSCKAYEENRKAFMNRYIYETARRYPFLYAPKILS
Opossum               RLGCLLAHKRGSAASIPEFQVPEAVHSCKAHGENPATFMNRYIYEITRRHPFLYAPTILS
Tasmanian devil       RLECLLAHKRSSAASIPEFQIPDPSQSCKAHQENRVNFMNRCIYEISRRHPFLYAPAILS
Wallaby               RLECLLDHKRGNAASIPEFQVPEPTQSCKAHQENPVTFMN--------------------


Human                 WAARYDKIIPSCCKAENAVECFQTKAATVTKELRESSLLNQHACAVMKNFGTRTFQAITV
Chimpanzee            WAARYDKIIPSCCKAENAVECFQTKAATVTKELRESSLLNQHACAVMKNFGTRTFQAITV
Bonobo                WAARYDKIIPSCCKAENAVECFQTKAATVTKELRESSLLNQHACAVMKNFGTRTFQAITV
Gorilla               WAARYDKIIPSCCKAENAVECFQTKAATVTKELRESSLLNQHACAVMKNFGTRTFQAITV
Orangutan             WAARYDKIIPSCCKAENAVECFQTKEATVTKELRESSLLNQHACAVMKNFGTRTFQAITV
Gibbon                WAARYDKIIPSCCKAENAVECFQTKAATVTKELRESSLLNQHACAVMKNFGTRTFQAITV
Rhesus                LAARYDKIIPSCCKAENAVECFQTKAASITKELRESSLLNQHACAVMKNFGTRTFHAITV
Crab eating macaque   LAARYDKIIPSCCKAENAVECFQTKAASITKELRESSLLNQHACAVMKNFGTRTFHAITV
Olive baboon          LAARYDKIIPSCCKAENAVECFQTKAASITKELRESSLLNQHACAVMKNFGTRTFHAITV
Marmoset              LAARYDKIIPSCCKVENAVECFQTKGASITKELRENSLFNQHACAVMKNFGLQTFQAITI
Squirrel monkey       LAARYDKIIPSCCKVANAVECFQTMGASITKELRENSLLNQHACAVMKNFGLQTFQAITI
Tarsier               LAGRYDKIIPPCCKADNAVECFQTKAASITKELKESSLLNEHVCAVMKDFGPRTSQAITV
Mouse lemur           ----------------------------------------------------------TV
Bushbaby              FAARYDKIIPPCCKAANAIECFQMKAAPITKELRESSLINQHTCAVMKTFGPRTFQAIIV
Chinese tree shrew    LAARYDKIIPSCCKAENAVECFQTKGAPIAKELRESSLLNQHMCAVMRTFEPRTFQAITV
Squirrel              LAAGYDKIIPLCCKAENAAECFQTKTTSITKELKESSLLNQHVCAVKRTFEPLIFQAITV
```

Golden hamster	LAAQYDKTVPVCCKAENMEECFQAKRASIAKELKEGSLLNEHVCAVIRKFGSRNLQATTI
Kangaroo rat	SAARYAKIMPLCCKAENAAECFQTKTASINKELRESSLLNQHICAVMKHDGPQTLQE---
Mouse	LAAQYDKVVLACCKADNKEECFQTKRASIAKELREGSMLNEHVCSVIRKFGSRNLQATTI
Rat	LAAQYDKAVPACCKADNMEECFQTKRASMAKELREGSMLNEHVCAVIRKFGSRNLQAVLI
Naked mole rat	LAARYDKIFPSCCKAENAAECFQTQATSITKDLSESSLLNQHVCAIIRNFGPRNFQAVTI
Guinea pig	LAARYDKIFPSCCKAENAAECFQTEVAPITKDLQESSLLNQHACAVMRNFGPRTFQAITI
Rabbit	LATRYDKIIPPCCKAENAVECFQTKAAPITKELREVSLLNQHVCSVMRNFSPLTFQAITV
Pika	LAARYDKIIPSCCQTENALECFQTKAAPITKELREVSLLNQHVCAVMRNFEPLTFQAVTV
Pig	LAAQYDKIIPPCCKAENAVECFQTKAASITKELRESSLLNQHMCTVMRQFGARTFRAITV
Alpaca	LAAQYDKVIPPCCKAENAVECFQTKAASITKELRESS-LNQHTCAVTRNFGPRTLQAITI
Bactrian camel	LAAQYDKVIPPCCKAENAVECFQTKAASITKELRESSLLNQHTCAVTRNFGPRTLQAITI
Dolphin	LAAQYDKVIPPCCKAENAVECFQTKAASITKELRESSLLNQHICAVMRKFGPRTFRAITV
Cow	VANQYNKIIPHCCKAENATECFETKVTSITKELRESSLLNQHICAVMGKFGPRTFRAITV
Horse	LASHYDKIISACCKSENAVECFQSKAATITKELRETSLLNQHVCAVIRNFGPRTLQAITV
Cat	LATHYGKIIPPCCKTENAVECFQTKTALITKELRESSLLNQHICAVMRNFGPRTFRAITV
Dog	LAAHYGKIIPLCCKAENAVECFQTKTSLITKELRESSLLNQHICAVMRNFGPRTFRAITV
Ferret	LAGHYGKMIPACCKAENAVECFQTKTALITKELRDSSLLNQHICAVMKNFGPRTFRAITV
Panda	LAAHYGKIIPPCCKAENAVECFQTKTALITKELRDSSLVNQHICAVMKNFGPRTFRAITV
Pacific walrus	LAAHYGKIIPHCCKVENAVECFQTKTALIIKELRDSSLLNQHICAVMKNFGPRTFRAITV
Weddell seal	LAAHYSKIIPHCCKAENAVECFQTKTALIIKELRDSSLLNQHICAVMKNFGPRTFRAITV
Black flying fox	LASHYDKIIPPCCKAENAVECFQTKAASITKELKESSLLNQHICSVMRNFGPRTFQAITI
Megabat	LASHYDKIIPPCCKAENAVECFQTKAASITKELKESSLLNQHICSVMRNFGPRTFQAITI
Microbat	LAAHYDKIIPLCCKAENAIECFQTQAASITKELRESSLLNQHVCAVMRNFGPRTFGAITI
Hedgehog	LAGHYAKIISSCC-AENPVECFQTKEAPVTKELRENSLLNQHMCAVRRTFEPKVFHAIII
Shrew	LAARFDKIIPTCCQAENAVECFQTKGAPLTKELRESNLLNQHVCSVLRKFGSPTFPAIIA
Hyrax	FASRYDKMISLCCE-DNATECFQSKAASLTKEIKESSLINQHVCAVIRKFEFRNIQSIIL
Elephant	LASRYDKIIPLCCKAENATECFQTKGASITKQLGESSSLNQHVCALMRSFGLRNVQAIIF
Cape golden mole	AASYYDKIIPLCCKAENAIECFQTKAAPVTKELRENSLLNQHVCAVIRNFEPRTFQAITV
Tenrec	LASQYDKMIPFCCKADDASECFQTKAASITKELRENNLLNQHVCAVMRNFEPRTFQAITV
Armadillo	LAAHYDKIIPPCCKTENAVECFQTKAASITKELRESSLLNQHICAVMRNFGPRIFRAITL
Sloth	LAALYDKIIPSCCKAENAVECFQTKTASITKELRESSLANQHTCAVMRNFGIRTFQAMTL
Opossum	LAARYDKIISTCCQAENAVECFHNQAAPVTKDLREKSLISQHVCDVLRKFGERSFKAILL
Tasmanian devil	LAARYDQIIFNCCQAENAMECFHTKAAPVLKDLRESSSISQHICGILRKFGDRTFKAIML
Wallaby	-------------------------AAPVTKELRENSLISQHLCGVRRKFGERTFKA---

Human	TKLSQKFTKVNFTEIQKLVLDVAHVHEHCCRGDVLDCLQDGEKIMSYICSQQDTLSNKIT
Chimpanzee	TKLSQKFTKVNFTEIQKLVLDVAHVHEHCCRGDVLDCLQDGEKIMSYICSQQDTLSNKIT
Bonobo	TKLSQKFTKVNFTEIQKLVLDVAHVHEHCCRGDVLDCLQDGEKIMSYICSQQDTLSNKIT
Gorilla	TKLSQKFTKVNFTEIQKLVLDVAHIHEHCCRGDVLDCLQDGEKIMSYICSQQDTLSNKIT
Orangutan	TKLSQKFTKVNFTEIQKLVLDVAHVHEHCCRGDVLDCLQDGEKIMSYICSQQDTLSNKIT
Gibbon	TKLSQKFTKVNFTEIQKLVLDVAHVHEHCCRGDVLDCLQDGEKIMSYICSQQDTLSNKIT
Rhesus	TKLSQKFTKVNFTEIQKLVLDVAHVHEHCCRGDVLDCLQDGEKIMSYICSQQDTLSNKIT
Crab eating macaque	TKLSQKFTKVNFTEIQKLVLDVAHVHEHCCRGDVLDCLQDGEKIMSYICSQQDTLSNKIT
Olive baboon	TKLSQKFTKVNFTEIQKLVLDVAHVHEHCCRGDVLDCLQDGEKIMSYICSQQDTLSNKIT
Marmoset	TKLSQKFTKVNFTEIRKLVLDVAHVHEHCCRGDVVDCLQDGEKIISYICSQQDTLSNKIA
Squirrel monkey	TKLSQKFTKVNFTEIHKLVLDVAHVHEHCCRGDVVDCLQDGEKIISYICSQEDTLSNKIT
Tarsier	TKLSQKFTKVNFTEIQKLVLDVTHVHEQCCRGDVLECLQDGEKIISYVCSQQEYLSSKIA
Mouse lemur	TKLSQKFTKVNFTEIQKLVLDVAHVHEQCCRGDVLECLQDGERIMSYICSQQDAMSSKIA
Bushbaby	TKLSQKFTRVNFTEIQKLVLDVAHIHEQCCRGDVLECLQDGEKIMSYICSQKDTLSTKIA
Chinese tree shrew	TKLSQKFTRANFTDIQKLVLDVAHVHEECCRGNMLECLQDGEKIMSYICSQQEILSSKIG
Squirrel	AKLSQKFPRANFSEIQKLVLDVAHIHEECCRGNVLECLQDGEKIMSYICSRQDALSSKIA
Golden hamster	IKMSQKFPKANFTEIEKLVLDVAHIHEECCQGNAMECLQDGEKIMSYICSQQDILSTKIA
Kangaroo rat	--EKIMSYLCSQEDILSNKIA
Mouse	IKLSQKLTEANFTEIQKLALDVAHIHEECCQGNSLECLQDGEKVMTYICSQQNILSSKIA
Rat	IKLSQKFPKANITEIRKLALDVAHIHEQCCHGNAMECLQDGESVMTHICSQQEILSSKTA
Naked mole rat	TKLSQKFPKANFSEIQKLVLDVAHIHEECCHGNVLECLHDGEKIMSYICSKQDTLSSKIA
Guinea pig	TKLSQKFPKANFSEMQKLVLDVAHVHEECCQGNALECLQDGEKIMSYICSKQDVLSSKIA
Rabbit	VKMSQKLPKANFTEIQKLVLDVAHTHKECCQGNVLECLQDGERIMSYICSQQHILPSTIA
Pika	TKLSQS--QANFTEIQKLVMDVAHIHQQCCQGNVVECLEDGKRIMSYICSQQHILSSRVA
Pig	TKLSQKFPKANFTEIQKLVLDVAHIHEECCRGNVLECLQDAERVVSYVCSQQDTLSSKIA
Alpaca	TKLSQKFPKANFTEIQKLALDVVHTHEECCRGNVLECLQDGEKIMSYICSQQDSLSSKIA
Bactrian camel	TKLSQKFPKANFTEIQKLALDVVHTHEECCRGNVLECLQDGEKIMSYICSQQDSLSSKIA
Dolphin	TKLSQKFPKANFTEIQRLVVDVAHVHEECCKGNVLECLQDGEKIMSYICSQQDILSSKIA
Cow	TKVSQKFPKANFTEIQKLVMDVAHIHEECCKGNVLECLQDGERVMSYICSQQDILSRQIA
Horse	TTLSQRYSKANFTEIQKLVLDVAHAHEECCRGNVEECVQDGEKLISYVCSQEDILSSSIV
Cat	TKLSQKFSKANFTEIQKLVLDVAHIHEECCRGNVLECLQDGEKIMSYLCSRQDILSSKIA

Dog	TKLSQKFSKANFTEIQKLVLDVAHIHEECCRGNVLECLQDGEKIMSYICSQQDILSSKIA
Ferret	TKLSQKFSKANFTEIQKLVLDVAHIHEECCGGNVLECLQDGEKIMSYICSQQDILSSKIA
Panda	TKLSQKFSKANFTEIQKLVLDVAHVHEECCRGDVLECLQDGEKIMSHICSQQDILSSKIA
Pacific walrus	TKLSQKFSKANFTEIQKLVLDVAHIHEECCRGNVLECLQDGEKIMSYICSQQDILSSKIA
Weddell seal	TKLSQKFSKANFTEIQKLVVDVAHIHEECCRGNVLECLQDGEKIMSYICSQQDILSSKIA
Black flying fox	TKLSQKFSKANFTEIQKLALDVAHTHEECCRGNVLECLQDGGKIMSYICSQQATLSSKIA
Megabat	TKLSQKFSKANFTEIQKLALDVAHTHEECCRGNVLECLQDGGKIMSYICSQQATLSSKIA
Microbat	TKLSQKFPQTNFTEIHKLVLDVAHTHEECCRGNVLECLQDAEKIMFYICSQQDTLSSKIA
Hedgehog	AKMSQRFPRANFTEIQKLVLDVVHLHEECCRGNVLECLQDGGKVMSYICSQQDALSSKIT
Shrew	AKLSQKFPKANFSEIEKLVLDVAHVHEECCRGNVLECLQDGGKIMSYICSQQDFLSSKMQ
Hyrax	TKISQKFPRANFTEIQKLALDTTHIHEECCRGNVLDCLHHGQEIMSYICSQQDALSREIA
Elephant	TKMSQKFPRANFTEIQKMTLDSAHIHEECCRGNVLECLHDGQEVMSYICSQQDALSSKIA
Cape golden mole	TKLSQKFTKANFTEIQKLVLDVAHTHKECCRGNVLECLHDGQEIMSYICSQQDNLSSKIA
Tenrec	SKLTQKFPKANFTEIQKLVLDGVHIHKECCRGNVLECLQDGQAMMSYICSQQENLSNKIA
Armadillo	TKLSQKFSKANFTEIQKLTLDVTHIHKKCCRGNVLECLHDGQEIMSYICSQQDTLSSKIA
Sloth	TKLSQKFSKANFTEIQKLVLDVAHTHEECCRGNVLECLQHEQEIMSYICSQQDTLSSKIA
Opossum	TKISQKFPKANFTVIRDLVLDGAHAHIECCKGNVLECLQDRNEIMSYICSQQDVLSSKIQ
Tasmanian devil	TKLSQKFPKANFTVIQNLVLDGAHVHMECCNGNVPECLQDKNEIMSYVCSQKDILSNQVQ
Wallaby	---NEIMSYICSQKDSLSSKIQ

Human	ECCKLTTLERGQCIIHAENDEKPEGLSPNLNRFLGDRDFNQFSSGEKNIFLASFVHEYSR
Chimpanzee	ECCKLTTLERGQCIIHAENDEKPEGLSPNLNRFLGDRDFNQFSSGEKNIFLASFVHEYSR
Bonobo	ECCKLTTLERGQCIIHAENDEKPEGLSPNLNRFLGDRDFNQFSSGEKNIFLASFVHEYSR
Gorilla	ECCKLTTLERGQCIIHAENDEKPEGLSPNLNRFLGDRDFNQFSSGEKNIFLASFVHEYSR
Orangutan	ECCKLTTLERGQCIIHAENDEKPEGLSPNLSRFLGDRDFNQFSSGEKNIFLASFVHEYSR
Gibbon	ECCKLTTLERGQCIIHAENDEKPEGLSPNLNRFLGDRDFNQFSSGEKNIFLASFVHEYSR
Rhesus	ECCKLTTLERGQCIIHAENDEKPEGLSPNLNRFLGDRDFNQFSSGEKNIFLASFVHEYSR
Crab eating macaque	ECCKLTTLERGQCIIHAENDEKPEGLSPNLNRFLGDRDFNQFSSGEKNIFLASFVHEYSR
Olive baboon	ECCKLTTLERGQCIIHAENDEKPEGLSPNLNRFLGDRDFNQFSSGEKNIFLASFVHEYSR
Marmoset	ECCKLTTLERGHCVINAENDEKPEGLSPNLNRFLGDKDFNQFSSEEKNIFLASYVHEYSR
Squirrel monkey	ECCKLTPLERGHCVINAENDEKPEGLSPNLNRFLGDKDFNQFSSEEKNIFLASYVHEYSR
Tarsier	ECCKLPILELGQCIIHAENDEKPEGLSPNLNRFLGDRDFNQFTSGEKNIFLASFVHEYSR
Mouse lemur	ECCKLPTLELGQCIIHAENDEKPEGLSPNLNRFLGDRDFNQFSSGEKNIFLASFVHEYSR
Bushbaby	DCCTMPLLEFGQCIIHAENDDRPEGLSLNLNRFLGDRDFSQFSSTEKNIFLASFVHEYSR
Chinese tree shrew	KCCKLPILELGQCVIHTENDDQPEGLSPNLNRFLGDRDFNQFSSREKNIFLASFIHEYSR
Squirrel	ECCKLPTLELGHCIIHAENDDKPEGLSPTLNRFLGDRDFNQFSSAERNIFLASFVHEYSR
Golden hamster	ECCKLPTLELGYCIIHAENDDKPEGLSPSLDGVLGDRNFGQLSSEEKIMSMARFLYEYSR
Kangaroo rat	ECCKLPVLELGHCIIHAENDDKPEDLSSNLNRFLGDRDFSQFSSEEKNMFLASFLHEYSR
Mouse	ECCKLPMIQLGFCIIHAENGVKPEGLSLNPSQFLGDRNFAQFSSEEKIMFMASFLHEYSR
Rat	ECCKLPTIELGYCIIHAENGDKPEGLTLNPSEFLGDRNFAQFSSEEKLLFMASFLHEYSR
Naked mole rat	ECCKLPLLELGHCIIHAENDDKPEGLSSDLSKFLGDRDFGRFSPGEKTMFLASFIHEYSR
Guinea pig	ECCKLPVLELGHCIIHAENGETPAGLSPELEGFLGDRDFSRFTPGEKTMFLASFIHEYSR
Rabbit	ECCKLPILELGHCIIHAENDDKPEGLSPHLHRFLGDRDFNQFSSQQKNIFLASFVYEYSR
Pika	ECCKLPILELGHCVIHAENDDKPEGLSPNLSGLLGDRDFNQFSSQEKNLFLASFVYEYSR
Pig	ECCKLPTLELGQCIIHAENDDKPEGLSPNLNRFLGERDFNQLSSREKDLSMARFTYEYSR
Alpaca	ECCKLPTLELGHCIIHAENDDKPEGLSPNLNRLLGERDFNQLSSREKDFSLARFTYEYSR
Bactrian camel	ECCKLPTLELGHCIIHAENDDKPEGLSPNLNRLLGERDFNQLSSREKDFSLARFTYEYSR
Dolphin	ECCKLPTLELGHCIIHAENDDKPEGLSPTLNRFLGETDFNQLSSREKDLYMARFTYEYSR
Cow	ECCKLPTLELGHCIIHAENDDKPEGLSPNVNRFLGDRDFNQLSSRDKDLSMARFTYEYSR
Horse	ECCKLPTVELAQCIIHAENDDKPEGLSPNLNRLLGERDFNQFSSKEKDLFMARFTYEYSR
Cat	ECCKLPILELGQCIIHAENDDTPEGLSPNLNRFLGERDFNQFSSREKDLFMARFTYEYSR
Dog	DCCKLPILELGQCIIHAENDGKPEGLSPNLNRFLGERDFNQFSSREKDLFMARFTYEYSR
Ferret	ECCKLPMLELGQCIIHAENDDKPEGLSPNLNRFLGERDFNQFSSREKDLFMARFTYEYSR
Panda	ECCKLPILELGQCIIHAENDDRPEGLSPNLNRFLGERDFNQFSSREKDLFMARFTYEYSR
Pacific walrus	ECCKLPILELGQCIIHAENDDKPEGLSPNLNRFLGERDFNQFSSREKDLFMARFTYEYSR
Weddell seal	ECCKLPILELGQCIIHAENDDKPEGLSPNLNRFLGERDFNEFSSREKDLFMARFTYEYSR
Black flying fox	ECCKLPILELGQCIIHAENDDKPEGLSPTINRFLGERDFNQFSSRDKDLFMARFTYEYSR
Megabat	ECCKLPILELGQCIIHAENDDKPEGLSPTINRFLGERDFNQFSSRDKDLFMARFTYEYSR
Microbat	ECCKLPTLELGQCIIHAENDDKPEGLSPTLNRFLGERDFNQFSSREKDLFMARFTYEYSR
Hedgehog	ECCKLPMLELGQCIIRAENDDKPEDLSSNLNRFLVARDFSQLSPKEKDLSMARYTYEYSR
Shrew	ECCKLPILELGQCIIHAENDEKPGDLSPSLGRFVDARDFSKLPSKEKDISMARFIYEYSR
Hyrax	GCCKLPTLELGHCIIQAENDDKPRGLSPNINTFL-DKDFHLLTSLEKDDLLASFIYEYAR
Elephant	ECCKLPTLELAHCIIQAENDDKPEGLSPNINRFLGDRDFNQFSSLEKDIFLASFTYEYAR
Cape golden mole	ECCKLPALELGHCIIRAENDDKPEGLSPNLGRFLGDRDFNQFSPLEKDLFLASFTHEYSR
Tenrec	ECCKLPPLELAYCIIRAENDDQPEGLSPNLSRFLGDRDFKQFALKEKDIFLASFTHEYSR
Armadillo	ECCKLPILELAQCIIRAENDDKPEGLSPNLKRFLGDRDFHQFSSVEKDLFLARYTYEYSR

Sloth	ECCKLPTLELAQCIIHAENDDKPEGLSPNLNRFLGDRDFNQFSSVEKDFFLGRFAYEYSR
Opossum	ECCSLPLRDQGDCIVNAENDDQPEGLSPDLRRFLGERKFNLYSSEEKDLFLARFVYEYSR
Tasmanian devil	ECCSLPLRDRGECIVGAENDGQPEGLSPNLRGFLEETKFHLYSSAEKDLFLARFVYEYSR
Wallaby	ECCTLPLQEQGECIVSAENDNRPEGLSPDLRRFLGERKFHLHSSEEKDLFLA--------

Human	RHPQLAVSVILRVAKGYQELLEKCFQTENPLECQDKGEEELQKYIQESQALAKRSCGLFQ
Chimpanzee	RHPQLAVSVILRVAKGYQELLEKCFQTENPLECQDKGEEELQKYIQESQALAKRSCGLFQ
Bonobo	RHPQLAVSVILRVAKGYQELLEKCFQTENPLECQDKGEEELQKYIQESQALAKRSCGLFQ
Gorilla	RHPQLAVSVILRVAKGYQELLEKCFQTENPLECQDKGEEELQKYIQESQALAKRSCGLFQ
Orangutan	RHPQLAVSVILRVAKGYQELLEKCFQTENPLECQDKGEEELQKYIQESQALAKRSCGLFQ
Gibbon	RHPQLAVSVILRVAKGYQELLEKCFQTENPLECQDKGEEELQKYIQESQALAKRSCGLFQ
Rhesus	RHPQLAVSVILRVAKGYQELLEKCFQTENPLECQEKGEEELQKYIQESQALAKRSCGLFQ
Crab eating macaque	RHPQLAVSVILRVAKGYQELLEKCFQTENPLECQEKGEEELQKYIQESQALAKRSCGLFQ
Olive baboon	RHPQLAVSVILRVAKGYQELLEKCFQTENPLECQDKGEEELQKYIQESQALAKRSCGLFQ
Marmoset	RHPQLAVSIILRVAEGYQELLEKCFQTENPLECQDKREEELQKYIQESQALAKQSCGLFR
Squirrel monkey	RHPQLAVSIILRVAEGYQELLEKCFQTENPLECQDKGEEELQKYIQESQALAKQSCGLFQ
Tarsier	RHPQLAVPVILRVAKGYQELLEKCFQSENPLQCQDKGEKELQKYIQESQALAKRSCGLFQ
Mouse lemur	RHPDLAVSVILRVAKGYQELLEKCFQSENALECQDKGEEELQRYIQESQALAKRSCGLFQ
Bushbaby	RHPDLAVPVILRVAKGYQELLEKCFQSENPLECQDKGEEELQKYIQESQALAKRSCGLFQ
Chinese tree shrew	RHLELAVPVILRVAKGYQEVLEKCFQSENPLECQDKG--VVQKYIQES-AL-KQTCGLFQ
Squirrel	RHPELAVPVILRVVKGYQEVLEKCGQSEKPLECQDKGEEELQKYTQESQALAKRSCGLFQ
Golden hamster	RHTKFAVSVILRVAKTYQEMMEKCSQSENPVVCQDNGEEEVQKHIQESQALAKQSCALYQ
Kangaroo rat	RHTNLPVPVILRVATGYKEVVEKCFQTENPLECQDKGEEELQKYVQETQALAKQSCGLFQ
Mouse	THPNLPVSVILRIAKTYQEILEKCSQSGNLPGCQDNLEEELQKHIEESQALSKQSCALYQ
Rat	NHPNLPVSVILKTAKSYQEILEKCSQSETPSKCQDNMEEELQKHIQESQALAKQSCDLYQ
Naked mole rat	RHTELAVSIILRVANGYQELLEKCFQSENTLECQNKGEQELQKYVQESQTMATRSCSLFQ
Guinea pig	RHTALAVSIILRIANGYQELLEKCFQSKNPLECQNKGEQELQKFIQESQAMATRSCGLYQ
Rabbit	RHPELPVTVILRITKGYQELLEKCFQTGNPLECQDKGEEELQKYIQESQAVVKRSCDLYQ
Pika	RHPELPVTIVLRTAKGYQEQLEKCAQDENPLACQNKGEEELQKYIQESQEVVKRSCDLYQ
Pig	RHPKLAVPVILRVAKGYQELLEKCSQSENPLECQDKGEEELEKYIQESQALAKRSCGLFQ
Alpaca	RHTKLAVPVILRVAKGYQELLEKCSQSEHPLECQDKGEEESEKYIQESQALAKRSCGLFQ
Bactrian camel	RHTKLAVPVILRVVKGYQELLEKCSQSEHPLECQDKGEEESEKYIQESQALAKRSCGLFQ
Dolphin	RHTKLAVPIILRVARGYQELLEKCSQSGNPPECQDKGEEELEKYIQESQALAKRSCGLFQ
Cow	RHTKLAVPIILRVAKGYQELLEKCSQSENPSECQDKGEEELEKYIQESQALAKRSCGLFQ
Horse	RHTKLAVPVILRVAKGYQEFLEKCSQSENPLECQDKGEEELQKYIQEGQALAKRSCGLFQ
Cat	RHTKLAVPVILRVAKGYQELLEKCSQSENPLECQDKGEEELEKYIQESQALAKRSCGLFQ
Dog	RHTKLAVPVVLRVAKGYQELLEKCSQSENPLECQDKGEEELEKYIQESQALAKRSCGLFQ
Ferret	RHTKLAVPVILRVAKGYQELLEKCSQSENPLDCRDKGEEELEKYIQESQALAKRSCGLFQ
Panda	RHTKLAVPVVLRVAKGYQELLEKCSQSENPLECQDKGEEELEQYIQESQALAKRSCGLFQ
Pacific walrus	RHTKLAVPVVLRVAKGYQELLEKCSQSENRLECQDKGEEELEKYIQESQALAKRSCGLFQ
Weddell seal	RHTKLAVPVVLRVAKGYQELLEKCSQSENRLECQDKGEEELEKYIQESQALAKRSCGLFQ
Black flying fox	RHPKLAVPVILRVAKGYQELLEKCSQSENPLECQDKGEEELEKYIQESQALAKRSCGLFQ
Megabat	RHPKLAVPVILRVAKGYQELLEKCSQSENPLECQDKGEEELEKYIQESQALAKRSCGLFQ
Microbat	RHTKFAVPVILRVAKGYQELLEKCSQSQNPLECQDKGEEELEKYIQENQALAKRSCGLFQ
Hedgehog	RHLELAIPVILRVIKTYLELMEKCAQSENPVECQDKGEEELEKHIQESQALSKRSCGLFQ
Shrew	RHSELSTSAILRSAEEFQKLLENCSQSENPLECQDKGEIEMEKHTQESRAFAKRSCGTFR
Hyrax	RHPELAVPIVLRVAKGYEELLEKCSHSENHLECQDKGEEELEIYIQESQALAKRSCGLFQ
Elephant	RHPELAVPVVLRVVKGYQELSEKCSQSENRLECQDKGEEELEKYIQESQALAKRSCGLFQ
Cape golden mole	RHPKLAVPVILRVAKGYQELLEKCSQSENHLECQDKGEEELEKYIQESQTLAKRSCDLFQ
Tenrec	RHPELAVPVILRVAKGYQELLEKCSQAENRLECQDKGEEELEKYIQESRALAKRSCGLFQ
Armadillo	RHPEFAVTVVLRVAKGYEQLLENCSQSENPLECQDKGEEELEKYIQESQTIAKRTCGLFQ
Sloth	RNTDLAIPVILRVTKGYQELLENCSQSENPLECQDKGEEELEKYIQESQALAKRSCGLFQ
Opossum	RHQDLAVPVILRVAKGYQEALEKCSKTENPSECQDKEEEELERHVQDSQALAKRSCGLFQ
Tasmanian devil	RHQELPVPVILRIAKGYQIALEKCPQTENPLECRDKEEEEINKHIQESRALAKRSCGLFQ
Wallaby	------------------------------------EEEINKYVQDSQALAKRSCGLFQ

Human	KLGEYYLQNAFLVAYTKKAPQLTSSELMAITRKMAATAATCCQLSEDKLLACGEGAADII
Chimpanzee	KLGEYYLQNAFLVAYTKKAPQLTSSELMAITRKMAATAATCCQLSEDKLLACGEGAADII
Bonobo	KLGEYYLQNAFLVAYTKKAPQLTSSELMAITRKMAATAATCCQLSEDKLLACGEGAADII
Gorilla	KLGEYYLQNAFLVAYTKKAPQLTSSELMAITRKMAATAATCCQLSEDKLLACGEGAADII
Orangutan	KLGEYYLQNAFLVAYTKKAPQLTSSELMAITRKMAATAATCCQLSEDKLLACGEGAADII
Gibbon	KLGEYYLQNAFLVAYTKKAPQMTSSELMAITRKMAATAATCCQLSEDKLLACGEGAADII
Rhesus	KLGEYYLQNAFLVAYTKKAPQLTSSELMAITRKMAATAATCCQLSEDKLLACGEGAADII
Crab eating macaque	KLGEYYLQNAFLVAYTKKAPQLTSSELMAITRKMAATAATCCQLSEDKLLACGEGAADII
Olive baboon	KLGEYYLQNAFLVAYTKKAPQLTSSELMAITRKMAATAATCCQLSEDKLLACGEGAADII

Marmoset	KLGEYYLQNVFLIAYTKKAPQLTSSELVAITRKMAATAATCCQLSEDELLACGEGAADII
Squirrel monkey	KLGEYYLQNVFLIAYTKKAPQLTSSELVAITRKMAATAATCCQLSEDELLACGEGAADII
Tarsier	KLGEYYLQNAFLVAYTKKAPQLTSSELIAFTRKMAAAGATCCQLSDDKLLACGEGAADLI
Mouse lemur	KLGEYYLQNAFLVAYTKKAPQLTSSELMALTRKMATAGATCCQLSEDRQLACGEGAADLI
Bushbaby	KLGEYYLQNAFLVAYTKKAPQLTSAELMAFTRKMATAGATCCQLSEDKQLACGEGAADLI
Chinese tree shrew	KLGEYHLQTAFLVTYTKKAPQLTTSELLANTRRMVTAAATCCQLSEDKQSACIETATDLI
Squirrel	KLGQYYLQNAFLVSYVKKVPQMTLTELMALTKKMANTAATCCQLSEDKQLACGEGAADLI
Golden hamster	KLGAYYLQKVFLIAYTRKVPQLTSAELIDLTSKMVSIASTCCQLSEENWSACGEGAADLF
Kangaroo rat	KLGDYYFQN-------------------------------CCPL-EDKRLACGEGAADLI
Mouse	TLGDYKLQNLFLIGYTRKAPQLTSAELIDLTGKMVSIASTCCQLSEEKWSGCGEGMADIF
Rat	KLGPYYLQNLFLIGYTRKAPQLTSAELIDLTGKMVSIASMCCQLSEEKRSACGEGLADIY
Naked mole rat	KLGEYYLQNAFLIAYTKKAPQLTSSELMAFTRKMVTTAATCCQLSGDKQLACGEGAADLI
Guinea pig	KLGEYHLQNSFLITYTKKAPQLTTSELMAFTRKMVTTAATCCKLSGDKQLACGEGAADLI
Rabbit	KLGEYNFQNVFLVSYTKKAPQLTSPELIALTRKMAAKAAMCCHLSEDKRLACAETAADLV
Pika	KSGHYYFQNAFLVAYTKKAPQLTSAELMAFTKKVVTKAAMCCHLSEDKRLACSETMADLV
Pig	KLGEYYLQNAFLVAYTKKAPQLTPPELMALTRKMATTGAACCHLSEDRQLACGEGAADLI
Alpaca	KLGEYYLQNAFLVAYTKKAPQLSPPELMALTRKMAATGATCCHLSEDKQLACGEGAADLI
Bactrian camel	KLGEYYLQNAFLVAYTKKAPQLTPPELMALTRKMAATGATCCHLSEDKQLACGEGAADLI
Dolphin	KLGEYYLQNAFLVAYTKKAPQLTSAELMTLTRKMATTGATCCHLSEDKQLACGEGSADLI
Cow	KLGEYYLQNAFLVAYTKKAPQLTSPELMALTRKMANAGAICCHLSEDKQLACGEGVADLI
Horse	KLGDYYLQNAFLVAYTKKAPQLTPPELIALTRKMATAAATCCQLSEDKQLACGEQVAGLI
Cat	KLGEYYLQNAFLVAYTKKAPQLTPPELMAFTRKMATAAATCCQLSEDKQLACGEGAADLI
Dog	KLGEYYLQNAFLVAYTKKAPQLTPPELMAFTRKMATAAATCCQLSEDRQLACGEGAADLI
Ferret	KLGEYYLQNAFLVAYTKKAPQLTPPELMAFTRKMATAAATCCQLSEDKQLACGEGAADLI
Panda	KLGEYYLQNAFLVAYTKKAPQLTPPELMAFTRKMATAAATCCQLSEDKQLACGEGAADLI
Pacific walrus	KLGEYYLQNAFLVAYTKKAPQLTPPELMAFTRKMATAAATCCQLSEDKQLACGEGAADLI
Weddell seal	KLGEYYLQNAFLVAYTKKAPQLTPPELMAFTRKMATAAATCCQLSEDKQLACGEGAADLI
Black flying fox	KLGEYYLQNAFLVAYTKKAPQLTPPELMAATRKMAIAAATCCQLSEDKQLACGEGAADLI
Megabat	KLGEYYLQNAFLVAYTKKAPQLTPPELMAATRKMAIAAATCCQLSEDKQLACGEGAADLI
Microbat	KLGEYYLQNAFLVAYTKKAPQLTPPELMTYTRKMASAAATCCRLSEEKQLACGEGAADVI
Hedgehog	KLGKYYLQNAFLVAYTKKAPQLTPLELMTFTKKMATAAATCCQLSEDKQLACGEAAADL-
Shrew	KLGNYYLQNTFLVAYTKKAPQMPAPELMVFTREMARAAAACCQLSEDKQLACGEGAADLI
Hyrax	KLGEYYLQNAFLVAYTKKAPQLTTRELIAVTRQMAATGATCCHLSEDKQLACGEGAADLI
Elephant	KLGEYYLQNAFLVAYTKKAPQLTTPELIAITRKMAATGATCCHLSEDKQLACGENAADLI
Cape golden mole	NLGEYYLQNEFLVAYTKKAPQLTTPELLALTKKMAATGTACCHLSEEKQLACGEGAADLI
Tenrec	KLGEYYFQNALLVAYTKKAPQLTTPELIAITRTMAATGASCCQLGEDKQLACGENAADLI
Armadillo	KLGEYPLQNVFLVAYTKKAPQLTSSELMAFTRKMAAAAVACCQLREDKQLACGEGEADQI
Sloth	KLGEYQLQN-FLVAYAKKAPQLTFQELIAFTRKMAAAAATCCQLGEDKQLSCGEEAADQI
Opossum	KLGEYHLQNVFLVAYTKKAPQLISQELISFTKKMAATASLCCQLSEDKQLACGEGAADLI
Tasmanian devil	KLGEYHLQNGFLVTYTKKAPQLTAEELIGYTKKMAATASVCCQLSEDRQLACGESAADLI
Wallaby	KLGEYHLQN---

Human	IGHLCIRHEMTPVNPGVGQCCTSSYANRRPCFSSLVVDETYVPPAFSDDKFIFHKDLCQA
Chimpanzee	IGHLCIRHETTPVNPGVGQCCTSSYANRRPCFSSLVVDETYVPPAFSDDKFIFHKDLCQA
Bonobo	IGHLCIRHEMTPVNPGVGQCCTSSYANRRPCFSSLVVDETYVPPAFSDDKFIFHKDLCQA
Gorilla	IGHLCIRHEMTPVNPGVGQCCTSSYANRRPCFSSLVVDETYVPPAFSDDKFIFHKDLCQA
Orangutan	IGHLCIRHETTPVNPGVGQCCTSSYANRRPCFSSLVVDETYVPPAFSDDKFIFHKDLCQA
Gibbon	IGHLCIRHEMTPVNPGVGQCCTSSYANRRPCFSSLVVDETYVPPAFSDDKFIFHKDLCQA
Rhesus	IGHLCIRHETTPVNPGVGQCCTSSYANRRPCFSSLVVDETYVPPAFSDDKFIFHKDLCQA
Crab eating macaque	IGHLCIRHETTPVNPGVGQCCTSSYANRRPCFSSLVVDETYVPPAFSDDKFIFHKDLCQA
Olive baboon	IGHLCIRHETTPVNPGVGQCCTSSYANRRPCFSSLVVDETYVPPAFSDDKFIFHKDLCQA
Marmoset	IGHLCIRHDTTPVNPGVGQCCTSSYANRRPCFGSLVVDETYVPPAFSGDKFIFHKDLCQT
Squirrel monkey	LGHLCIRHETTPVNPGVGQCCTSSYANRRPCFSSLVVDETYVPPAFSGDKFIFHKDLCQT
Tarsier	LGQLCIRHAATPVNPGVGQCCTSSYANRRPCFSSLVVDETYVPPSFSADKFIFHKDLCQA
Mouse lemur	IGQLCIRHEATPVNPGVGHCCTSSYANRRPCFSSLVVDETYVPPSFSDDKFIFHKDLCQA
Bushbaby	LGQLCIRHDTAAVNPGVAQCCAASYANRRPCFSSLVLDDTYSPPAFSGDKFIFHKDLCQA
Chinese tree shrew	IGQLCIRHETTPINPGIGQCCTSSYANRRPCFSSFVLDPTYIPPLFSADKFVFHKDLCQA
Squirrel	IGHLCIRHEVNPVNPSVGHCCNSSYANRRPCFSSLVIDETYVPPPFSADKFIFHKDLCQP
Golden hamster	IGHLCIRHEAKPLNPGIGHCCNSSYSNRRPCITSLVMDESYVPPPFSADKFTLNKDLCQA
Kangaroo rat	IGQLCIRHAATPLNPGVGRCCDSAYANRRPCFSSLTVDDTYVPPPFSDDKFIFHKDLCQA
Mouse	IGHLCIRNEASPVNSGISHCCNSSYSNRRLCITSFLRDETYAPPPFSEDKFIFHKDLCQA
Rat	IGHLCLRHEANPVNSGINHCCSSSYSNRRLCITSFLRDETYVPPPFSEDKFIFHKDLCQA
Naked mole rat	IGQLCIRHDASPVNPGVGICCNSSYANRRPCFSSLVVDETYVSPPFSADKFIFHKDLCQA
Guinea pig	IGQLCIRHEARPVNPGVGRCCSSSYADRRPCFSRLLVDETYVSPPFSADKFIFHKDLCQA
Rabbit	LGQLCIRHEATPVNPGVGQCCTSSYANRALCFNKLVLDETYVPPPFSADKFIFHADLCQA

```
Pika                   LGQLCIRHEATPLNPGIGHCCTSSYANRALCFHKLVLDETYVPPPFSADKFVFHKDLCQA
Pig                    IGQLCIRHEEMPINPGVGQCCTSSYANRRPCFSSLVLDETYVPPPFSDDKFIFHKDLCQA
Alpaca                 IGQLCIRHEETPVNPGVGQCCTSSYANRRPCFSSLVMDETYVPPPFSDDKFIFHKDLCQA
Bactrian camel         IGQLCIRHEETPVNPGVGQCCTSSYANRRPCFSSLVMDETYVPPPFSDDKFIFHKDLCQA
Dolphin                IGLLCIRHEETPVNPGVGQCCTSSYANRRPCFSSLVVDETYVSPPFSDDKFIFHKDLCQA
Cow                    IGHLCIRHEENPINPGVDQCCTSSYSNRRPCFSSLVVDETYVPPPFSDDKFIFHKDLCQV
Horse                  IGQLCIRHEESPINPGVGQCCTSSYANRRPCFSSLVVDETYVPPPFSDDKFIFHKDLCQA
Cat                    IGQLCIRHEETPVNPGVGLCCTSSYANRRPCFSSLVVDETYVPLPFSADRFIFHKDLCRA
Dog                    IGQLCIRHEETPINPGVGQCCSSSYANRRPCFSSLVVDETYIPSPFSADKFIFHKDLCQA
Ferret                 IGQLCIRHEETPVNPGVGQCCTSSYANRRPCFSSLVVDETYTPSPFSADKFIFHKDLCQA
Panda                  IGQLCIRHEETPVNPGVGQCCTSSYANRRPCFSSLVVDETYIPSPFSADKFIFHKDLCQA
Pacific walrus         IGQLCIRHEETPVNPGVGQCCTSSYANRRPCFSSLVVDETYTPSPFSADKFIFHKDLCQA
Weddell seal           IGQLCIRHEETPVNPGVGQCCTSSYANRRPCFSSLVVDETYTPSPFSSDKFIFHKDLCQA
Black flying fox       IGQLCIRNEETPVNPSVGQCCTSSYASRRPCFSSLVVDETYVPPPFSDDKFIFHKDLCQA
Megabat                IGQLCIRNEETPVNPSVGQCCTSSYASRRPCFSSLVVDETYVPPPFSDDKFIFHKDLCQA
Microbat               IGQLCIRHGETPINAAVGQCCTSSYANRRPCFSSLVVDETYVPPPFSDDKFIFHKDLCQA
Hedgehog               -GHLCIRHEETPINSGVGQCCTSSYANRRPCFSSLVVDETYVPPPFSADKFIFHKDLCQP
Shrew                  IGHLCIRHEEAPINARVGHCCSSSYANRRPCFSNLVMDEAYVPPPFSADKFIFHTDLCQA
Hyrax                  IGHLCIRHEETP-DAGVTRCCNYTYAIRRPCFSNLLVDETYVPPA-SADKFLFHKDLCQA
Elephant               IGQLCIRHEETPVNPGVGHCCNSSYANRRPCFSNLLVDETYVPPAFSAAKFLFHKDLCQA
Cape golden mole       IGQLCIRHEATPINPGVGQCCSSSYANRRPCFSNLLIDETYVPPEFSAAKFHFQKDLCQA
Tenrec                 IGQLCIRHETSPVNPGVGQCCASSYANRRPCFSNLLVDETYVPPEFSAAKFHFQKDLCQA
Armadillo              IGQLCIRHEATPVNPGVGRCCSSSYANRRPCFSNLVVDETYVPPAFSAAKFLFHKDLCQA
Sloth                  IGELCIRHEATPVNPGFGHCCTSSYANRRPCFSNLVVDETYVPPSFSAAKFLFHKDLCQA
Opossum                IGQLCARHEAQPINDGVGHCCDSSYANRRPCFSNFVRDEKYVPPPFSHDQFNFRQDLCHA
Tasmanian devil        IGQLCARHEAQPINDGVAHCCDFSYAHRRPCFSNFVKDEKYVPPPFSYEQFKFQQDLCQA
Wallaby                ------------------------------------------------------------


Human                  QGVALQTMKQEFLINLVKQKPQITEEQLEAVIADFSGLLEKCCQGQEQEVCFAEEGKRLF
Chimpanzee             QGVALQTMKQEFLINLVKQKPQITEEQLEAVIADFSGLLEKCCQGQEQEVCFAEEGQKLI
Bonobo                 QGVALQTMKQEFLINLVKQKPQITEEQLEAVIADFSGLLEKCCQGQEQEVCFAEEGQKLI
Gorilla                QGVALQTMKQEFLINLVKQKPQITEEQLETVIADFSGLLEKCCQGQEQEVCFAEEGQKLI
Orangutan              QGVALQTMKQEFLINLVKQKPQITEEQLEAVIADFSGLLEKCCQGQEQEVCFAEEGQKLI
Gibbon                 QGVALQTMKQEFLINLVKQKPQITEEQLEAVIADFSSLLEKCCQSQEQEVCFAEEGQKLI
Rhesus                 QGVALQTMKQEFLINLVKQKPQITEEQLEAVIADFSGLLEKCCQGQEQEVCFAEEGRRLF
Crab eating macaque    QGVALQTMKQEFLINLVKQKPQITEEQLEAVIADFSGLLEKCCQGQEQEVCFAEEGQKLI
Olive baboon           QGVALQTMKQEFLINLVKQKPQITEEQLEAVIADFSGLLEKCCQGQEQEVCFAEEGQKLI
Marmoset               EGLALHTMIQEFLINLVKQKPQITEEQLGAVTADFAGMLEKCCQGQEQEVCLAEEGQQLI
Squirrel monkey        EGLALHTMIQEFLINLVKQKPQITEEQLGTITADFAGMLEKCCRGQEREVCLAEEGQQLV
Tarsier                QGVALQTMKQEFLINLVKQKPQITEEQLEAVITDFSSLLEKCCQGQEQEVCFAE-GPKLI
Mouse lemur            QGVALQTMKQEFLINLVKQKPQITEEQLEAVIADFSGLLERCCQGQEQDVCFAEEGSKLI
Bushbaby               QGIALQTTKQEFLINLVKQKPQITEEQLEAVIADFSGLLDRCCQGREQEECFAHEGEELI
Chinese tree shrew     QGVALQTMKHEFLINLVKQKPQITDEQLGAVTADFSSLLEKCCQSQEQDACFSEEGLKLI
Squirrel               QGVALQTMKQEFLINLVKQKPQITEEQLEAVIADFSGLLETCCQGQEQEACFAQESPKLI
Golden hamster         QGRALQTMKQELLVNLVKQKPDMTEEQHEAVTADFSSFLEKCCQDQEQEDCFAEEGPKLI
Kangaroo rat           QGRALQTMKQELLISLVRQKPQMTEEQHEAVTADFSGLLEKCCRGQEQEVCFAEEGPKLI
Mouse                  QGKALQTMKQELLINLVKQKPELTEEQLAAVTADFSGLLEKCCKAQDQEVCFTEEGPKLI
Rat                    QGRALQTMKQELLINLVKQKPEMTEEQHAAVTADFSGLLEKCCKDQDQEACFAKEGPKLI
Naked mole rat         HGVALQEMKQELLINLVKQKVQITEGQLETIIQDFSGFLEKCCHHQEQEACFAEEGPKLI
Guinea pig             QGAALQEMKQELLINLVKQKVQITEEQLEAIIPDFSGFVEKCCQHQEQEACFAEEGPKLI
Rabbit                 QGIALQTMKQEFLINLVKQKPQITQEQLEAVIADFSGMLEKCCHGQEREACFAQEGPKLI
Pika                   QGTALQTMKQEFLVNLVKQKLQITQQQLDGVTEDFSELLKKCCQGQEQEACLAEEGPKLI
Pig                    QGVALQTMKQQFLINLVKQKPQITEEQLEAVIADFSGLLEKCCQGQEQEVCFAEEGPALI
Alpaca                 QGVALQTMKQQFLINLVKQKPQITEEQLEAVIADFSGLLEKCCQGQEQETCFAEEGPALI
Bactrian camel         QGVALQTMKQQFLINLVKQKPQITEEQLEAVIADFSGLLEKCCQGQEQETCFAEEGPALI
Dolphin                QGVALQTMKQQFLINLVKQKPQITEEQLEAVIADFSGLLERCCQGQGQEVCFEEEGPALI
Cow                    QGVPLQTMKQQFLINLVKQKPQITEEQLETVVADFSGLLEKCCQSQEQEVCFTEEGPALI
Horse                  QGVALQTMKQQFLINLVKQKPQITEEQLEAVIADFSGLLEKCCQGQGQEVCFSEEGPQLI
Cat                    QGVALQTMKQQFLINLVKQKPQITEEQLEAVIADFSGLLEKCCQGQEQEACFAEEGPKLI
Dog                    QGVALQTMKQQFLINLVKQKPQITEEQLEAVIADFSGLLEKCCQGQEQEACFEEEGPKLI
Ferret                 QGVALQTMKQQFLINLVKQKPQITEEQLEAVIADFSALLEKCCQGQEQDACFEDEGPKLI
Panda                  QGVALQTMKQQFLINLVKQKPQITEEQLEAVIADFSGLLEKCCQGQEQAACLEEEGVKLI
Pacific walrus         QGVALQRMKQQFLINLVKQKPQITEEQLEAVIADFSGLLEKCCQGQEQEACFEEEGPKLI
Weddell seal           QGVALQRMKQQFLINLVKQKPQITEEQLEAVIADFSGLLEKCCQSQEQEACFEEEGPKLI
Black flying fox       QGVALQTMKQRFLINLVKQKPQITEEQLEAVISDFSALLEKCCQGHEQEVCFAEEGPKLI
Megabat                QGVALQTMKQRFLINLVKQKPQITEEQLEAVISDFSALLEKCCQGHEQEVCFAEE-----
```

```
Microbat            QGVALQTMKQQFLINLVKQKPQISEEQLEAVIADFSGLLEKCCQGQEQEVCFAEEGPKLI
Hedgehog            QGVALQTMKQQFLINLVEQKPQITEEQLEAVTADFSALMDKCCKGEVHEACVTEEGPKLI
Shrew               QGVALQTKKQEFLINLVKQKPQITEEQLEAVTADFSALLEKCCRSEKQDVCFAEEGPKLI
Hyrax               QGVALQWMKQEFLINLV-------------------------------------------
Elephant            QGVALQSMKQEFLINLVKQKLQITEEQLEAVIADFSGLLETCCQGQEQEVCLAEEGPKLI
Cape golden mole    PGVALQTMKQEFLINLVKQKPQIREEQLEIVITDFCGLLEKCCQAQEQEDCFAKEGPKLI
Tenrec              QGVALQMRKQEFLINFVKQMPQIREEQLEAVIADFCGLLETCCQAQDQDVCFAEEGPKLI
Armadillo           QGVELQTRKQEFLINLVKQKLQLTEEQLEVVTADFSGLLEKCCQDQGQEDCFAEEGPKLI
Sloth               QGVALQMMKQEFLINLVKHKLQITEEQLEAAIADFSHLLEKCCQSQEQEVCFAEEGHKLI
Opossum             QGEELQKKKQEFLINLVKQKPHITEEQLKAVTTDFTGLLENCCKGKQQEACFAEEGPKLI
Tasmanian devil     QGEELQKKKQEFLINLVKQKPNITEEQLKAVIADFTGLLEKCCKGK-EEACFEEEGPKLI
Wallaby             -----------------------------------------------------------I


Human               SKTRAALGV
Chimpanzee          SKTRAALGV
Bonobo              SKTRAALGV
Gorilla             SKTRTALGV
Orangutan           SKTRAALGV
Gibbon              SKTRAALGV
Rhesus              SKTRAALGV
Crab eating macaque SKTRAALGV
Olive baboon        SKTRAALGV
Marmoset            SKSRAALGV
Squirrel monkey     SKTRAALEV
Tarsier             SKTRAALRV
Mouse lemur         SKTRAALGV
Bushbaby            --TDLILSV
Chinese tree shrew  SKTRAALGV
Squirrel            SRTRAALGV
Golden hamster      SRTRAALGV
Kangaroo rat        SKTRAALGV
Mouse               SKTRDALGV
Rat                 SKTREALGV
Naked mole rat      SKTRTSLGV
Guinea pig          SKTRTSLGV
Rabbit              SKTRAALEV
Pika                SKTRAALGV
Pig                 SKTRASLGV
Alpaca              SKTRTALGV
Bactrian camel      SKTRTALGV
Dolphin             SKTRAALGV
Cow                 SKTRAALGV
Horse               SKTRAALGV
Cat                 SKTRAALGV
Dog                 SKTRAALGV
Ferret              SKTRAALGV
Panda               NSAEWIERI
Pacific walrus      AKTRAALGV
Weddell seal        AKTRAALGV
Black flying fox    SKTRAALEV
Megabat             ---------
Microbat            SKTRAALGV
Hedgehog            SKTRAALGV
Shrew               SKTRAALGA
Hyrax               ---------
Elephant            SKTRAALGV
Cape golden mole    SKTRAALGA
Tenrec              AKTRAALGA
Armadillo           SRTRAALGV
Sloth               SKTRAALGV
Opossum             AKTQEALGV
Tasmanian           SKTQEAF--
Wallaby             AKTQGALGA
```

AFAMIN

Human	MKLLKLTGFIFFLFFLTESLTLPTQPRDIENFNSTQKFIEDNIEYITIIAFAQYVQEATF
Chimpanzee	MKLLKLTGFIFFLFFLTESLTLPTQPRDVENFNSTQKFIEDNIEYITIIAFAQYVQEATF
Bonobo	MKLLKLTGFIFFLFFLTESLTLPTQPRDVENFNSTQKFIEDNIEYITIIAFAQYVQEATF
Gorilla	MKLLKLTGFIFFLFFLPESLTLPTQPRDVENFNSTQKFIEENIEYITIIAFAQYVQEATF
Orangutan	MKQLKLTGFIFFLFFLTESLTLPTQPQDVENFNSTQKFIEENIEYITIIAFAQYVQEATF
Gibbon	MKQLKLTGFIFFLFFLTESLTLPIQPQDVDNFNSTQKFIEENVEYITIIAFAQYVQEATF
Rhesus	------TGFIFFLCFLTESLTLPTQPQDVDNFSSTQKFIEENLEYITIIAFAQYVQEATF
Crab-eating macaque	MKQLKFTGFIFFLCFLTESLTLPTQPQDVDNFSSTQKFIEENLEYITIIAFAQYIQEATF
Olive baboon	MKQLKLTGFIFFLCFLTESLTLTTQPQDVDNFSSTQKFIEENLEYITIIAFAQYVQEATF
Marmoset	MKQLKLTGFIYFLFFLTESLMLPTELQDLDNFNITQKFIEENVEYITIITFAQYVQEATF
Squirrel monkey	MKQLKLTGFIFFLFFFTESLMLPTELQDLDNFNITQKFIEENVEYITIITFAQYVQEATF
Tarsier	MKQLKLTGFVFFLFFVTESLTLPAKPQDVDYFTSTQKYIEENVEYITIIAFAQYVQEATF
Mouse lemur	----------------------------------TQKFIEENIEYITIIAFAQYVQEATF
Bushbaby	MKHFKLSGFIFFLFFFTESLTLPTKPEDVDDFEITQKFVEDNI-----------------
Chinese tree shrew	MKQLELTSFVLFLFFLTESLPLPTKPQDADDFSVIQKFIEENVEYITTIMFSQYIQEVAF
Squirrel	MKQLKFSGFIFFLLFLTESLSLLTETQDVDSFGITQKFIEENTGSITIIAFAQYVQEATF
Golden hamster	MKHIKFIGFIFFLFSLTESLALPTKPQDIDPFLDTHKFIEENTAYLTIIAVAQYVQEVSF
Kangaroo rat	MKQFKLTAFIFSLFFWTESLSLPINPWDIDDFSTTQKFIQENIGSLVIITFAQYVQEATF
Mouse	MRHLKLTGFIFFLLPLTESLALPTKPQDVDHFNATQKFIDENTTYLAIIAFSQYVQEASF
Rat	MRHLKLTGFIFFLLSLTESLALPTKPQDVDHFNATQKFINENVAYLTIIASAQYVQEASF
Naked mole rat	MQQLKLTGFFFLVFSLIDSLTLPTQPQDIGNFNVNQKFIEENITPFTIIAFSRYVQEATF
Guinea pig	MQQLKFTGFIFFLFILIESLTLPTQLQEVDNFNVTQKFINENTASMTIIAFAQYVQEATF
Rabbit	MKQLNLAGIVFFSLFLIASLILPTKPQDVDDFSVTQKFIEENAGYLTIIAFAQYVQEATF
Pika	MRQLNLTDFVFFFLFLTESLTLTTKPQDVDDSSVTQNFIDENTGYLTIIEFAQYVQEATF
Pig	MKQLKLTGFVIFFFFLTESLTLPTKPQDIDDVRITKKFIEDNVVYITIIAFAQYIQEASF
Alpaca	MKQLKLTGFVIFFFFLTESLTLPTKPQDVENSNSTQNFLEYYVLF-TIIAFAQYIQEASF
Bactrian camel	MKQLKLTGFVIFFFFLTESLTLPTKPQDVDDVSVTKKFIEENVEYITIIAFAQYIQEASF
Dolphin	MKQLKLTGF-LFFFFLTESLTLPTKPQDVDDVSITQKFIEDNIGYITIIAFAQYIQEASF
Killer whale	MKQLKLTGF-LFFFFLTESLTLPTKPQDVDDVSITQKFIEDNIGYITIIAFAQYIQEASF
Cow	MKQLKLTGFVIFFFFLTESLTLPTQPQDVDDVRITQKFIDDNIGYITIIAFAQYIQEASF
Sheep	MKQLKLTGFVIFFFFLTESLTLPTQPQDVDDVRITQKFIEDNVGYITIIAFAQYIQEASF
Goat	MKQLKLTGFVIFFFFLTESLTLPTQPQDVDDVRITQKFIEDNVGYITIIAFAQYIQEASF
Horse	MKQLTLTGFVIFLFFLTESLTLPTKLQDVDDASITQKFIEENIGYITVIAFSQYVQEASF
White rhinoceros	MKQLKLIGFVIFLFFLTESLTLPTKLQDVDDASITQKFIEKNIGYITIIAFSQYVQEASF
Cat	MKQLKLTGFVIFLFFLTESLTLSTKPQDVDDVSITQKFIEENVGYITIIIFAQYIQEASF
Dog	MKQLKLTGFVIFLFFVPESLTLPTQPQDLDDVISTQKFIEENVGYITIIVFAQYVQEASF
Ferret	MKQLKLTGFVIFLFFVPESLTLPTQPQDVDDVSITQKFIEENVGYITIIVFAQHIQEASF
Panda	MKQLKLTGFVIFLFFVPESLTLPTQPQDVDDVNITQKFIEENVGYITIIVFAQYIQEASF
Pacific walrus	MKQLKLTGFVIFLFCVPESLTLPTQPQDVDDVSITQKFIEENVGYITIIAFAQYIQEVSF
Weddell seal	MKQLKLTGFVIFLFFVPESLTLPTQPQDVDDVSITQKFIEENVGYITIIAFAQYIQEVSF
Megabat	------------------------------DFRITQKFIEENVGYITIIAFAQYIQEASF
Microbat	MKQLKLTGFVIFLFFLTECLSLPTKPQDVDDFNIIQKFIEDNVGYITIITFAQYVQEASF
Elephant	--
Hyrax	MK-LKLAGFV-FLFFWTETLALLIQPPDV-------------------------------
Tenrec	MEWFKLAGFVICLLFLSESLTLPTNPQDADHLKTTQTFIQDNTEYITIVAFAQYVQEATF
Opossum	MKSIRTFGPVFLFFYLAESLTLPRAPQTKDYLNATQKFLENNIRDVTTVAFAQFVQEATY
Tasmanian devil	MKSIKSIGAVILFFSLAESVTLPEVPQQED-RNATQKFLENNTRDVTTVAFAQFVQEATY
Wallaby	MSQ---IGPVILFFFLVESLTLPKAPDQEDYLNVTQKFLENNIRDV--------------
Platypus	---QEASY

Human	EEMEKLVKDMVEYKDRCMADKTLPECSKLPNNVLQEKICAMEGLPQKHNFSHCCSKVDAQ
Chimpanzee	EEMEKLVKDMVEYKDRCMADKTLPECSKLPNNVLQEKICAMEGLPQKHNFSHCCSKVDAQ
Bonobo	EEMEKLVKDMVEYKDRCMADKTLPECSKLPNNVLQEKICAMEGLPQKHNFSHCCSKVDAQ
Gorilla	EEMEKLVKDMVEYKDRCMADKTLPECSKLPNNVLQEKICAMEGLPQKYNFSHCCSKVDAQ
Orangutan	EEMEKLVKDMVEYKDRCMADKTLPECSKLPNNVLQDKICAMEGLSQKHNFSHCCSKVDSQ
Gibbon	EEMEDLVKDMVEYKDRCMADKTLPECSKLPNNVLQEKICAMEGLPQKYNFSHCCSKVDDE
Rhesus	EEMEKLVKDMVEYKDRCMADKTLPECSKLPNNFLQEKMCAMEGLPQKHNFSHCCSKVGAE
Crab-eating macaque	EEMEKLVKDMVEYKDRCMADKTLPECSKLPNNFLQEKMCAMEGLPQKHNFSHCCSKVGAE
Olive baboon	EEMEKLVKDMVDYKDGCMADKTLPECSKLP------------------------------
Marmoset	EEMEKLVKDMVEYKDSCMANSMLPKCSKLPNDVLQEAICAMEGLPQKHNFSHCCSKADAE
Squirrel monkey	EEMEKLVKDMVEYKDSCMANSMLPKCTKLPNDVLQEAICAMEGLPQKHNFSHCCSKADTE
Tarsier	EEMEMLTKDMKEYRDRCLADRTLPECAKLVNNVLQERFCAMEGLPQKYNFSHCCSKADFE
Mouse lemur	EDVEILVKDMVEYKDRCVANKTLPECFKLP------------------------------

Bushbaby	------------------------------NDVLQEKICAMKGLPQKYNFSHCCGN-DFE
Chinese tree shrew	EELQVMINNMVEYRDKCVADRTLPECSKSANEIFQEKTCSMEGLPQKYNFSHCCSKVDFE
Squirrel	DEMEILVKDIIEYRDKCLAHKTLPECAEIAINVLQGKICSIEGLPQKYNFSHCCSKMGLE
Golden hamster	DEVEMLVKVMMDYRDRCLANRTLPECSKIANKVIQDMICATEGLPQKHNVSHCCSKTDFA
Kangaroo rat	DELEMLVKNVI--KERCLTNLRLPECSEIAKTAL-EITCAGEGSPQKYNFSQCCSKVDVE
Mouse	DEVETLVKVMLDYRDRCWADNTLPECSKTANDAIQDMLCDMEGLPQKHNFSHCCGKAGFP
Rat	EEVEMLVKVMLDYKDRCLADSTLPECSKIANDAIQDMLCDMKGLPQKHNFSHCCRQAGFQ
Naked mole rat	DDMEILIKDVMKYKDKCLANKTLLECSKPANNVLQDMICSVEGLPQKHNFSHCCSKVDFE
Guinea pig	DDMEMLIKDMMKYKDKCLGDKTLPECSKLANNVLQEMMCSMEGLPQKYNFSHCCRKVDFK
Rabbit	EEVQMLMKDMLEYKDKCLADKTLPECAETANNILQEKICAMEGLSQRHNFSHCCSKVDFE
Pika	EEVERLMNDMLEHRRKCMADKTLSECSETTNNILQEKMCAMEGLPQRYNFSHCCSKVDFE
Pig	EEVEMLVKAMKEYRDRCLADMTLPECSKLANDVLLENICAMEGLPQKYNFSHCCHKVDFE
Alpaca	EEVEMLAKAMTEYRDKCLADRTLPECSKLANDVLLENICAMEGLPQKHNFLHCCRKIDVE
Bactrian camel	EEVEMLAKAMTEYRDKCLADRTLPECSKLANDVLLENICAMEGLPQKHNFLHCCRKIDVE
Dolphin	EEVEMLVKAMTEYRDKCLADMTLPECSKLANDVLLENICTMEGLPQKHNFSHCCHKADFE
Killer whale	EEVEMLVKAMTEYRDKCLADMTLPECSKLANDVLLENICTMEGLPQKHNFSHCCHKADFE
Cow	EEVEMLVKAMTEYRDKCLADRTLPECSKLANEVLLENICAMEGLPQKYNFSHCCHKVDFE
Sheep	EEVEMLVKTMAEYRDKCLADRTRPECSKLTNEVLLENICAMEGLPQKYNFSHCCRKVDFE
Goat	EEVEMLVKTMAEYRDKCLADRTRPECSKLTNEVLLENICAMEGLPQKYNFSHCCRKVDFE
Horse	EEVDRLVKIMTKYKGQCSGEVTLPDCPRLTNDILLEKTCAVKGLPQKYNFSHCCSMVDLE
White rhinoceros	EEVEILVKIMTKYGEQCLDEVTIPDCPRLTNDILLERTCAVKGLPQKYNFSHCCSMVDFE
Cat	EEVEMLVKTMTEYRDKCLADMTLPECSKLANDVLLENICALEGLPQKHNFSHCCSKVDLE
Dog	EEVEMLVKAMTEYRDKCLVDMTLPVCSKLANDVLLENICALEGLPQKHNFSHCCSKVDLE
Ferret	EEVEVLVNAMKEYRDKCLGDRTLLMCSKSANDVLLENICALEGLPQKHNFSHCCSKVDLE
Panda	EEVEVLVKAMTEYRDKCLGDVTLPVCSKLATDVLLENICALEGLPQKHNFSHCCSKVDLE
Pacific walrus	EEVEVLVKAMTEYRDKCLGDVTLPMCYKLANDVLLENICALEGLPQKHNFSHCCSKVDLE
Weddell seal	EEVEVLVKAMTEYRDKCLGGMTLPMCSKLANDVLLENICALEGLPQKHNFLHCCGKVDLE
Megabat	EEAEMLRKAMTEYKDQCLANMTLPMCSKLPNDVLQEMICAIEGLPQKHNFSHCCNKVDFE
Microbat	EEVEMLAKTMTEYRDQCLANSKLPQCSKLPNDALQEAICAMEGVPQKHNFSYCCNKGGFE
Elephant	--
Hyrax	--
Tenrec	EEVEMFAKEMMALKDKCVADMALPECSGLPNDILQKSICAVEGLPQKYNFSHCCAKSDLE
Opossum	EEITGLVKQMILYRDKCVADMQQPECSELAIYVMQDKICRVKELSDKYGLSECCTKNSTN
Tasmanian devil	EEITELVKQMIHYIDKCMANMELPECSRLANDVIQDKICEVKELSEKYGFSECCTEDISK
Wallaby	--
Platypus	EDISKMVENLVALKNRCTAQEKLPECSKTEVGESEDEMCAGKELSEKYGYADCCSQDEKN

Human	RRLCFFYNKKSDVGFLPPFPTLDPEEKCQAYESNRESLLNHFLYEVARRNPFVFAPTLLT
Chimpanzee	RRHCFFYNKKSDVGFLPPFPTLDPEEKCQAYESNRESLLNHFLYEVARRNPFVFAPTLLT
Bonobo	RRHCFFYNKKSDVGFLPPFPTLDPEEKCQAYESNRESLLNHFLYEVARRNPFVFAPTLLT
Gorilla	RRLCFFYNKKSDVGFLPPFPTLDPEEKCQAYESNRESLLNHFLYEVARRNPFVFAPTLLT
Orangutan	RRLCFFYNKKADVGFLPPFPTLDPEEKCQAYESNRESLLNHFLYEVARRNPFVFAPTLLT
Gibbon	RRLCFFYNKKADVRFLPPFPTLDPEEKCQAYESNRESLLNHFLYEVARRNPFVFAPTLLT
Rhesus	RRLCFFYNKKADVGFLPPFPTLDPEEKCQSYESNRESLLNHFLYEVARRNPFVFAPTLLT
Crab-eating macaque	RRLCFFYNKKADVGFLPPFPTLDPEEKCQSYESNRESLLNHFLYEVARRNPFVFAPTLLT
Olive baboon	--
Marmoset	RRICFFYNKKANVGFLPPFPTLDPEEKCQAFKNNKESFLNHFVYEVARRNPFVFAPTLLT
Squirrel monkey	RRLCFLYNKKANVGFLPPFPTLDPEEKCQAFKNNKESFLNHFVYEVARRNPFVFAPTLLT
Tarsier	RRLCFFFNKKADVEFLPPFPTWDPEEKCQAYKNNRESFLNHYMYEVARRYPFVFATTLLT
Mouse lemur	--
Bushbaby	RRSCFFLNKKDGVGFLPPFPTLDPEEKCQAFKKSSESFLNHYVYEVARRNPFVFAPTLLS
Chinese tree shrew	RRLCFFYNKKGDGEFQSPFPTLEPEEKCQAYQNVRKPYL---------------------
Squirrel	RRLCFLYNKKADVGFLPPFPTLDPEEKCQAYKNNRESFLNRYTHEVARRNPFAFGPTLLT
Golden hamster	RRLCFFYNKKAKTGFLPPFPTLDPEEKCQAYKNNNGSFLNLYMYEVARRNPFVFSPVLLT
Kangaroo rat	RR--FLYKKKSTVGFLPPFPTLDPEEKCQAYKNDRESFINCYSHELATRNPFGFASILLM
Mouse	RRLCFFYNKKANVGFLPPFPTLDPEEKCQAYKNNSESFLHLYMYEVARRNPFVFAPVLLA
Rat	RRLCFFYNKKANVGFLPPFPTLDPEEKCQAYKNNSESFLNLYMYEVARRNPFAFAPVLLN
Naked mole rat	RRHCFLYNKKADVGFLPPFPILDP-EKCEAYKNTRESFLNQCMYEVARRNPFVFAPTLLI
Guinea pig	RRLCFFYNKKANVGFLPPFPTLDPEEKCEAYKNTRDSFLNQYIYEVARRNPFVFAPTLLT
Rabbit	RRLCFFYNKKADVGFLPPFLTLDPEEKCQAYKNSRDSFLNHYIYEVARRNPFAFAPTLLT
Pika	RRLCFFYNKKADVGFLPLFLTLDPEEQCQAYKNNRDSFLNHYIYEFARRNPFAFAPTLVT
Pig	RRLCFLHNKKAEVGFLPPLPTLDPEEKCQTYKTNKESFLNNYIYEVSRRNPFVFAPTLLT
Alpaca	RKLCFLHNKKADVGFLPPLPTLDPEEKCQTFKNNRESFLNNYIYEISRRNPFVFAPTLLT
Bactrian camel	RKLCFLHNKKADVGFLPPLPTLDPEEKCQTFKNNRESFLNNYIYEISRRNPFVFAPTLLT
Dolphin	RKHCFLHNKKADVGFLPPLPTLDPEEKCQTYKNNRESFLNNYIYEVSRRNPFVFAPTLLT

Killer whale	RKHCFLHNKKADVGFLPPLPTLDPEEKCQTYKNNRESFLNNYIYEVSRRNPFVFAPTLLT
Cow	RRLCFFHNKKADIGLLPPLPTLDPEEKCQTYKNNRESFLNNYVYEVSRRNPFVFAPTLLT
Sheep	RRLCFFHNKKADIGFLPPLPTLDPEEKCQTYKNNRESFLNNYIYEVSRRNPFVFAPTLLT
Goat	RRLCFFHNKKADIGFLPPLPTLDPEEKCQTYKNNRESFLNNYIYEVSRRNPFVFAPTLLT
Horse	RERCFFHNKKSDVGFLPPLPTLDLEEKCQTYKNNRESFLNNCIYEVSRRNPFVFAPTLLT
White rhinoceros	RERCFFHNKKSDIGFLPPLPTLDLEEKCLTYKNNRESFLNN-------------------
Cat	KKLCFFHHKKADVGFLPPLPTLDPEEKCQTYKNNRESFLNNYIYEVSRRNPFVFAPTLLT
Dog	RKLCFFHNKKADAGFLPPLPTLDPEEKCQTYKSNRESFLNNYIYEVSRRNPFVFAPTLLT
Ferret	RKLCFFHNKKADVGFLPPLPTLDPEEKCQTYKNNRESFLNNYVYEVSRRSPFVFAPTLLT
Panda	RKLCFLHNKKTDVGFLPPLPTLDPEEKCQTYKNNRESFLNNYIYEISRRNPFVFAPTLLT
Pacific walrus	RKLCFFHNKKADVGFLPPLPTLDPEEKCQTYKNNRESFLNNYIYEVSRRNPFVFAPTLLT
Weddell seal	RKLCFFHNKKADVGFLPPLPTLDPEEKCQTYKNNRESSLNNYIYEVSRRNPFVFAPTLLT
Megabat	RRLCFFRNKKAGVEFLPSPLTLDPEEKCQAYKNNSESFLNNYIYEVSRRNPFVFAPTLLM
Microbat	RRLCFFRNKKADVGFLPPLPTLDPEVKCQAYKNNSESFLSNYIYEVSRRNPFVFTPTLLT
Elephant	--
Hyrax	--
Tenrec	RRGCFYLNKKADVGFLPPLPMADAEKKCQEYKQKKDLFLKKYLYEVARRNPFVFVTTLLS
Opossum	RLPCFFQRKSADASFLTPFQGPDPEKACQEYAEDPEAFLNHFIYEIARQNPFGFAPTLLS
Tasmanian devil	SRLCFFERKRTDVSFLTPFQIPDPEKGCQEYEADQETFLNHYIYEIARRNPFIFAPTLLA
Wallaby	---YVYEIARQNPFLFAPTLLS
Platypus	RHRCFFLRKRVDLGFLLHYD-LKPEEECKAYKDDPEAVWNQYIYEIARRQPFLYTPTLLF

Human	VAVHFEEVAKSCCEEQNKVNCLQTRAIPVTQYLKAFSSYQKHVCGALLKFGTKVVHFIYI
Chimpanzee	VAAHFEEVAKSCCEEQNKVDCFQTRAIPVTQYLKAFSSYQKHVCGALLKFGTKVVHFIYI
Bonobo	VAAHFEEVAKSCCEEQNKVDCFQTRAIPVTQYLKAFSSYQKHVCGALLKFGTKVVHFIYI
Gorilla	VAAHFEEVAKSCCEEQNKVNCFQTRAIPVTQYLKAFSSYQKHVCGALLKFGTKVVHFIYI
Orangutan	VAAHFEEVAKSCCEEQNKVNCFQTRAIPVTQYLKAFSSYQKHVCGALLKFGTKVVHFIYI
Gibbon	VVAHFEEVAKTCCEEQNKVNCFQTRAIPVTQYLKAFSSYQKHVCGALLKFGTKVVHFIYI
Rhesus	VAAHFEEVAKSCCEEQNKVNCFQTRAIPVIQYLKAFSSYQKHVCGALLKFGTKVVHFIYI
Crab-eating macaque	VAAHFEEVAKSCCEEQNKVNCFQTRAIPVIQYLKAFSSYQKHVCGALLKFGTKVVHFIYI
Olive baboon	-------------------------AIPVIQYLKAFSSYQKHVCGALLKFGTKVVHFIYI
Marmoset	VAAHFEDVATSCCEEQNKVNCFQTRAVPVMQYLKAFSSYQNNVCGTLLKFGTKIINSINI
Squirrel monkey	VAAHFGDVTKSCCEEQNKVNCFQTRAVPVMQYLKAFSSYQNNVCGVLLKFGTKIINSINI
Tarsier	VATRFEEVAKPCCEEQYRVDCFQTRVAPVTQYLKTWSSYSKNVCGAYFKFGPKVLNSINV
Mouse lemur	-----------------------TRAAPVIQYLKAFSSYQKNVCGAYLKFGTKVLNFINI
Bushbaby	AATRFAEAAGACCEDQREVGCFQKRAAPIMQYLRAFSSYQKNVCGALLKFGPEGLNSINI
Chinese tree shrew	-------------------------AAPITRYLKASSSYQKTLCGAYMKFGPKILKSINV
Squirrel	VAARFEEVAKTCCEEQQKANCFQAKAAPITQYLKAMSSYQKTVCGAFLKFGPKVLNSINI
Golden hamster	VAARFEEAATTCCEQQEKATCFQAKVVPITQYLKASSSYQGTVCGALMKFGPKVLNSINT
Kangaroo rat	AAASFEELAKRCGEQQ-KDNCFQTNAV-------------------------------NI
Mouse	VAAWFEEAATTCCEQQQKATCFQAKAAPITQYLKASSSYQRNVCGALIKFGPKVLNSINV
Rat	VAARFEEAATTCCEQQQKATCFQDKAAPITQYLKALSSYQRNVCGALLKFGPKTLNSINI
Naked mole rat	VAARFEEVVKTCCAEHQKANCFQAMATPVTQYLKAFSSYQKNVCGALMKFGPKVLNSINI
Guinea pig	VAARFEEVAKTCCEEQQKANCFQEKATPITQYLKAFSSYQKNVCGALMKFGPKVLNSINI
Rabbit	VAAQFEELTNRCCEEQHKASCFGVRAAPVTQYLKAMSSYQKNVCGAFLKFGSNVLNSMNI
Pika	VAARFEEVVSTCCEEQHKASCFGVRAAPVTQYLKALSSYQKNVCGAFMKFGPQVLNSINI
Pig	VAARFEEMAETCCEEQEKANCFRSKAEPFIQYLKALSSYQKNVCGALLKFGPQILQSINI
Alpaca	VAARFEEMTKTCCEEQEKAYCFRTKAEHFIQYLKALSSYQKNVCGALMKFGPQILQSINT
Bactrian camel	VAARFEEMTKTCCEEQEKAYCFRTKAEHFIQYLKALSSYQKNVCGALMKFGPQILQSINT
Dolphin	AAARFEEMTKTCCEEQEKANCFRTKAETFIQYLKALSSYQKNVCGALMKFGPQILKSINI
Killer whale	AAARFEEMTKTCCEEQEKANCFRTKAETFIQYLKALSSYQKNVCGALMKFGPQILKSINI
Cow	VAARFEEMTKTCCEEQEKANCFQTKAEPFIYYLKALSSYQKNACRALMKFGRQILQSINI
Sheep	VAARFEEMTKTCCEEQEKANCFRTKAEPFIYYLKALSSYQKNVCGALMKFGPQILQSINI
Goat	VAARFEEMTKTCCEEQEKANCFQTKAEPFIYYLKALSSYQKNVCGALMKFGPQILQSINI
Horse	VATRFEEMAKTCCEEQDKANCFRTKAEPVIQYLKALSSYQKNVCGALVKFGPQVLKSINI
White rhinoceros	-------------------------AESVIQYLKALSSYQKNVCGALMKFGLQVLKSINL
Cat	VAARFEEMTKTCCEEQDKANCFRTKAEPVIQYLKASSSFQKNVCGALIKFGLQVLESINV
Dog	VAARFEEMTKTCCEEQDKANCFRTKAELVIQYLKASSSFQKNVCGALMKFGLQVLESINV
Ferret	VAARFEEMTKTCCEAQDKANCFRTNAEPVIQYLKASSSFQKNVCGAFMKFGPQVLGLINV
Panda	VAARFEEMTKTCCEEQDKANCFRTKAEPVIQYLKASSSFQKNVCGALMKFGLQVLESINV
Pacific walrus	VVARFEEMTKTCCEEQDKANCFRTKAEPVIRYLKASSSFQKNVCGALMKFGLQVLESINV
Weddell seal	VAARFEEMTKTCCEEQDKANCFRTKAEPVIQYLKASSSFQKNVCGALMKFGLQVLESINI
Megabat	LAARFEVITKTCCEEQDKANCFQTKAKSVIQFLKALSSYQKNVCGAFLKFGLQVLNSINI
Microbat	VMGLIEEMAKTCCEEQDKANCFRIKAVPVIQYLKAFSSFQKNVCGAFLRFGPTVLKSIYI
Elephant	--YI

Hyrax	---YI
Tenrec	VATRFEDVAKTCCEEEDKASCFQTKASPVIKYLKENSAFEKTLCGASMRFGPRIITLINA
Opossum	GAAIYKEAITRCCQEENKHECFHLKVTPITNGLKERSASQKNTCGVLMKYGPKVLKHLKV
Tasmanian devil	LVARYKEVSTSCCQEENKHECFRIKATPITNDLKEISAKQQNICGVLRKFGPKGVK-FKI
Wallaby	LAACYKEVSTSCCQEEKKHGCFH-TATTITNNLKELSAKQ-NLCGVLMHFGPKGVKLHKA
Platypus	HAQEYEKVVQACCSEENKLQCFQTKGTLVTNDLRWIHEIHKHLCRIAMGFGKRALRSGKV

Human	AILSQKFPKIEFKELISLVEDVSSNYDGCCEGDVVQCIRDTSKVMNHICSKQDSISSKIK
Chimpanzee	VILSQKFPKIEFKELISLVEDVSSNYDGCCEGDVVQCIRDTSKVMNHICSKQDSISSKIK
Bonobo	AILSQKFPKIEFKELISLVEDVSSNYDGCCEGDVVQCIRDTSKVMNHICSKQDSISSKIK
Gorilla	AILSQKFPKIEFKELISLVEDVSSNYDGCCEGDVVQCIRDTSKVMNHICSKQDSISSKIK
Orangutan	AVLSQKFPKIEFKELISLVEDVSSNYDGCCEGDVVQCIRDTSKVMNHICSKQDSISSKIK
Gibbon	AVLSQKFPKIEFKELISLVEDVSSNYDGCCEGDVVQCIRDTSKVMNHICSKQDSISSKIK
Rhesus	AVLSQKFPKIEFKELISLVEDISSNYEGCCEGDVVQCIRDTSQVA---------------
Crab-eating macaque	AVLSQKFPKIEFKELISLVEDISSNYEGCCEGDVVQCIRDTSKVMNHICSKQDSISSKIK
Olive baboon	AVLSQKFPKIEFKELISLVEDISSNYEGCCEGDVVQCIRDTSKVMNHICSKQDSISSKIK
Marmoset	AILSQKFPKIEFEELISLIEDVSSNYDECCEGDVVHCIRHMSKIMNHICSKQDAFSSKIK
Squirrel monkey	AILSQKFPKIEFKELISLIEDVSSTYDECCEGDVVHCIRHMSKIMNHICSKQDAVSSKIK
Tarsier	ATLSQSLPKMNFKDLTSLVEVVSSIYDGCCEGDVVQCIRDMNKFMSHLCSKPVSNSSKIK
Mouse lemur	AVLSQKFPKIEFKELTSLIEDVSSKYDGCCEGDVVQCVRDTSKVMNHICSKQGSISSKIQ
Bushbaby	AVLSQKFPKVEFQELTSLLEDVSFKYDACCEGDVVGCIRSSSKVASRICSKQESISSKIK
Chinese tree shrew	AVFSQKFPRVEFKELLSLIEEISSMYDGCCEGDAVQCIRDSAKVMNHICSKQDSISTKIK
Squirrel	AVFSKKFPKIEFKELTSLLEDVSSMYDGCCEGDVLQCIRYQSQVMSHICSKQESISSKIK
Golden hamster	AVFSKKFPKIGFQNLTSLLEDVSTMYDVCCEGDVVQCIRSQ-------------------
Kangaroo rat	VVLGRKFPKRKFHELTSLLKDVLSMYDGCCLGDFVNCIHAQNQVYRYICSEQDSISSKIK
Mouse	AVFSKKFPKIGFKDLTTLLEDVSSMYEGCCEGDVVHCIRSQSQVVNHICSKQDSISSKIK
Rat	AVFSKKFPKIGFEDLTSLLEDVSSMYDGCCEGDVVQCIRSQSQVMHHICSKQDSISSKIK
Naked mole rat	AVFSKKFPKIEFRELTSLLQEVSSMYDGCCEGDALQCIHDQSKVMRHIFSKQDSISSKIK
Guinea pig	AVFSKKFPKIEFRELTSLLKDVSSMYYGCCEGDVLQCIHDQSKVMSHICSKQDSISSKIK
Rabbit	AVLSQKFPKIGFKELTSLLEDVSSTYDGCCEGDVVHCTYGMGKIMSHICSKQDSISSKIK
Pika	AILSQKFPKIGFKELTSLLEDVSSKYDGCCKGDVIHCIHGMDKVVSHICSKQDSISSKIK
Pig	AVLSQKFPKIEFKQLTSLLEDISSKYDGCCGGDVVHCIRGRSKVMSHICSKQDSISSKIK
Alpaca	AVLSQKFPKIEFKELTSLLKDVSSKYDGCCEGDVVECIHGRSKIMSHICSKQDSISSKIK
Bactrian camel	AVLSQKFPKIEFKELTSLLKDVSSKYDGCCEGDVVECIHGRSKIMSHICSKQDSISSKIK
Dolphin	AVLSQKFPKIEFKELISLLEDVSSKYDGCCEGDVVQCIHGRSKVMSHICSKQDSISSKIK
Killer whale	AVLSQKFPKIEFKELISLLEDVSSKYDGCCEGDVVQCIHGRSKVMSHICSKQDSISSKIK
Cow	AILSQKFPKIGFKQLTSLLEDVSSKYDGCCEGDVVQCIRGRSKVMSHICSKQDSISSKIK
Sheep	AILSQKFPKIGFKQLTSLLEDVSSKYDGCCEGDVVQCIHGRSKVMSHICSKQDSISSKIK
Goat	AILSQKFPKIGFKQLTSLLEDVSSKYDGCCEGDVVQCIRGRSKVMSHICSKQDSISSKIK
Horse	AILSQKFPKIEFKELTSLLEDVSSKYEGCCEGDAVKCIRRRSKIMSHICSKQDSISSKIK
White rhinoceros	AILSQKFPKIEFKELTSLLEDISSKYEACCEGDGVQCIRGRSKVMSHICSKQDAISSKIK
Cat	AILSQKFPKIEFKELTSLLEDVSAKYDGCCEGDAVQCIRGRSKVMSHICLKQDSISSKIQ
Dog	AILSQKFPKIEFKELTSLLEDVSSKYNGCCEGDAVQCIRDRSKVMNHICSKQDSISSKIR
Ferret	AILSQKFPKIEFKELISLLEDVSSNYHGCCEGDAVQCIRDRSKVLSHICSKQDSISSKIK
Panda	AILSQKFPKIEFKELTSLLKDVSSKYDGCCEGDAVQCIRDRSKVMSHICSKQDSISSKIK
Pacific walrus	AILSQKFPKIEFKELTSLLKDVSSKYDGCCEGDAMQCIHERSKVMSHICSKQDSISSKIK
Weddell seal	AILSQKFPKIEFKELTSLLKDVSSKYDGCCEGDAVQCIHDRSKVMSHICSKQDSISSKIK
Megabat	AILSQKFPKIEFKELTSLLEDVSSKFNGCCEGDAVHCIRGWSKVMDHICSKQDSISSKIK
Microbat	AIFSQKFPKTEFEELTSILEDVSSKYDGCCEGDAVQCIRGRSKLMSHICSKQDSISSKIK
Elephant	AVLSRKFPEIEF-EFISLLEDVASKYDGSCEGDTVECIQAMNNVMSHICTKQDPISSKVK
Hyrax	ARFRQDMP-TEFKK--PLLADISSKFDGCPKGD-VHCV-VTSKIVSHICIKQGSISSKIK
Tenrec	AIIGQKFPKIEFKTLTSTVKHISSTDGGCCEGDAVGCIHTRSMAVNHICTNQDSISTKVK
Opossum	VEFSQKFPKIDFKDLGILLENMDHLFDECCEGDVVQCFRNEAKILNYICSKPDSISSKII
Tasmanian devil	VEISQKFPKIDFSLLNKLLEDIPNMVDGCCEGDVVHCFRLEAKIINHICSRPDSLSSKIR
Wallaby	VEISQKFPKIP-KELDILPKDIPIMIDGCWEGDVVYCLHSKAKITNYLCSKPDSISSKIR
Platypus	LYFSQQFPKMELATLLNVMPDLLGEQDGCCEGDVIECFRSRAELVSSMCSNQKAISSQFS

Human	ECCEKKIPERGQCIINSNKDDRPKDLSLREGKFTDSENVCQERDADPDTFFAKFTFEYSR
Chimpanzee	ECCEKKIPERGQCIINSNKDDRPKDLSLREGKFTDSENVCQERDADPDTFFAKFTFEYSR
Bonobo	ECCEKKILERGQCIINSNKDDRPKDLSLREGKFTDSENVCQERDADPDTFFAKFTFEYSR
Gorilla	ECCEKKILERGQCIINSNKDDRPKDLSLREGKFTDSENVCQERDADPDTFFAKFTFEYSR
Orangutan	ECCEKKIPERGQCIINSNKDDRPKDLSLREAKFTDSENVCQERDADPDTFFAKFTFEYSR
Gibbon	ECCEKKIPERGQCIINSNKDDRPKDLSLREAKFTDSENVCQERDADPDTFFAKFTFEYSR

```
Rhesus                ---------------------------------------CLEEVSFSNPFDCRFTFEYSR
Crab-eating macaque   ECCEKKIPERGQCIINSNKDDRPKDLSLREAKFTDSENVCQERDADPDIFFAKFTFEYSR
Olive baboon          ECCEKKIPERGQCIINSNKDDRPKDLSLREAKFTDSENVCQERDADPDTFFAKFTFEYSR
Marmoset              ECCEKKIPERDQCIINSNKDDRPKDLSLREAKFTDSENVCQERDADPDTFFAEFNFEYSR
Squirrel monkey       ECCEKKIPERDQCIINSSKDDRPKDLSLREGKFTDSENVCQERDADPDTFFAEFNFEYSR
Tarsier               ECCEKKPLERDECIMNTSKEDRTKDLSLREAKFTDSKNVCQERDTDPDNFFTEFTYEYSR
Mouse lemur           ECCEKKIPERGECIINSNKDDRPEDL--REAKFTDSETLCQERDTDPDNFFAEFTYEYSR
Bushbaby              ECCEKEVPERGECIIYSNKDDRPKDLSPGEAKFTDSENVCQGRDADPDNFFAEFTYEYSR
Chinese tree shrew    DCCGKKMLER------------------------------------------FTYEYSR
Squirrel              ECCEEKMPEREACIINSSKDGRPQDLSLREAKFTDNENVCQERDTDPGNFFAEFLYEYSR
Golden hamster        --------------------------------FTDSKNVCQERDSNQDGLLNEFTYEYSR
Kangaroo rat          KCCEKDILECEECIINSNKDDMPE-YSLGKSKYTGSENVCQEKDTYPDSFFAE-IYKNSK
Mouse                 VCCEKKTLEREACIINANKDDRPEGLSLREAKFTESENVCQERDSDPDKFFAEFIYEYSR
Rat                   ACCEKKPPERADCIINANKDDRPEDLSLRTPKFTDSENVCQERDSEQDKFFAEFLYDYSR
Naked mole rat        QCCEKKIPEREDCIINLNKDDRPKNVSLTEAKFIHIEHMCQERETDPEHFFARFLYEYSR
Guinea pig            ECCEKKIPEREDCIINSNKDDRPKNVSLTEAKLTGSEHLCQERDTDPDNFFAKFLYEYSR
Rabbit                ECCEKKIPERGECIINSNKDDRPKDLSLREAKLTDNENLCEERDTDPDNFFAAFLYEYAR
Pika                  ECCEKKIQERGECILNSNKDEKPKYLSSREAKFTDNENLCEERNTDPNTFFAEFLYEYAR
Pig                   ECCEKKIPERGECIIYSNKDDRPNDLSPREAKFTESENVCEERDADPETFLAEFLYEYSR
Alpaca                GCCEKKIPERGECIIYSNKDDRPNDLSLRETKFTESNNVCEERNADRGNFKTEFLYEYAR
Bactrian camel        GCCEKKIPERGECIIYSNKDDRPNDLSLRETKFTESNNVCEERNADRGNFKTEFLYEYSR
Dolphin               ECCEKKIPERGECIIYSNKDDRPNNLPLREAKFTESENVCEERDADQENFMAEFLYQYSR
Killer whale          ECCEKKIPERGECIIYSNKDDRPNNLPLREAKFTERENVCEERDADQENFMAEFLYQYSR
Cow                   DCCEKKIPERGECIIYSNKDDRPNDLSLREAKFIESDNVCEKRDADQANFMAEFLYEYSR
Sheep                 DCCEKNIPERGECIIYSNKDDRPNDLSLREAKFIESDNVCEKRDADQANFMAEFLYEYSR
Goat                  DCCEKNIPVRGECIIYSNKDDRPNDLSLREAKFIESDNVCEKRDADQANFMAEFLYEYSR
Horse                 DCCEEKIPKRGECIIYSNKDDRPKDLSLREAKFTENENVCEQRDANPDIFMAEFLFEYSR
White rhinoceros      ECCEEKIPKRGECIIYSNKDDRPKDLSLREAKFTENENVCEQRDADQDTFMAEFLYEYSR
Cat                   ECCEKKIPERGECIISSNKDDRPKDLSLREAKFTESENVCEERDANQTSFMAEFLYEYSR
Dog                   ECCEKKMPERGECIISSNKDDRPKDLGLREAKFTESENVCEERDANQTIFMAEFLYEHAR
Ferret                ECCEKKIPERGECIIASNKDNRPEDLALREAKFTESENVCEERDANQTIFMAEFLYEYSR
Panda                 ECCEKKMPERGECIVSSTKDDRPKELALREVKFTESENVCEERDANQTIFMAEFLYEHSR
Pacific walrus        ECCEKKMPERGECIISSNKDDRPKDLALREVKFTESENVCEERDANQTIFMAEFLYEHSR
Weddell seal          ECCEKKMPERGECIISSNKDDRPKDLALREVKFTERENVCEERDANQTIFMAEFLYEHSR
Megabat               ECCEKKIPERGECIISSNKDDRPKDLSLKETKFTENKNVCEERNADQDIFMAEFLYEYSR
Microbat              ECCEKKIPERGECIIQS-KDDKPKDLSLKEEKFTENKNVCEERNADQDTFMAKFLYEYSR
Elephant              ECCE-KNPDLGECIIYLGKDQKPKGLSLRETKFADRK-VC--R--HQENFMAEYSYEYSK
Hyrax                 QCSGK-TSEHHEC-----KEEKPKGLSLREVRFT--KDVCQHRDADQENF----------
Tenrec                ECCDKPILERGDCIVNLGRDEKPSDLSPREAKFTDSKDVCQHRDADQENFMIEFLYEYSR
Opossum               DCCQLTIPQGGECIINSGNDDKPEDLSPRAERFTEGDDVCQSLHEDKENFQAKFLYEYSR
Tasmanian devil       DCCQLTIPERGECIIHSENDDKPKDLSPRVERFLEGDDVCQRLHEDKENFLIEFLHEYSR
Wallaby               DCCQLAVP-RGECI------------SLRAEIFTEGDDVCQHLQEDKENFLT--------
Platypus              NCCDKPVPERGECIFRSVREDAPQDLPPTEEKFIRAQDVCQRYADKKDGFLDEFVYEYSR


Human                 RHPDLSIPELLRIVQIYKDLLRNCCNTENPPGCYRYAEDKFNETTEKSLKMVQQECKHFQ
Chimpanzee            RHPDLSIPELLRIVQIYKDLLRNCCNTENPPGCYRYAEDKFNETTEKSLKMVQQECKHFQ
Bonobo                RHPDLSIPELLRIVQIYKDLLRNCCNTENPPGCYRYAEDKFNETTEKSLKMVQQECKHFQ
Gorilla               RHPDLSIPELLRIVQIYKDLLRNCCNTENPPGCYRYAEDKFNETTEKSLKMVQQECKHFQ
Orangutan             RHPDLSIPELLRIVQIYKDLLRNCCNTENPPGCYRYAEDKFNETTEKSLKMVQQECKHFQ
Gibbon                RHPDLSIPELLRIVQIYKDLLRNCCNTENPPGCYRYAEDKFNETTEKSLKMVQQECKHIQ
Rhesus                RHPDLSIPGLLRIVQIYKDLLRNCCNTENPPGCYRYAEDKFNETTEKSLKMVQQECKHFQ
Crab-eating macaque   RHPDLSIPGLLRIVQIYKDLLRNCCNTENPPGCYRYAEDKFNETTEKSLKMVQQECKHFQ
Olive baboon          RHPDLSIPGLLRIVQIYKDLLRNCCNTENPPGCYRYAEDKFNETTEKSLKMVQQECKHFQ
Marmoset              RHPDLSIPQLLRVVQMYKDLLRNCCNTENPPDCYRYAEDKFNEATEKSLKKIQQECKHSQ
Squirrel monkey       RHPDLSIPQLLRVVQMYNDLLRNCCNTENPPDCYHYAEEKFNETTEKGLKKIQQECKHSQ
Tarsier               RHSDLSIPQLLRITNVYKDLLQNCCKTENPPDCYRHAEDKFNETTEKSLKMVQQECKRFR
Mouse lemur           NHPDLSTPELLRIAHVYKDVLSNCCNTQNPPGCYRRAKDKFNETTEKSHKIVQRECEHFQ
Bushbaby              NHPDLSIPEILRITHEYKALLTSCCNTENPPDCYRHAEDRFKKTTEKSLKIVQRECEHFQ
Chinese tree shrew    RHQDLSIPEILRISQVYEDLLGNCCNTENPQDCYSHAEEKFNETTEKSLKIVKQECDSFQ
Squirrel              RHPELSTPELLRIARVYEDLLADCCNKENPPDCYHQAEDKFNETTERSLRMVQQECEQLQ
Golden hamster        RHNYLSTPELLRITKVYKDLLEDCCSRENPAGCYQHAEDKFNKTTEKSLAMVQQECKLFQ
Kangaroo rat          RHPDLSASTLLQITGMYEDTLGDCYNRENPPDCLR-------------------------
Mouse                 RHPDLSTPELLRITKVYMDFLEDCCSRENPAGCYRHVEDKFNETTQRSLAMVQQECKQFQ
Rat                   RHTELSTPELLRITKVYKDLLEDCCNRKNPLSCYRHAEDKFNETTERSLAMVQQECKQFQ
Naked mole rat        RHPDLSTSELLRTAGVSEDFLRDCCSRETLTDCYRHMKDKFNDTTGESLQMVQQECKHFQ
```

```
Guinea pig           RHPDLSTSELLRITGVYEDFLGDCCSRETPTDCYRHVEGKFNETTGKSLQIVQQECKHFQ
Rabbit               RHQDLSIVELLRIAEVYKNLMENCCSTENPLNCYHSVEEKFNETTEKSLKMVQQECKRFQ
Pika                 RHPHLSILELLRIAEVYEDVIENCCNTEHPPSCYHNVEDKFNETTEKSLKMVQQECKRFQ
Pig                  RHPELSTPELLRIAGVYEDLLRECCNSQTPPDCYKDAEKKFNETTEKSLKIVQRECEHFQ
Alpaca               RHTELSIPELLRIAETYEDLLGECCNTENPPDCYRDAENRFNETTEKSLKIVQQECEHFQ
Bactrian camel       RHTELSIPELLRIAETYEDLLGECCNTENPPDCYRDAENRFNETTEKSLKIVQQECEHFQ
Dolphin              RHPELSTPELLRIAGVYEDLLRECCNTENPPDCYRHAENKFNETTEKSLKIVQRECEHFQ
Killer whale         RHPELSTPELLRIAGVYEDLLRECCNTENPPDCYRHAENKFNETTEKSLKIVQRECEHFQ
Cow                  RHPELSTPELLRIAKVYKDLLKECCNMENPPECYRHAENRFNETTEKSLKIVQRECEHFQ
Sheep                RHPELSTPELLRIAKVYEDLLKECCNMENPPECYRRAENRFNETTEKSLKIVQRECEHFQ
Goat                 RHPELSTPELLRIAKVYEDLLKECCNMENPPECYRRAENRFNETTEKSLKIVQRECEHFQ
Horse                RHPELSTPELLRIARVYEDLLEECCSTENPPDCYGNAEYKFNETTQRSLKIVQRECEHVQ
White rhinoceros     RHPELSTSELLRIARVYKDLLEECCKTENPPDCYGNAEYKFNETTQRSLKIVQRECEHLQ
Cat                  RHPELSTPELLRIAGVYEDLLKECCHTENPPDCYRHAEHRFNETTEKSLKIVQRECERSQ
Dog                  RHPELSTPELLRIAGVYEELLKECCHTENPPDCYRRAEYKFNETTEKSLKIVQRECEHFQ
Ferret               RHLELSVPELLRIAGVYENLLKECCRTENPPDCYRHAEYKFNETTEKSLKIVQRECEHFQ
Panda                RHPELSIPELLRIAGVYEDLLKECCHTENPPDCYRRAENKFNETTEKSLKTVQRECEHFQ
Pacific walrus       RHPELSIPELLRIAGVYEDLLKECCHTENPPDCYRHAEYKFNETTEKSLKIVQRECAHFQ
Weddell seal         RHPELSIPELLRIAGVYEDLLKECCHAENPPDCYRHAEYKFNETTEKSLKIVQRECEHFQ
Megabat              RHPELSVPELLRIASVYDDLLKECCNTKNPPDCYCNAENKFNETTEKSLRIVQRECDHFR
Microbat             RHQELSTPELLRIARVYDDLLRECCNAENPPDCYSTMENKFNETTEKSLKIVQRECEHFQ
Elephant             RH-DLSTPEYLRI------LLEECCKREKTSDCYGHAEEKSN-TTEESLEMVKEECEHFQ
Hyrax                ------------------------------------------------------------
Tenrec               RHQDLSTPEILRIGEVYENLLDDCCKKENPSDCYSHAGEKFNYTTEKSLQMVPQDFEALQ
Opossum              RHPELPFSVLLRIGVMYENLLEMCCKTENPKECYGQAEEEFQKIIAKSWTLVKTECGYFQ
Tasmanian devil      QHLELPHSVLLRIAVIYEELLEKCCKTENPKECYSHGEEEFLNITMKTHAVAKLQCDFFQ
Wallaby              ------------------------------------------------------------
Platypus             KHQKVSGPVIRRVLEKYQDLLEKCCQTADPHQCYSRGEEEFQKILQESQARVQEDCAHFQ


Human                NLGKDGLKYHYLIRLTKIAPQLSTEELVSLGEKMVTAFTTCCTLSEEFACVDNLADLVFG
Chimpanzee           NLGKDGLKYHYLIRLTKIAPQLSTEELVSLGEKMVTAFTTCCTLSEEFACVDNLADLVFG
Bonobo               NLGKDGLKYHYLIRLTKIAPQLSTEELVSLGEKMVTAFTTCCTLSEEFACVDNLADLVFG
Gorilla              NLGKDGLKYHYLIRLTKIAPQLSTEELVSLGEKMVTAFTTCCTLSEEFACVDNLADLVFG
Orangutan            NLGKDGLKYHYLIRLTKIAPQLSTEELVSLGEKMVTAFTTCCTLSEEFACVDNLADLVFG
Gibbon               NLGKDGLKYHYLIRLTKIAPQLSTEELVSLGEKMVTAFTTCCTLSEEFACVDNLADLVFG
Rhesus               NLGKDGLTYQYLIRLTKIAPQLSSEELVSLGKKMVTAFTTCCTLSEEFACVDNLADLVLG
Crab-eating macaque  NLGKDGLTYQYLIRLTKIAPQLSSEELVSLGKKMVTAFTTCCTLSEEFACVDNLADLVLG
Olive baboon         NLGKDGLTYQYLIRLTKIAPQLSSEELVSLGKKMVTAFTTCCTLSEEFACVDNLADLVLG
Marmoset             NLGKDGLKYHYLIRFTKIAPQLSTEELVSLGKKMVTVFTTCCTLSEEFACVENLVDLVLG
Squirrel monkey      NLGKDGLKYHYLIRLTKIAPQLSTEELVSLGKKMVTVFTTCCTLSEEFACVENLADLVLG
Tarsier              NLGKDGLKYHYLMSLTKIAPQLSTEELMFLAKRMVMALTTCCTQSGEFACVDNLVDSALG
Mouse lemur          LWGKDGVKYYYLIRLTKTAPQLSTEELISLGEKMAMALATCCTLSEEFACVDNLVDLVLG
Bushbaby             NWGENGLKYR-L-KITS-GFQLS-------------------------------VDLVLG
Chinese tree shrew   NLGKEDLKYHLLIRMTKKVPQLSTEELIFLGKEMVTVLTTCCTQSEEFACVDNLADLVLG
Squirrel             NLGKDGLKYHSLIELTKIAPQLSTEELISLGKEMVIALTTCCTLSEEFACVDNLADLVLG
Golden hamster       GLEKDALLLHYLVKFTKAAPQLSLEELIYLSKGMTEALTTCCTLSEEFACVDDLADLVLG
Kangaroo rat         ----------SIIKLTKIAPQLPTEQLVYL-------ITTCCTLS--FACVANWADPVLG
Mouse                ELGKDTLQRHFLVKFTKAAPQLPMEELVSLSKEMVAALTTCCTLSDEFACVDNLADLVLG
Rat                  ELGKDALQRHFLVKFTKAAPQLPMEELVSLSKEMVAALATCCTLSDEFACVDNLADLVLG
Naked mole rat       NLGKDGLKHHYLIKLTKIVPQLSTEELISLGKEMVTALTTCYTLSDEFACVD--ADLVLG
Guinea pig           NLGEDGLKYHYLIKLTKIAPQLSTEELISLGKEMVTALTTCCTLSDEFACVDDLADLVLG
Rabbit               NLGKDGLKYHYLIKLTKTAPQLSTEELLSLGQEMVMALSTCCTLSEEFACVDNLMDLVLG
Pika                 NLGKDGLAYYYLIKLTKSAPQLPTEELISLGKETVTALTTCCTLSEEFACVDNLMDLVLG
Pig                  NLGKDDLKYHYLARLTKIAPQLSTDELTFLGKEMMIALATCCTLSEEFACVDNLMDLVLG
Alpaca               NLGKDDLKYYYLIKLTKIAPQLSTEELTFLGKEMVIALTTCCTLSEEFACVDNLMDLVLG
Bactrian camel       NLGKDDLKYYYLIKLTKIAPQLSTEELTFLGKEMVIALTTCCTLSEEFACVDNLMDLVLG
Dolphin              ISGKDDLKYHYLINLTKLAPQLSTEELTFLGKEMVTALTTCCTLSEEFACVDNLVDLVLG
Killer whale         ISGKDDLKYHYLINLTKLAPQLSTEELTFLGKEMVTALTTCCTLSEEFACVDNLVDLVLG
Cow                  NLGKDDLKYHYLINLTKLAPQLSTEELTFLGKEMVMALTTCCTLSEEFACVDNLVDLVLG
Sheep                NLGKDDLKYHYLINLTKLAPQLSTEELTFLGKEMVIALTTCCTLSEEFACVDNLMDLVLG
Goat                 NLGKDDLKYHYLINLTKLAPQLSTEELTFLGKEMVIALTTCCTLSEEFACVDNLMDLVLG
Horse                NLGKDDLKYHYLIKFTKIAPQLSTEELTFLGKEMVTALTTCCTLSEEFACVDNLVDLVIG
White rhinoceros     NLGKDGLKYHYLIKLTRIAPQLSTEELTFLGQEKAAALTTCCTLSEEFACVDNLVDLVLG
Cat                  NLGKEDLKYYYLIKLTKIAPQLSTEELTFLGKEMVTALTTCCTLSEEFACVDNLMDLVLG
Dog                  NLGKDDLKYYYIVKLTKIAPQLSTEELTFLGKEMVAALTTCCTLSEEFACVDNLVDLVLG
```

```
Ferret                NLGKDDLKYYYLIKLTKIAPQLSTEELTFLGKEMVTALATCCTLSEEFACVDNLMDLVLG
Panda                 NLGKDDLKHYYLIKLTKIAPQLSTEELTFLGKEMVTALTTCCTLSEEFACVDNLMDLVLG
Pacific walrus        DLGKDDLKYYYLIKFTKIAPQVSTEELTFLGKEMVTALATCCTLSEEFACVDNLMDLVLG
Weddell seal          NLGKDDLKYYYLIKLTKIAPQLSTEELTFLGKEMVTALATCCTLSEEFACVDNLMDLVLG
Megabat               NLGKDDLKYHYLIKLTKIAPQLSTEELTFIGEEMVTALTTCCTLSEEFACVDNLAKLIFG
Microbat              NLREDVLKY---------------------------------------------------
Elephant              ------------------------------------------------------------
Hyrax                 ----------------------------------------------FACVDHLAKLFLG
Tenrec                NLWKEDLKYHHLVKVKKMTHQVSTKKLSLFSKDTEPALAMCCAQSEEFACMDNLMNLVLG
Opossum               NLGEEGFLNQCLLPLTKKAPQLPPQELISHSKKLASFFAKCCPLGEEFACVDNAVSLTIG
Tasmanian devil       QLGEEGFFTQCLLFLTKKAPQLSSQELISHSQKLVTSFSKCCFLEEKFACVDNLAGLVLG
Wallaby               ----------CLLVLTKRAPQLPPQELISQMQKFHASFTKCCPLGEEFARVHH-VHLILG
Platypus              KLGEDLYRQKYAISFTKQAPELPLTELTVHVERKTNDAAHCCMQPDETACIERSANLLYG


Human                 ELCGVNENRTINPAVDHCCKTNFAFRRPCFESLKADKTYVPPPFSQDLFTFHADMCQSQN
Chimpanzee            ELCGVNENRTINPAVDHCCKTNFAFRRPCFESLKADKTYVPPPFSQDLFTFHADMCQSQN
Bonobo                ELCGVNENRTINPAVDHCCKTNFAFRRPCFESLKADKTYVPPPFSQDLFTFHADMCQSQN
Gorilla               ELCGVNENRTINPAVDHCCKTNFAFRRPCFESLKADKTYVPPPFSQDLFTFHADMCQSQN
Orangutan             ELCGVNENRTINPAADHCCKTKFAFRRPCFESLKADKTYVPPPFSQDLFTFHADMCQSQN
Gibbon                ELCGVNENRTINPAVDHCCKTNFAFRRPCFESLKADKTYVPPPLSQDIFTFHADMCQSQN
Rhesus                ELCGVNENRTINPAVDHCCKTNFAFRRPCFESLKADKTYVPPPFSQDLFTFHADMCQSQN
Crab-eating macaque   ELCGVNENRTINPAVDHCCKTNFAFRRPCFESLKADKTYVPPPFSQDLFTFHADMCQSQN
Olive baboon          ELCGVNENRTINPAVDHCCKTNFAFRRPCFESLKADKTYVPPPFSQDLFTFHADMCQSQN
Marmoset              ELCGVNENRTINPAVDHCCKSNFAFRGPCFESLTTHKTYVPPPFSQDLFTFHPDMCQSQN
Squirrel monkey       ELCGVNENRTINPAVDHCCKSNVAFRGPCFESLTAHKTYVPPPFSQDLFTFHTDMCQSQN
Tarsier               ELCGVNLNRTINPAVDRCCRTNFAFRTPCFKDLKTDKTYVPPPTSQDLFTFHTDLCQAQN
Mouse lemur           ELCGINENRTINPAVDHCCKTNFAFRRHCFEGLKADETYVPPTTTKDLFTFNADLCQAQN
Bushbaby              ELCGVHKNRTINPAVDHCCRTNFAFRRYCFEGLEVDKTYVPPPTSKDLFTFPADLCQAGN
Chinese tree shrew    EFCGINKNRTINPAVDHCCLTNPAFRRHCFESLEADKTYVPPPTSRDLFIFHTDLCQAPN
Squirrel              ELCGINKNRTINPAVDHCCKANFAFRRLCFKELKGDEMYEPPS-SQGFFTSCVDWCQAQN
Golden hamster        DLCGVNENRTINPAVDHCCRADFAFRRHCFEHLEADTTYALSSVSALISALRSDWCQNHN
Kangaroo rat          ELCGINENQTINPALDHCCKANFAFRRHCFGGLEADKMYVPPLVSEDSFAFHDEWCMTQN
Mouse                 ELCGVNTNRTINPAVDHCCKTDFAFRRHCFEHLKADTTYELPSVSALVSALHTDWCQPRK
Rat                   ELCGINKNRTINPTVDHCCRADFAFRRPCFEHLKADTTYALPSVSALVSALRADWCQPLK
Naked mole rat        ELCGINENQTINPAMDHCC--NFAFRRHCFEGLKADKTYVPPPLSQDLPVFPVDWCQSQN
Guinea pig            ELCGINENRTINPAVDHCCTANFAFRRPCFESLKPDKTYIPPPLSQDLSVFPVDWCQSQN
Rabbit                ELCGIKENRTINPAVDHCCQTNFAFRRPCFEGLKADQTYVPPPTSQDFFAFPLDWCQDQK
Pika                  DLCGVNENRTINPAVGRCCQTSFAFRRPCLEGLEADPTYVPPPIVQDVFAFRADWCQTQD
Pig                   ELCGINKNRSINPTVDHCCKTNFAFRRSCFEGLEADKTYVPLPPSQGLFTFPPDLCQAHN
Alpaca                QLCGINENRNINPAVDHCCKTNFAFRRSCFEGLKADKAYVPLPTSQGLFTFHTDLCQAHN
Bactrian camel        ELCGINEYHNINPAVDHCCKTNFAFRRSCFEGLKADKAYVPPPTSQGLFTFHTDLCQAHN
Dolphin               ELCGINENRSINPAVDHCCKTNFAFRRSCFEGLEADKTYVPPPTSQGLFTFHTDLCQAHN
Killer whale          ELCGINENRSINPAVDHCCKTNFAFRRSCFEGLEADKTYVPPPTSQGLFTFHTDLCQAHN
Cow                   ELCGINENRNINPAVDHCCKTNFAFRRSCFESLEADKTYVPPSTSQGLFTFHADLCQAHN
Sheep                 ELCGINENRNINPAVDHCCKTNFAFRRSCFESLEADKTYVPPSTSQGLFTFHADLCQAHN
Goat                  ELCGINENRNINPAVDHCCKTNFAFRRSCFESLEADKTYVPPSTSQGLFTFHADLCQAHN
Horse                 ELCGINENRSINPAVDHCCKTDFAFRRPCFEGLEADKTYVPPSTSQGLFTFHADWCQAHK
White rhinoceros      ELCGINENRSINPAVDHCCKTDFAFRRPCFEGLEADKTYVPPSTSQGLFTFHTDLCQAHK
Cat                   ELCGINENRNINPDVDHCCKTNFAFRRSCFEGLEADKAYVPPSTFQGLFTFHTDLCQAHK
Dog                   ELCGINENRNINPDVDHCCKTNFAFRRPCFEGLEADKTYVPPSTSQDLFTFHTDLCQAHN
Ferret                ELCGLNENRNINPDVDHCCKTNFAFRRPCFEGLEADKTYKPPCTPQDLFVFHTDLCQSHN
Panda                 ELCGINENRNINPDVDHCCKTNFAFRRPCFEGLEADKTYVPPSTSQDLFTFHTDLCQAHN
Pacific walrus        ELCGINENRNINPDVDHCCKTNFAFRRPCFEGLEADKTYVPPSTPQDLFTFHTDLCQAHN
Weddell seal          ELCGINENRDINPDVDHCCKTNFAFRRPCFEGLEADKTYVPPSTPQDLFTFHTDLCQAHN
Megabat               ALCRRHEAEPINDGVGHCCDDSYAFRKPCFDDLHVDRTYISPPLSCDLSALE-ELCKAE-
Microbat              EICGVNGNRTINPAVDHCCKTNFAFRRHCFEALEADKTYVPPSASQGLFTFHTDLCQAHN
Elephant              ------------------------------------------------------------
Hyrax                 VLCRRHEAKPINAGVGHCCDASYAFRKPCFDDLSADETYIAPSLSCDVISLKEDLCKPQE
Tenrec                ELCGVIRNPTVNRAVDQCSKSHFAFRG-CLENLKQDGKHVPPSTTQGWSTFH-DWYQAQN
Opossum               ELCGINRNRTINPGVDNCCKSSYAFSSMCFKDLTTDETYTSPTFLPELFTFNLTLCQGK-
Tasmanian devil       ELCGINKNRTINPTVDNCCASSSAFRPLCFNDLPEDETYTPPTFLPEMFTFNISLCQ-E-
Wallaby               EICGVNKN-TINPNVDNCCKSSLVFRSWCFNNLTADETFIPPTFPTEVFTFNITLCQ-K-
Platypus              QICGFFANRSINPGVTNCCKS-FAFKEPCFPSLKVDETYTPPAFSPELFTPNEDWCQLPD
```

```
Human                  EELQRKTDRFLVNLVKLKHELTDEELQSLFTNFANVVDKCCKAESPEVCFNEESPKIGN
Chimpanzee             EELQRKTDRFLVNLVKLKHELTDEELQSLFTNFANVVDKCCKAESPEVCFNEESPKIGN
Bonobo                 EELQRKTDRFLVNLVKLKHELTDEELQSLFTNFANVVDKCCKAESPEVCFNEESPKIGN
Gorilla                EELQRKTDRFLVNLVKLKHELTDEELQSLFTNFANVVDKCCKAESPEVCFNEESPKIGN
Orangutan              EELQRKTDRFLVNLVKLKHELTDEELQSLFTNFANAVDKCCKAESPEVCFNEESPKIGN
Gibbon                 EELQRKTERSLVNLVKLKHELTDEKLQSLFTNFTNAVDKCCQAESPEDCFNEESPKIGN
Rhesus                 EELQRKTDRFLVNVVKLKHELTDEELQSLFTNFTNAVEKCCKAESPKVCFNEESPKIGN
Crab-eating macaque    EELQRKTDRFLVNVVKLKHELTDEELQSLFTNFTNAVEKCCKAESPKVCFNEESPKIGN
Olive baboon           EELQRKTDRFLVNVVKLKHELTDEELQSLFTNFTNAVEKCCKAESPEVCFNEESPKIGN
Marmoset               EELQKKTDRFLVNIVKLKYEVANEELQSLLTNFGNAVEKCCKAEKDEVCFNVESPKIVT
Squirrel monkey        EELQKKTDRFLVNIVKLKYEVANEELQSLLTNFGNAVEKCCKAEKDEVCFNKESPKLVT
Tarsier                EELQRKKDRFLVNLVKRKPGLTDEKLLTLFTNVTLVVEKCCKAEGPEACFNEEGPKIDN
Mouse lemur            EELQRKKDRVLVDLARRR----------------------------------EGPKIGS
Bushbaby               EELQRRKDRFLVNLVKRRRELTAEQLQTLFTRVTKAVEKCCEAEAPEACFKEETEPVLV
Chinese tree shrew     EELQKKKDRFLVNLVKLKPGFADQELRSLFLTFTAAVEKCCKAQEPEACFNEEVSKLGN
Squirrel               EELQRKKDRFLVNLVKLKPGLTYEELQSLFTSFTNMAEKCCKMQEPEACFLEEGPKIGN
Golden hamster         EDLQNKKHRFMVNLVKWMPEITAKERLCLFTKFTAVGEKCEEVQEPETCFRPEDPGTVP
Kangaroo rat           EELQSMKNRFLIDLVKWKPALTQEKLRSLF-----------------------------
Mouse                  EDLQNKKHRFLVNLVKWMPGITDEEWLCLFTKFTAAREECSEVQEPESCFSPESSKTG-
Rat                    EDLQNKRHRFLVNLVKWMPEITDEERLCLFTKFTAAGEECGNIQKPEACFSPESSKTG-
Naked mole rat         EVPQGKKYRFLIKLVRLKPQLTDEELLSLLTNFTSAVEKCCKVPEPEACFHEE------
Guinea pig             EELQGKKYRFLIQLVRLKLQLADKELLSLLTQFITGVKKCCTAPEPEACFHENTSMSGN
Rabbit                 EELQRKKDRFLVNLVKLKPELPDEELQSLFTNFTDTLEKCCNAKGPEACFNQEHLKIGD
Pika                   EELQRKKDRFLVNLVKLKPELANQEVQSLFINFTDTLESCCKAKAPEACFNQERPKIGS
Pig                    EELQRKKDRFLVNLVKLKPELTEEALRSSLIEFTNVVVKCCQAEGPEACFEEEGPKLAA
Alpaca                 EELQRKKDRFLVNLVKWKPELREDKLQSLLTDFTKVVATCCKAEGPAACFSEEGPKLAA
Bactrian camel         EELQRKKDRFLVNLVKWKPELTEDKLRSLLTDFTKVVATCCKAEGPAACFSEEGPKLAA
Dolphin                EELQRKKDRFLVNLVKL-PELT-VELQSLLMDFTNVVKKCCQAQGPEACFKEEGPKLSA
Killer whale           EELQRKRDRFLVNLVKLKPELTRVELQSLLVDFTNVVKKCCQAQGPEACFKEEGPKLSA
Cow                    EELQRKKDRFLVNLVKLKPELAGEELWSLLADFTNVVEKCCKAQEPEACFKEESPKLAA
Sheep                  EELQRKKDRFLVNLVKLKPELAGEELWSLLADFTNVVEKCCKAQEPEACFKEESPKLAA
Goat                   EELQRKKDRFLVNLVKLKPELAGEELWSLLADFTTVVEKCCKAQEPEACFKEESPKLAA
Horse                  EELQRKKDRFLVNLVKLKPELGGEALQSLPTDFSNVVEKCCKAEGPEACFSEE------
White rhinoceros       EELQRKKDRFLVDLVKLKPEFRDEELQSLLTDFSNVVETCCKAEGPETCFNEEGPKLAA
Cat                    EELQRKKDRFLVNVVKLKPELPDVELRSLLTDFTNVVVKCCQAEGPEACFNEEGPQTPP
Dog                    EELQRKKDRFLVNLVKRKPELTEEELRSLLADFSNVVVTCCKAEGPEACFNEELNKT--
Ferret                 EELQRKKDRFLVNVVKRGPELTEAVLRPLLSDFTDVVAKCCKAEDPETCFREEGPKVAA
Panda                  EELQRKKDRFLVNLVKRKPELTDEALRSLLTDFTNVVVKCCKAEGPEACFNEEGPKL--
Pacific walrus         EELQKKKDRFLVNLVKRRPELTDEALRSLLTAFTDVVVKCCKAEGPEACFNEEGPKVTA
Weddell seal           EELQKKKDRFLVNLVKQRPELTDEALKSLLTDFTDVVVKCCKAEGPEACFNEEGPKVTA
Megabat                EEFQ--TEKFLVNLVKLKPELTNEELWSLLTDSANAVDRCCKAERPEACWNEEG-KIGS
Microbat               EELQRKKDRFLVNLVKLKPELTDEELRSSLTGFTNLVDKCCKAEGPEACFNEEDSDSSH
Elephant               -----------------------------------------------------------
Hyrax                  DKLQSKAE-FLVELVKLMPPHT-EELWSLITDFTNVLEKCCKLEGPEASFSDE------
Tenrec                 GALQSKKERLFVRSVKMKSQRPD-ELPSWLTAFAPGVNRCGE-EGPEPCFNEECPTGSQ
Opossum                EEQQKKRRELMVHLLQQKPHLGVDQVMPLLSDFSKMTD-CCEVEEKETCFKEEHSKLYN
Tasmanian devil        MELQRKRRELIVNLVRQKPVLETQQVMQMFSDFPTLIN-CCKTEERESCFKEEHSKLYN
Wallaby                TEQQRKR-MLIMNLVKQKPLLAAKQVIQVLLDFS-----CCEAEERVTCFKEE------
Platypus               MELQRKKLKLLINLVKHKPQTPDDQLQTALTDFSDVVKKCCQASGPGACFVAEGPNLIA
```

Legend:

: Positively selected site in the whole phylogeny

: Lineage-specific positively selected site

Table S1. List of mammalian species.

Common name	Scientific name
Alpaca	*Vicugna pacos*
Armadillo	*Dasypus novemcinctus*
Bactrian camel	*Camelus ferus*
Black flying fox	*Pteropus alecto*
Bonobo	*Pan paniscus*
Bushbaby	*Otolemur garnettii*
Cape golden mole	*Chrysochloris asiatica*
Cat	*Felis catus*
Chimpanzee	*Pan troglodytes*
Cow	*Bos taurus*
Crab-eating macaque	*Macaca fascicularis*
Dog	*Canis lupus familiaris*
Dolphin	*Tursiops truncatus*
Elephant	*Loxodonta africana*
Ferret	*Mustela putorius furo*
Gibbon	*Nomascus leucogenys*
Goat	*Capra hircus*
Golden hamster	*Mesocricetus auratus*
Gorilla	*Gorilla gorilla*
Ground squirrel	*Spermophilus citellus*
Guinea Pig	*Cavia porcellus*
Hedgehog	*Erinaceus europaeus*
Horse	*Equus caballus*
Human	*Homo sapiens*
Hyrax	*Procavia capensis*
Kangaroo rat	*Dipodomys ordii*
Killer whale	*Orcinus orca*
Lesser hedgehog tenrec	*Echinops telfairi*
Manatee	*Trichechus inunguis*
Marmoset	*Callithrix jacchus*
Megabat	*Pteropus vampyrus*
Microbat	*Myotis lucifugus*
David's Myotis	*Myotis davidii*
Mouse	*Mus musculus*
Mouse Lemur	*Microcebus murinus*
Naked mole rat	*Heterocephalus glaber*
Olive Baboon	*Papio anubis*
Opossum	*Monodelphis domestica*
Orangutan	*Pongo abelii*
Panda	*Ailuropoda melanoleuca*
Pig	*Sus scrofa*
Pika	*Ochotona princeps*
Platypus	*Ornithorhynchus anatinus*
Rabbit	*Oryctolagus cuniculus*
Rat	*Rattus norvegicus*
Rhesus macaque	*Macaca mulatta*
Rhinoceros	*Ceratotherium simum*
Sheep	*Ovis aries*
Shrew	*Sorex araneus*
Sloth	*Choloepus hoffmanni*
Squirrel	*Ictidomys tridecemlineatus*
Squirrel monkey	*Saimiri boliviensis*
Star-nosed mole	*Condylura cristata*
Tarsier	*Tarsius syrichta*
Tasmanian devil	*Sarcophilus harrisii*
Tree Shrew	*Tupaia belangeri*
Wallaby	*Macropus eugenii*
Walrus	*Odobenus rosmarus*
Weddell seal	*Leptonychotes weddellii*

Table S2. Positively selected sites in the human and chimpanzee lineages.

Gene	Lineage	Codon	Ancestral AA	Human/chimpanzee AA	Pr[a]	Other methods[b]
ALB	Human	183	Glu	Lys	0.765	
		314	Leu	Ile	0.776	MEME
	Chimpanzee	49	Ile	Val	0.764	
		107	Thr	Lys	0.950	MEME-BEB
		156	Glu	Gly	0.913	MEME-BEB
		166	Ile	Val	0.908	MEME
AFP	Human	42	Asn	Ser	0.875	MEME
		44	Thr	Ala	0.873	MEME
	Chimpanzee	100	His	Arg	0.842	
AFM	Human	29	Val	Ile	0.777	
		183	Ala	Val	0.876	
		202	Phe	Leu	0.887	MEME
	Chimpanzee	200	Phe	Leu	0.829	
		241	Gln	His	0.906	

[a] Posterior probability of $\gamma > 1$ as detected by gammaMap.
[b] Other methods that identified the same codon as positively selected

Table S3. Nucleotide diversity and Tajima's D for the albuminoid genes.

Gene	Population	S[a]	Θ_W (x 10^{-4})		Π (x 10^{-4})		Tajima's D	
			value	rank[b]	value	rank[b]	value	rank[b]
GC	YRI	244	7.15	0.63	7.72	0.63	0.26	0.54
	CEU	142	4.15	0.41	4.23	0.32	0.07	0.26
	CHBJPT	122	3.57	0.52	5.77	0.61	2.02	0.77
ALB	YRI	45	4.91	0.25	5.94	0.39	0.65	0.76
	CEU	32	3.48	0.26	5.57	0.51	1.81	0.89
	CHBJPT	28	3.04	0.39	5.67	0.60	2.55	0.91
AFP	YRI	90	7.57	0.69	7.02	0.54	-0.23	0.25
	CEU	57	4.78	0.56	4.52	0.36	-0.17	0.20
	CHBJPT	32	2.68	0.30	4.73	0.47	2.29	0.85
AFM	YRI	63	6.03	0.44	5.85	0.38	-0.09	0.33
	CEU	36	3.43	0.26	4.92	0.42	1.31	0.73
	CHBJPT	22	2.10	0.17	3.66	0.32	2.13	0.80

[a] Number of segregating sites
[b] percentile rank relative to a distribution of ~3000 randomly selected genes

Table S4. Variant vitamin D-binding proteins.

Variant Name	rs4588	rs7041	Frequency		
			YRI	CEU	CHBJPT
GC*1F	Thr (C)	Asp (T)	0.89	0.22	0.50
GC*1S	Thr (C)	Glu (G)	0.08	0.58	0.25
GC2	Lys (A)	Asp (T)	0.03	0.20	0.25
